# Synthesis, Characterization and Bioassay of Novel Substituted 1-(3-(1,3-Thiazol-2-yl)phenyl)-5-oxopyrrolidines

**DOI:** 10.3390/molecules25102433

**Published:** 2020-05-22

**Authors:** Birutė Sapijanskaitė-Banevič, Božena Šovkovaja, Rita Vaickelionienė, Jūratė Šiugždaitė, Eglė Mickevičiūtė

**Affiliations:** 1 Department of Organic Chemistry, Kaunas University of Technology, Radvilėnų Rd. 19, LT-50254 Kaunas, Lithuania; birute.sapijanskaite@ktu.lt (B.S.-B.); bsergeevna@gmail.com (B.Š.); 2 Department of Veterinary Pathobiology, Veterinary Academy, Lithuanian University of Health Sciences, Tilžės Str. 18, LT-47181 Kaunas, Lithuania; siujur@gmail.com; 3 Department of Information Systems, Kaunas University of Technology, Studentų Str. 50, LT-51368 Kaunas, Lithuania; egle.mickeviciute@ktu.lt

**Keywords:** biological activity, heterocycles, thiazoles, pyrrolidinones, triazoles

## Abstract

Thiazole derivatives attract the attention of scientists both in the field of organic synthesis and bioactivity research due to their high biological activity. In the present study, thiazole ring was obtained by the interaction of 1-(4-(bromoacetyl)phenyl)-5-oxopyrrolidine-3-carboxylic acid with thiocarbamide or benzenecarbothioamide, as well as tioureido acid. A series of substituted 1-(3-(1,3-thiazol-2-yl)phenyl)-5-oxopyrrolidines with pyrrolidinone, thiazole, pyrrole, 1,2,4-triazole, oxadiazole and benzimidazole heterocyclic fragments were synthesized and their antibacterial properties were evaluated against Gram-positive strains of *Staphylococcus aureus*, *Bacillus cereus*, *Listeria monocytogenes* and Gram-negative *Pseudomonas aeruginosa*, *Escherichia coli* and *Salmonella enterica enteritidis*. The vast majority of compounds exhibited between twofold and 16-fold increased antibacterial effect against the test-cultures when compared with *Oxytetracycline*.

## 1. Introduction

**Figure 1:**
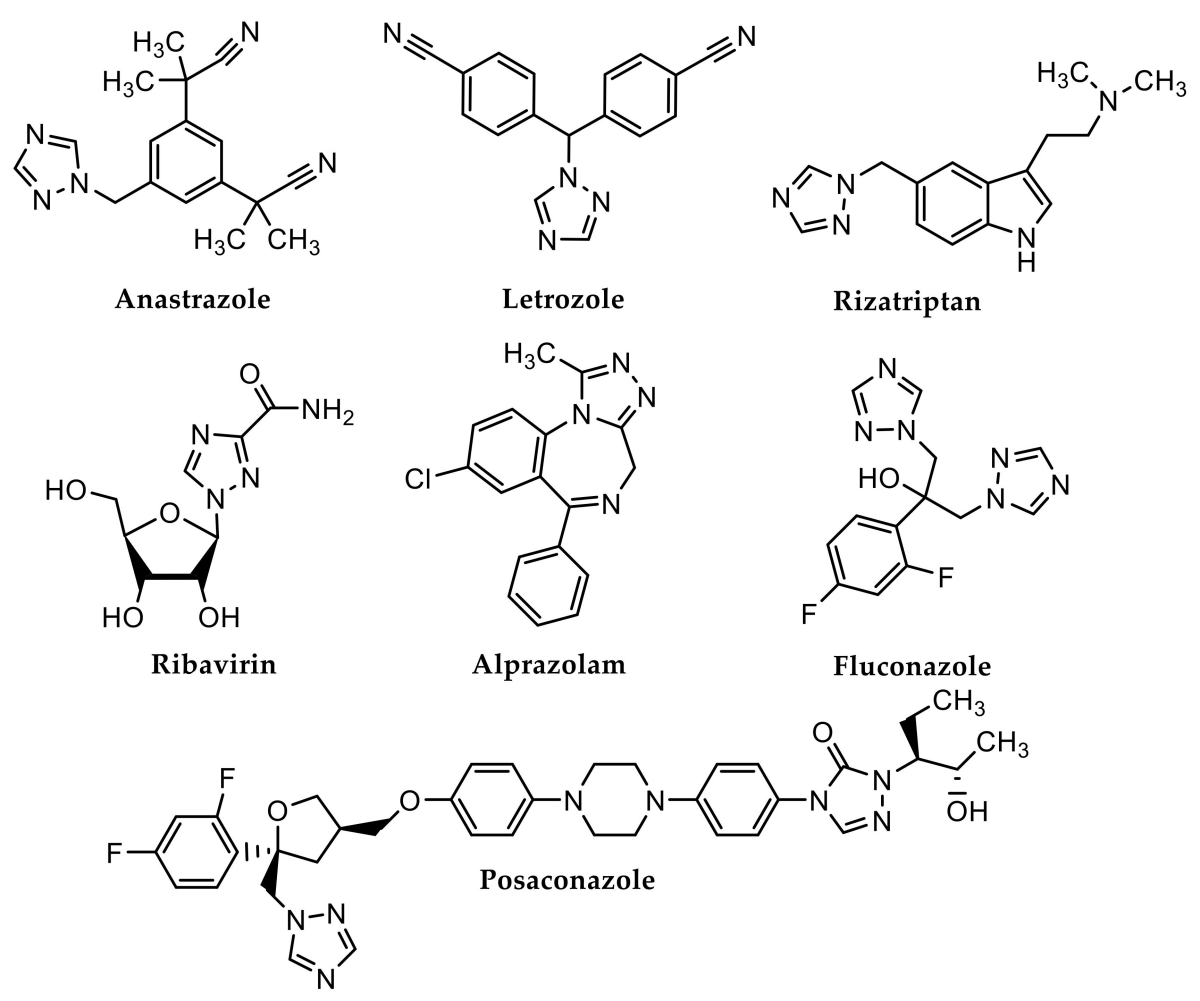
Pharmaceuticals with 1,2,4-triazole scaffold in the molecule.

Heterocyclic compounds are one of the main groups of organic compounds possessing a wide range of applications in various areas of science and high technologies. Recently, greater attention has been paid to heterocyclic compounds such as azoles. The nitrogen-containing five-membered heterocycles–pyrrole, diazole, triazole, thiazole, thiadiazole, oxadiazole, benzimidazole demonstrate a wide diversity of biological properties. Thiazoles are commonly reported as active antimicrobial [1,2,3,4,5,6,7,8], anti-tuberculous [9,10,11,12], anti-HIV [13], antiviral [14,15], antihistamine [16,17], antipyretic [18], antitumor [3,19], antidepressant [20], fungicidal [21,22] property bearing compounds. 2-Aminothiazoles has found application in agriculture as herbicides and fungicides [23,24]. Pyrrole nucleus has been incorporated into a wide variety of therapeutically important medication candidates. For example, the pyrrole ring is present in the structure of the anti-cancerous drug *Sunitinib* [25,26], which is used for the treatment of several types of cancer. Compounds with a pyrrolidinone moiety in the structure show inhibition of human carbonic anhydrase [27,28]. Compounds containing a 1,2,4-triazole scaffold demonstrate different pharmacological actions such as analgesic [29,30], antiviral [31], anti-inflammatory [32], anticonvulsant [33,34], anticancer [35], antidepressant [36], antibacterial [37,38], antifungal [37,39], and are used as various pharmaceuticals. They have been approved as drugs such as Anastrazole, Letrozole, Rizatriptan, Ribavirin, Alprazolam, Fluconazole and Posaconazole (Figure 1). Therefore, many researchers looking for new medicines are still focused on the synthesis of 1,2,4-triazole derivatives. Benzimidazole is an important heterocyclic compound with the fused-ring system in the structure. Because of its similarity to the structure of purine bases, benzimidazole derivatives can compete with them in the course of bacterial membrane biosynthesis, thereby stimulating the death of bacteria [40,41].

This study focused on the synthesis of new, potentially biologically active 1,3-disubstituted 5-oxopyrrolidines with pyrrolidinone, thiazole, pyrrole, 1,2,4-triazole, oxadiazole, benzimidazole and quinoxaline heterocyclic fragments in the structure, and resulted in the discovery of compounds with significantly increased antibacterial activity.

## 2. Results and Discussion

### 2.1. Chemistry

**Scheme 1:**
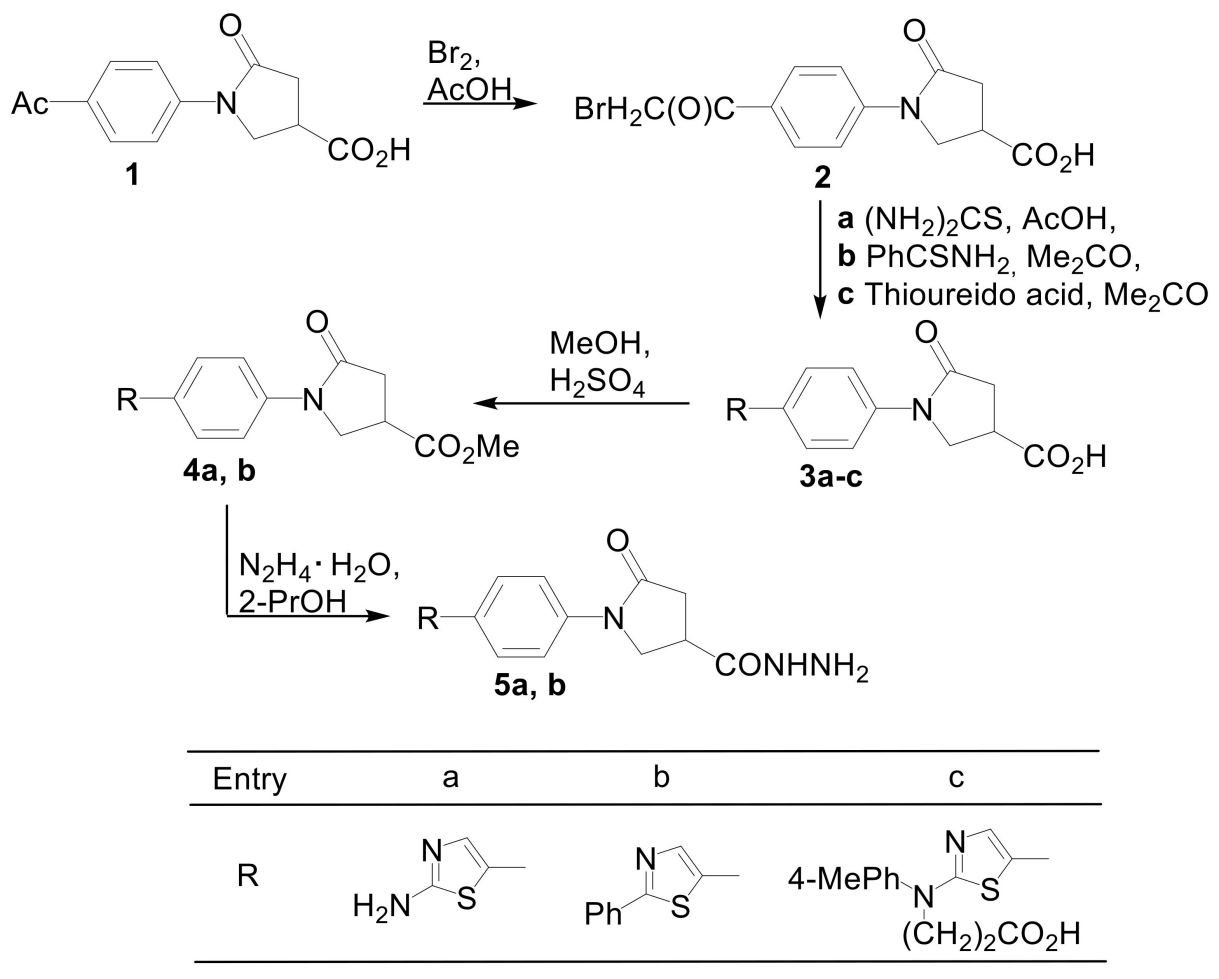
Synthesis of disubstituted pyrrolidinone derivatives **2–5**.

In this work, the initial compound **1** was prepared from 4-aminoacetophenone and itaconic acid by a known method [42]. In order to obtain 2,5-disubstituted thiazole derivatives **3a–c**, compound **1** was brominated with Br_2_ in acetic acid at room temperature (Scheme 1), and then used in the reactions with thiocarbonyl compounds. The bromination reaction resulted in the formation of α-bromocarbonyl compound 1-(4-(2-bromoacetyl)phenyl)-5-oxopyrrolidine-3-carboxylic acid (**2**). Cyclocondensation of α-bromoacyl derivative 2 with the corresponding thioamide, i.e., thiocarbamide, benzenecarbothioamide or thioureido acid, under different conditions was carried out to obtain the desired outcomes **3a–c**. The **a** reaction was performed in acetic acid at 60 °C; in the cases of **b** and **c**, the reaction was carried out in refluxing acetone. A comparison of the ^1^H and ^13^C-NMR spectra of compounds **3a–c** revealed the characteristic signals of the formed substituted thiazole moieties. In the spectra of compound **3a**, the singlets at 6.96 (S–C=CH) and 7.07 (NH_2_, ^1^H-NMR) ppm, and the resonance lines at 100.9 (C–S), 138.1 (S–C=*C*H), 168.2 (C=N, ^13^C-NMR) ppm prove the presence of thiazole heterocycle in the molecule. The characteristic signals in the ^1^H (8.20 ppm, S–C=CH) and ^13^C (114.0, 154.7, 166.9 ppm, C–S, S–C=*C*H, C=N) NMR, as well as additional resonances in the aromatic field of the corresponding spectrum of **3b**, provide the evidence of new 2-phenyl thiazole fragment. The formation of the 2-carboxyethyl-4-methylanilino moiety in compound **3c** was confirmed by the presence of triplets at 2.40 (*J* = 7.9 Hz, NCH_2_C*H*_2_CO) and 4.10 (*J* = 7.9 Hz, NC*H*_2_CH_2_) ppm in the ^1^H-NMR spectrum. The signals of protons of the *p*-substituted benzene cycle resonated as two doublets at 7.26 and 7.32 ppm and the signal arising at 2.32 ppm indicated the presence of the methyl group. All NMR spectra of the synthesized compounds are given in Supplementary Materials.

Compounds **5a**,**b** were prepared following the standard synthesis route, i.e., 5-oxopyrrolidine-3-carboxylic acids **3a**,**b** were esterified with methanol in the presence of a catalytic amount of sulfuric acid to obtain esters **4a**,**b**. The prepared esters were easily converted to the corresponding acid hydrazides **5a**,**b**, when treated with hydrazine hydrate in refluxing propan-2-ol.

**Scheme 2:**
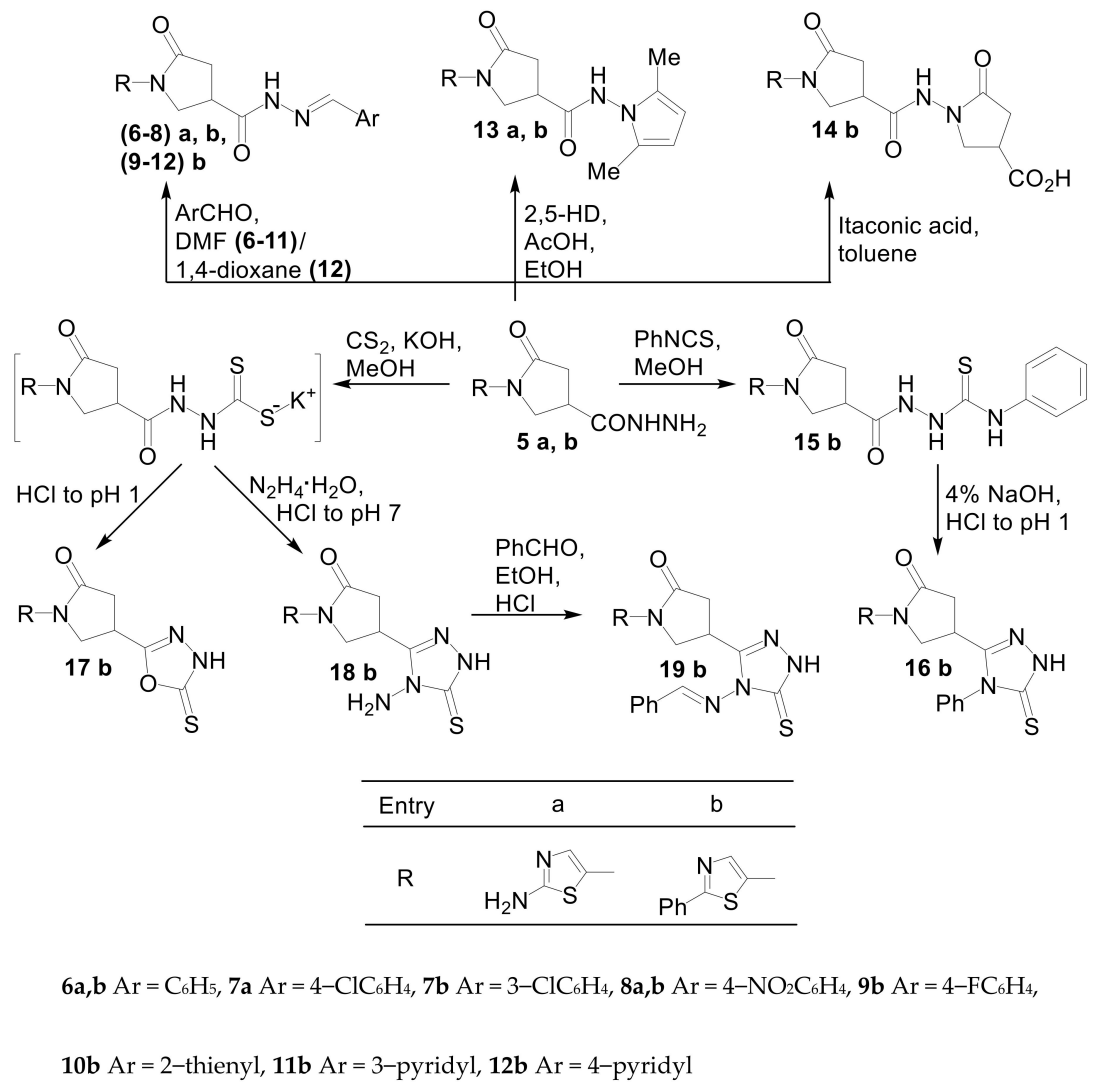
Synthesis of compounds **6–19**.

The condensation of acid hydrazides **5a**,**b** with aromatic aldehydes in dimethylformamide or 1,4-dioxane afforded hydrazone-type compounds (**6–8**)**a**,**b**, and (**9–12**)**b** in good to excellent yield (62–99%, Scheme 2; the yields of all synthesized compounds are given in Table 1).

The synthesized hydrazones in DMSO-*d*_6_ solution, due to the restricted rotation around the CO-NH bond, exist as a mixtures of *E/Z*-amide conformers with the prevailing *Z* conformational structure [43,44,45]. However, geometric isomers are also possible due to the presence of the N=C double bond. The academic literature indicates that hydrazones obtained from acid hydrazides and aromatic aldehydes favour the sterically less-hindered and more stable geometric *E-*geometrical isomer [46,47,48].

The reaction of acid hydrazides **5a**,**b** with hexane-2,5-dione (2,5-HD) resulted in the formation of 2,5-dimethylpyrrole derivatives **13a**,**b**, as expected. Using the method of the formation of pyrrolidinone ring from aromatic amines and itaconic acid, hydrazide **5b** as an amino group-containing compound was reacted with this dicarboxylic acid. The reaction was performed in toluene at reflux, and the synthesized compound **14b** containing two pyrrolidinone rings connected by the amide bond was proved by double sets of the protons (^1^H-NMR) of the 2COCH_2_, 2CH and 2NCH_2_ groups, which gave rise in the ranges of 2.54–2.88, 3.25–3.42 and 3.55–4.16 ppm, respectively. In the ^13^C-NMR spectrum of **14b** spectral lines at 31.3, 33.6, 34.2, 35.5, 49.7 and 50.3 ppm have been attributed to the CO*C*H_2_, CH and NCH_2_ groups of two pyrrolidinone rings, and resonances at 170.9, 171.4, 171.8 and 174.6 ppm approve the presence of four carbonyl groups in this structure. NMR spectral data of compound **14b** showed the presence of only one isomer in the DMSO-*d*_6_ solution. Unfortunately, attempts to grow single crystals suitable for stereochemical assignment by X-ray structural analysis were unsuccessful.

Thiosemicarbazide **15b** was synthesized by treatment of acid hydrazide **5b** with phenyl isothiocyanate in methanol. Singlets at 9.58, 9.68, 9.88, 10.12 and 10.22 ppm in the ^1^H-NMR spectrum of the compound are characteristic of the 3NH of the thiosemicarbazide moiety, and six additional resonance lines of the newly attached phenyl fragment in the ^13^C-NMR spectrum of **15b** provide clear evidence of the compound structure. 2-(5-Oxo-1-(4-(2-phenylthiazol-5-yl)phenyl)pyrrolidine-3-carbonyl)-*N*-phenylhydrazine-1-carbothioamide (**15b**) was then converted to 1,2,4-triazole **16b** by a base catalyzed intramolecular dehydrative cyclization reaction.

The reaction of hydrazide **5b** with carbon disulfide in methanol under basic conditions (KOH/methanol) gave the intermediate, the potassium dithiocarbazate salt, which under the action of concentrated hydrochloric acid was cyclized to 5-thioxo-1,3,4-oxadiazole **17b.** The treatment of potassium carbodithioate with hydrazine monohydrate afforded 4-aminotriazole **18b**. The condensation of one equivalent of compound **18b** containing free amino group with two equivalents of benzenecarbaldehyde in refluxing ethanol containing a catalytic amount of concentrated hydrochloric acid yielded the Schiff base **19b**. The characteristic changes in the chemical shift of the resonances of the triazole moiety were observed because of the changed influence of the substituent at the nitrogen atom of the C–N–C=S fragment. In the case of **19b**, the carbon atom of the C=S group resonated at 162.9 ppm, and the C=N–NH carbon atom peaked at 162.0 ppm in ^13^C-NMR spectrum. Additional spectral lines were observed in the aromatic region and were assigned to the carbon atoms of the newly incorporated benzene ring and formed azomethine group. The ^1^H-NMR spectrum of 1,2,4-triazole derivative **19b** showed characteristic singlets at 10.14 ppm (N=CH) and 13.97 (NH), and the multiplet of the benzene rings integrated for 14 protons.

**Scheme 3:**
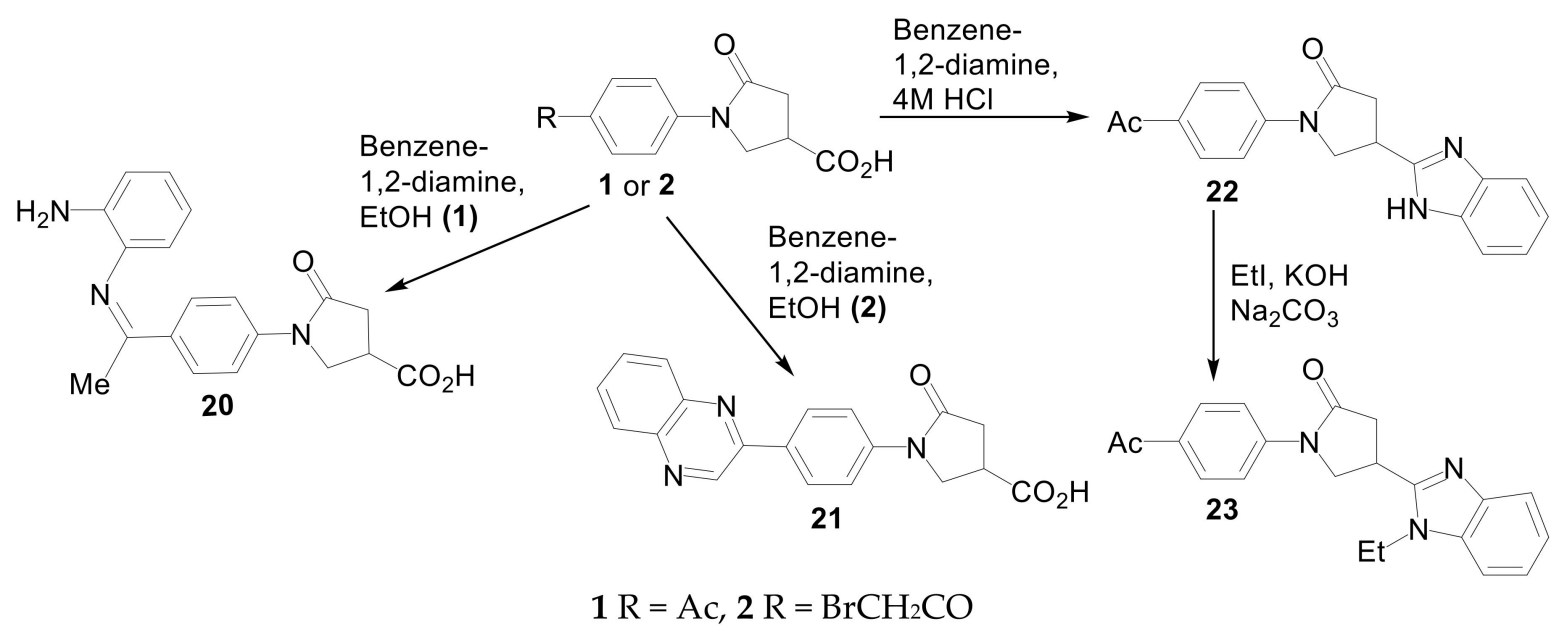
Synthesis of compounds **20–23**.

In the next stage of the work, the reactivity of acyl- **1** and *α*-bromo acyl **2** derivatives was investigated by reacting them with benzene-1,2-diamine (Scheme 3). The reactions were performed in different solvents. The reaction of the derivative **1** with diamine in ethanol yielded the condensation product 1-(4-(1-((2-aminophenyl)iminoethyl)phenyl)-5-oxopyrrolidine-3-carboxylic acid (**20**), while the bromoacyl derivative **2** under the same conditions afforded the compound **21** containing the quinoxaline moiety. The condensation in the compound **1** occurred in the acyl moiety. This resulted in a conjugated system with double bonds in the chain of the benzene ring-iminoethyl fragment-benzene ring, which gave the brightly brown-orange crystalline compound **20**. The obtained compound **20** was easily identified by the data of the NMR spectra. In the ^1^H and ^13^C-NMR spectra of compound **21**, the increased number of spectral lines in the aromatic regions of the respective spectra, the HRMS (High-resolution mass spectrometry) data, and the elemental analysis approved the formation of quinoxaline moiety. The second step of the investigation was to find out the products of the same reaction in refluxing 4 M hydrochloric acid. The reaction of compound **1**, having an acyl group on the benzene ring with benzene-1,2-diamine in refluxing 4 M hydrochloric acid, afforded the benzimidazole derivative **22** [49]. Resynthesis was carried out according to a well-known Philips method (the heating of reagents in a 4 M HCl); however, extending it to 16 h and neutralizing the mixture with aqueous ammonium hydroxide to pH 7. This resulted in the increased yield of the desired product **22** (from 40 to 92%). The condensation of brominated compound **2** with diamine under the same conditions led to the formation of the complex mixture, and product separation was unsuccessful.

For more extensive analysis of biological properties of the compounds resynthesized benzimidazole **22** [49] was alkylated with iodoethane at solvent-free conditions and in the presence of potassium hydroxide and sodium carbonate.

The reaction provided *N*-alkylated compound 1-(4-acetylphenyl)-3-(1-ethyl-1*H*-benzimidazol-2-yl)-5-oxopyrrolidine (**23**).

### 2.2. Biological Activity

All synthesized compounds **2**–**19** and **21**–**23** were tested against Gram-positive bacteria strains of *Staphylococcus aureus* (ATCC 25923), *Bacillus cereus* (ATCC 10231), *Listeria monocytogenes* (ATCC 19111) as well as Gram-negative *Pseudomonas aeruginosa* (ATCC 10145), *Escherichia coli* (ATCC 8739) and *Salmonella enterica enteritidis* (ATCC 13076) bacteria for their in vitro antibacterial activity by broth dilution and spread-plate techniques [50,51,52]. *Oxytetracycline* was used as a control (*C*) for antibacterial activity screening. The determined values of the minimum inhibition (MIC, µg/mL) and the minimum bactericidal (MBC, µg/mL) concentrations are presented in Table 2. The investigations demonstrated that the synthesized compounds possessed higher antibacterial properties than those of the known antibiotic *Oxytetracycline*.

The in vitro evaluation of the above-mentioned compounds revealed the excellent antibacterial activity of compounds **3c**, ***5b***, **15b** and **16b**. Their effect on Gram-positive bacteria strains (MIC 7.8 µg/mL and MBC 15.6 µg/mL) was 8 times higher, and against Gram-negative 16-fold higher, than those of the control *Oxytetracycline*.

It should also be noted that among the thiazoles **3a–c** the amino thiazole **3c**, containing the β-alanine moiety, displayed the best antibacterial results against all the tested bacteria strains.

Among all the tested compounds, methyl ester **4b**, hydrazone **6b** and oxadiazole derivative **17b** should be distinguished. They demonstrated four times stronger inhibition effects against Gram-positive bacteria strains and showed 8-fold higher inhibition against Gram-negative bacteria in comparison with the control. Hydrazones **6a** and (**7–12**)**b** in most cases displayed good to very good action against the tested bacteria species. However, the best activity showed hydrazone **9b** with 4-fluorophenyl moiety in the molecule.

The comparison of the antibacterial activity of both benzimidazoles showed that the alkylated derivative **22** was more potent only against the strain of *S. aureus.* In all other cases, the effect of benzimidazole **20** and its derivative **22** on test-bacteria strains was completely the same. It is also noteworthy that *S. aureus* and *E. coli* were the most sensitive to the action of benzimidazoles **20** and **22**. The minimum inhibition and minimum bactericidal concentrations of 31.25 µg/mL (MIC 15.6 µg/mL for *S. aureus* in case of **22**) were sufficient in comparison with 62.5 and 125 µg/mL of *Oxytetracycline* for the inhibition of the growth and bactericidal action of the indicated test-cultures.

The quinoxaline fragment containing derivative **21** exhibited a more marked effect than the control *Oxytetracycline* only against *B. cereus, L. monocytogenes* and *S. enterica enteritidis*. In other cases, resistance of the test-bacteria was found to be the same as that of the used control antibiotic (except for MIC in the case of *L. monocytogenes*).

Comparing the data of the biological activity of the compounds containing different substituents (amino (**a**) or phenyl (**b**)) at the 2-position of thiazole ring, it can be asserted that derivatives with 2-phenylthiazol-5-yl moiety (**3–6** and **13**)**b**, show stronger inhibition and bactericidal effects than the derivatives with 2-aminothiazol-5-yl fragment (**3–6** and **13**)**a**. The MIC of compounds with phenyl group changes in range of 7.8–62.5 µg/mL, with prevailing MIC of 15.6 µg/mL, and the MBC vary from 15.6 to 125 µg/mL, with the prevailing value of 31.25 µg/mL. Accordingly, the data of compounds with amino group are as follows: the MIC vary from 15.6 to 62.5 µg/mL, with the prevailing value of 31.25 µg/mL; and the MBC changes in range of 31.25–125 µg/mL, with the prevailing concentration of 62.5 µg/mL. It should be noted that, among the compounds of group a, the 2-aminothiazole derivative **3a** exhibited stronger activity only against *S. aureus* and *E. coli* in comparison with its analogue **3b**.

## 3. Materials and Methods

### 3.1. Synthesis

Reagents and solvents were purchased from Sigma-Aldrich (St. Louis, MO, USA) and used without further purification. The reaction course and purity of the synthesized compounds were monitored by TLC (Thin layer chromatography) using aluminum plates pre-coated with Silica gel with F254 nm (Merck KGaA, Darmstadt, Germany). Melting points were determined with a Melt-Temp Melting Point Analyzer (Electrothermal, Bibby Scientific Company, Burlington, NJ, USA) and were uncorrected. NMR spectra were recorded on a Varian Unity Inova (300, 75 MHz) and Brucker BioSpin GmbH (400, 101 and 700, 175 MHz) spectrometers. Chemical shifts were reported in (δ) ppm relative to tetramethylsilane (TMS) with the residual solvent as internal reference ([D6]DMSO, δ = 2.50 ppm for ^1^H and δ = 39.5 ppm for ^13^C). The data are reported as follows: chemical shift, multiplicity, coupling constant [Hz], integration and assignment. IR spectra (ν, cm^−1^) were recorded on a Bruker TENSOR 27 spectrometer using KBr pellets. Mass spectra were measured on a Waters (Micromas) ZQ 2000 mass spectrometer (ESI 20 eV). Elemental analyses (C, H, N) were conducted using the Elemental Analyzer CE-440 (Exeter Analytical, Inc., North Chelmsford, MA, USA); their results were found to be in good agreement (± 0.3%) with the calculated values.

*1-(4-Acetylphenyl)-5-oxopyrrolidine-3-carboxylic acid* (**1**): White solid, yield 62.9 g, 86%, m. p. 170–171 °C (2-propanol) [42]. The ^1^H and ^13^C-NMR spectra agreed with that given in the study [42].

*1-(4-(2-Bromoacetyl)phenyl)-5-oxopyrrolidine-3-carboxylic acid* (**2**): To a stirred mixture of carboxylic acid **1** (2.47 g, 10 mmol) and acetic acid (5.5 mL) a solution of bromine (9.59 g, 60 mmol) and acetic acid (15 mL) was slowly added dropwise at room temperature, and then the resulting reaction mixture further was stirred for 4 h. Afterwards, the reaction mixture was poured into the ice/water mixture (350 mL). The residual bromine was removed by adding a few milliliters of 20% sodium thiosulfate solution. The obtained precipitate was filtered off, washed with plenty of water and recrystallized from water to give the title compound **2** (white solid, yield 2.34 g, 95%, m. p. 149–150 °C (water). ^1^H-NMR (400 MHz, DMSO-d_6_): δ = 2.67–2.92 (m, 2H, COCH_2_), 3.32–3.46 (m, 1H, CH), 3.94–4.20 (m, 2H, NCH_2_), 4.76, 4.89 (2s, 2H, CH_2_Br), 7.76–8.14 (m, 4H, H_Ar_), 10.52 (br s, 1H, OH) ppm. ^13^C-NMR (101 MHz, DMSO-d_6_): δ = 33.9 (CH_2_Br), 35.0 (COCH_2_), 35.4 (CH), 49.8 (NCH_2_), 118.5, 128.6, 129.9, 143.7 (C_Ar_), 172.8 (N–C=O), 174.1 (COOH), 190.6 (C=O) ppm. IR (KBr): ν_max_ = 1738, 1691, 1660 (3C=O) cm^–1^. MS (ESI), *m*/*z*, % [M]^+^ = 326 (100), [M + 2]^+^ = 328 (98). Calcd. for C_13_H_12_BrNO_4_, %: C 47.87; H 3.71; N 4.29. Found, %: 47.78; H 3.68; N 4.29.

*1-(4-(2-Aminothiazol-5-yl)phenyl)-5-oxopyrrolidine-3-carboxylic acid* (**3a**): A mixture of carboxylic acid **2** (3.26 g, 10 mol), thiourea (1.52 g, 20 mol) and acetic acid (20 mL) was heated at 60 °C for 16 h, then the reaction mixture was diluted with water and the formed precipitate was filtered off, washed with water and hexane, and recrystallized from dimethylforamide/water (2:1) mixture to give the title compound **3 a** (white solid, yield 2.0 g, 62%, m. p. 271–272 °C (DMF/water). ^1^H-NMR (400 MHz, DMSO-d_6_): δ = 2.69–2.82 (m, 2H, COCH_2_), 3.32–3.39 (m, 1H, CH), 3.93–4.13 (m, 2H, NCH_2_), 6.96 (s, 1H, CH=C), 7.07 (s, 2H, NH_2_), 7.66 (d, *J* = 8.8 Hz, 2H, H_Ar_), 7.79 (d, *J* = 8.8 Hz, 2H, H_Ar_), 12.81 (br s, 1H, OH) ppm. ^13^C-NMR (101 MHz, DMSO-d_6_): δ = 35.2 (COCH_2_), 35.3 (CH), 50.0 (NCH_2_), 100.9 (C–S), 119.3, 125.9, 130.8, 138.2, 149.4 (CH=C, C_Ar_), 168.2 (C=N), 171.8 (C=O), 174.3 (COOH) ppm. IR (KBr): ν_max_ = 3240 (NH_2_), 1684, 1664 (2C=O). Calcd. for C_14_H_13_N_3_O_3_S, %: C 55.43; H 4.32; N 13.85. Found, %: C 55.39; H 4.29; N 13.77.

*5-Oxo-1-(4-(2-phenylthiazol-5-yl)phenyl)pyrrolidine-3-carboxylic acid* (**3b**): A mixture of compound **2** (0.4 g, 0.123 mmol) and benzencarbothioamide (0.17 g, 0.123 mmol) was refluxed in acetone (5 mL) for 6 h, then cooled down, the formed crystalline precipitate was filtered off, washed with acetone, and recrystallized from methanol to give the title compound **3b** (white solid, yield 0.19 g, 43%, m. p. 221–222 °C (methanol)). ^1^H-NMR (400 MHz, DMSO-d_6_): δ = 2.65–2.82 (m, 2H, COCH_2_), 3.28–3.41 (m, 1H, CH), 4.00–4.18 (m, 2H, NCH_2_), 7.51–7.61 (m, 3H, H_Ar_), 7.75–7.86 (m, 2H, H_Ar_), 8.01–8.18 (m, 4H, H_Ar_), 8.20 (s, 1H, CH=C), 12.82 (s, 1H, OH) ppm. ^13^C-NMR (101 MHz, DMSO-d_6_): δ = 35.1 (COCH_2_), 35.3 (CH), 49.9 (NCH_2_), 114.0 (C–S), 119.4, 126.2, 126.5, 129.3, 129.7, 130.4, 133.0, 139.0 (C_Ar_), 154.7 (CH=C), 166.9 (C=N), 172.0 (N–C=O), 174.2 (COOH) ppm. IR (KBr): ν_max_ = 3446 (OH), 1727, 1651 (2C=O). MS (ESI), *m*/*z*, %: [M + H]^+^ = 365 (100). Calcd. for C_20_H_16_N_2_O_3_S, %: C 65.92; H 4.43; N 7.69. Found, %: C 65.82; H 4.35; N 7.62.

*1-(4-(2-((2-Carboxyethyl)-4-methylanilino)-1,3-thiazol-5-yl)phenyl)-5-oxopyrrolidine-3-carboxylic acid* (**3c**): To a solution of 3-(1-(p-tolyl)thioureido)propanoic acid (0.24 g, 1 mmol) in acetone (5 mL), compound **2** (0.42 g, 1.3 mmol) was added and the mixture was heated at reflux for 8 h. After the completion of the reaction, the mixture was cooled down, the formed crystalline solid was filtered off, washed with acetone, and dissolved in 10% aqueous sodium acetate solution (25 mL) by boiling, and then cooled down. The formed precipitate was filtered off, washed with water, and recrystallized from methanol to give the title compound **3 c** (light yellow solid, yield 0.38 g, 82%, m. p. 196–197 °C (methanol)). ^1^H-NMR (400 MHz, DMSO-d_6_): δ = 2.32 (s, 3H, CH_3_), 2.40 (t, *J* = 7.9 Hz, 2H, CH_2_COO), 2.51–2.98 (m, 3H, COCH_2_ + CH), 3.88–4.05 (m, 2H, NCH_2_), 4.10 (t, *J* = 7.9 Hz, 2H, NCH_2_CH_2_), 6.99 (s, 1H, CH=C), 7.26 (d, *J* = 8.4 Hz, 2H, H_Ar2_^,^_, 6_^,^), 7.32 (d, *J* = 8.3 Hz, 2H, H_Ar3_^,^_, 5_^,^), 7.67 (d, *J* = 8.8 Hz, 2H, H_Ar2, 6_), 7.82 (d, *J* = 8.7 Hz, 2H, H_Ar3, 5_) ppm. ^13^C-NMR (101 MHz, DMSO-d_6_): δ = 20.7 (CH_3_), 35.0 (COCH_2_), 37.1 (CH), 37.5 (CH_2_), 50.2 (NCH_2_), 51.9 (CH_2_), 101.2 (C–S), 118.9, 125.9, 126.7, 130.1, 130.4, 136.5, 139.0, 142.2, 150.0 (CH=C, C_Ar_), 169.1 (C=N), 173.7 (C=O), 174.5 (COOH), 176.0 (COOH) ppm. IR (KBr): ν_max_ = 1683, 1605, 1575 (3C=O). MS (ESI), *m*/*z*, %: [M + H]^+^ = 466 (100). Calcd. for C_24_H_23_N_3_O_5_S, %: C 61.92; H 4.98; N 9.03. Found, %: C 61.68; H 4.62; N 8.91.

#### 3.1.1. General Procedure for the Preparation of Esters 4a,b

To a solution of the corresponding carboxylic acid **3** (2.75 mmol) in methanol, concentrated sulfuric acid (1 mL) was added dropwise and the mixture was heated at reflux for 5 h. Then the solvent was evaporated under reduced pressure, and the residue neutralized with 7% (**a**) or 10% (**b**) sodium carbonate solution to pH 7. The obtained solid was filtered off, washed with plenty of water, and recrystallized from methanol to give the title compound **4a** (light yellow solid, yield 0.54 g, 62%, m. p. 271–272 °C (methanol)) and **4b** (light yellow solid, yield 0.71 g, 71%, m. p. 139–140 °C (methanol)).

*Methyl 1-(4-(2-aminothiazol-5-yl)phenyl)-5-oxopyrrolidine-3-carboxylate* (**4a**): ^1^H-NMR (400 MHz, DMSO-d_6_): δ = 2.64–2.89 (m, 2H, COCH_2_), 3.43–3.52 (m, 1H, CH), 3.68 (s, 3H, OCH_3_), 3.94–4.15 (m, 2H, NCH_2_), 6.95 (s, 1H, CH=C), 7.05 (s, 2H, NH_2_), 7.65 (d, J = 8.8 Hz, 2H, H_Ar_), 7.79 (d, J = 8.8 Hz, 2H, H_Ar_) ppm. ^13^C-NMR (101 MHz, DMSO-d_6_): δ = 34.9 (COCH_2_), 35.1 (CH), 49.7 (NCH_2_), 52.2 (OCH_3_), 100.9 (C–S), 119.2, 125.8, 130.8, 138.0, 149.3 (CH=C, C_Ar_), 168.2 (C=N), 171.5 (C=O), 173.1 (COO) ppm. IR (KBr): ν_max_ = 3430 (NH_2_), 1692, 1639 (2C=O). MS (ESI), *m*/*z*, %: [M + H]^+^ = 318 (100). Calcd. for C_15_H_15_N_3_O_3_S, %: C 56.77; H 4.76; N 13.24. Found, %: C 56.66; H 4.75; N 13.40.

*Methyl 5-oxo-1-(4-(2-phenylthiazol-5-yl)phenyl)pyrrolidine-3-carboxylate* (**4b**): ^1^H-NMR (400 MHz, DMSO-d_6_): δ = 2.71–2.80 (m, 2H, COCH_2_), 3.45–3.55 (m, 1H, CH), 3.70 (s, 3H, OCH_3_), 3.99–4.18 (m, 2H, NCH_2_), 7.44–7.62 (m, 3H, H_Ar_), 7.77 (d, J = 8.8 Hz, 2H, H_Ar_), 7.96–8.12 (m, 4H, A_Ar_), 8.13 (s, 1H, CH=C) ppm. ^13^C-NMR (101 MHz, DMSO-d_6_): δ = 34.9 (COCH_2_), 35.1 (CH), 49.7 (NCH_2_), 52.2 (OCH_3_), 114.0 (C–S), 119.4, 126.2, 126.5, 129.3, 130.4, 133.0, 138.9, (C_Ar_), 154.7 (CH=C), 166.9 (C=N), 171.7 (N–C=O), 173.1 (COO) ppm. IR (KBr): ν_max_ = 1738, 1699 (2C=O). MS (ESI), *m*/*z*, %: [M + H]^+^ = 379 (100). Calcd. for C_21_H_18_N_2_O_3_S, %: C 66.65; H 4.79; N 7.40. Found, %: C 66.54; H 4.82; N 7.32.

#### 3.1.2. General Procedure for the Preparation of Hydrazides 5a,b

A mixture of the corresponding methyl ester **4** (10 mmol), hydrazine monohydrate (2.5 g, 2.45 mL, 50 mmol) and propan-2-ol (30 mL) was heated at reflux for 10 h. After completion of the reaction (TLC), the mixture was cooled to room temperature, the formed precipitate filtered off, washed with water, and recrystallized from methanol to give the title compound **5a** (white solid, yield 2.7 g, 81.4%, m. p. 248–248 °C) and **5b** (white solid, yield 2.46 g, 65%, m. p. 231–232 °C).

*1-(4-(2-Aminothiazol-5-yl)phenyl)-5-oxopyrrolidine-3-carbohydrazide* (**5a**): ^1^H-NMR (400 MHz, DMSO-d_6_): δ = 2.53–2.81 (m, 2H, COCH_2_), 3.10–3.23 (m, 1H, CH), 3.88–4.08 (m, 2H, NCH_2_), 4.30 (s, 2H, NHNH_2_), 6.94 (s, 1H, CH=C), 7.04 (s, 2H, NH_2_), 7.65 (d, J = 8.4 Hz, 2H, H_Ar_), 7.78 (d, J = 8.4 Hz, 2H, H_Ar_), 9.28 (s, 1H, NH) ppm. ^13^C-NMR (101 MHz, DMSO-d_6_): δ = 34.1 (COCH_2_), 35.8 (CH), 50.7 (NCH_2_), 100.8 (C–S), 119.1, 125.8, 130.7, 138.2, 149.4 (CH=C, C_Ar_), 168.2 (C=N), 171.6, 172.1 (2C=O) ppm. IR (KBr): ν_max_ = 3391, 3316 (NH, NH_2_), 1674, 1643 (2C=O). MS (ESI), *m*/*z*, %: [M + H]^+^ = 317 (100).

*5-Oxo-1-(4-(2-phenylthiazol-5-yl)phenyl)pyrrolidine-3-carbohydrazide* (**5b**): ^1^H-NMR (400 MHz, DMSO-d_6_): δ = 2.62–2.80 (m, 2H, COCH_2_), 3.12–3.28 (m, 1H, CH), 3.86–4.08 (m, 2H, NCH_2_), 4.31 (s, 2H, NHNH_2_), 7.48–7.56 (m, 3H, H_Ar_), 7.77 (d, J = 8.8 Hz, 2H, H_Ar_), 7.98–8.09 (m, 4H, H_Ar_), 8.12 (s, 1H, CH=C), 9.31 (s, 1H, NH) ppm. ^13^C-NMR (101 MHz, DMSO-d_6_): δ = 34.0 (COCH_2_), 35.8 (CH), 50.7 (NCH_2_), 113.9 (C–S), 119.3, 126.2, 126.5, 129.3, 129.6, 130.4, 133.0, 139.1 (C_Ar_), 154.8 (CH=C), 166.9 (C=N), 171.5, 172.2 (2C=O) ppm. IR (KBr): ν_max_ = 3318, 3281 (NH, NH_2_), 1682, 1638 (2C=O). MS (ESI), *m*/*z*, %: [M + H]^+^ = 379 (100). Calcd. for C_20_H_18_N_4_O_2_S, %: C 63.47; H 4.79; N 14.80. Found, %: C 63.37; H 4.72; N 14.71.

#### 3.1.3. General Procedure for the Preparation of Hydrazones 6–12

A mixture of the corresponding hydrazide **5a**,**b** (10 mmol), benzencarbaldehyde (11 mmol) or thiophene-2-carboxaldehyde (2.24 g, 20 mmol) and dimethylformamide (30 mL) was refluxed for 1–3 h. Afterwards, the reaction mixture was cooled down, diluted with water (50 mL), the obtained product was filtered off, washed with water and ether, and recrystallized from the indicated solvent.

*1-(4-(2-Aminothiazol-5-yl)phenyl)-3-(benzylidenehydrazinocarbonyl)-5-oxopyrrolidine* (**6a**): light yellow solid, yield 3.65 g, 90.2%, m. p. 239–240 °C (methanol). ^1^H-NMR (400 MHz, DMSO-d_6_): δ = *Z*/*E* 2.68–2.94 (m, 2H, COCH_2_), 3.36–3.41 (m, 0.35H, CH), 3.93–4.25 (m, 0.65H, CH + 2H, NCH_2_), 6.94, 6.95 (2s, 1H, CH=C), 7.05 (s, 2H, NH_2_), 7.24–7.90 (m, 9H, H_Ar_), 8.05 (s, 0.65H, N=CH), 8.23 (s, 0.35H, N=CH), 11.58 (s, 0.65H, NH), 11.65 (s, 0.35H, NH) ppm. IR (KBr): ν_max_ = 3398 (NH_2_), 1681, 1668 (2C=O). MS (ESI), *m*/*z*, %: [M + H]^+^ = 406 (100). Calcd. for C_21_H_19_N5_4_O_2_S, %: C 62.21; H 4.72; N 17.27. Found, %: C 62.16; H 4.69; N 17.23.

*3-(Benzylidenehydrazinocarbonyl)-1-(4-(2-phenylthiazol-5-yl)phenyl)-5-oxopyrrolidine* (**6b**): light yellow solid, yield 4.05 g, 92%, m. p. 214–215 °C (methanol). ^1^H-NMR (400 MHz, DMSO-d_6_): δ = *Z*/*E* 2.73–2.95 (m, 2H, COCH_2_); 3.35–3.47 (m, 0.35H, CH); 3.95–4.31 (m, 0.65H, CH + 2H, NCH_2_); 7.38–7.61 (m, 6H, H_Ar_), 7.66–7.78 (m, 2H, H_Ar_), 7.81 (d, J = 8.6 Hz, 2H, H_Ar_), 7.96–8.28 (m, 6H, H_Ar_ + CH=C + N=CH), 11.61 (s, 0.65H, NH), 11.68 (s, 0.35H, NH) ppm. IR (KBr): ν_max_ = 3178 (NH), 1700, 1668 (2C=O). MS (ESI), *m*/*z*, %: [M + H]^+^ = 447 (100). Calcd. for C_27_H_22_N_4_O_2_S, %: C 69.51; H 4.75; N 12.01. Found, %: C 69.61; H 4.86; N 11.94.

*1-(4-(2-Aminothiazol-5-yl)phenyl)-3-(4-chlorobenzylidenehydrazinocarbonyl)-5-oxopyrrolidine* (**7a**): light yellow solid, yield 3.93 g, 89.5%, m. p. 254 °C (methanol). ^1^H-NMR (400 MHz, DMSO-d_6_): δ = Z/E 2.69–2.89 (m, 2H, COCH_2_), 3.34–3.38 (m, 0.4H, CH), 3.94–4.22 (m, 0.6H, CH + 2H, NCH_2_), 6.95 (s, 1H, CH=C), 7.04 (s, 2H, NH_2_), 7.48–7.82 (m, 8H, H_Ar_), 8.03 (s, 0.65H, N=CH), 8.22 (s, 0.35H, N=CH), 11.64 (s, 0.65H, NH), 11.71 (s, 0.35H, NH) ppm. IR (KBr): ν_max_ = 3252 (NH_2_), 1673, 1656 (2C=O). MS (ESI), *m*/*z*, %: [M + H]^+^ = 440 (100). Calcd. for C_21_H_18_ClN_5_O_2_S, %: C 57.33; H 4.12; N 15.92. Found, %: C 57.27; H 4.08; N 15.88.

*3-(3-Chlorobenzylidenehydrazinocarbonyl)-1-(4-(2-phenylthiazol-5-yl)phenyl)-5-oxopyrrolidine* (**7b**): yellow solid, yield 3.45 g, 69%, m. p. 141–142 °C (methanol). ^1^H-NMR (400 MHz, DMSO-d_6_): δ = *Z*/*E* 2.72–2.95 (m, 2H, COCH_2_), 3.34–3.45 (m, 0.4H, CH), 3.99–4.25 (m, 0.6H, CH + 2H, NCH_2_), 7.33–8.28 (m, 15H, H_Ar_ + CH=C + N=CH), 11.70 (s, 0.65H, NH), 11.80 (s, 0.35H, NH) ppm. IR (KBr): ν_max_ = 3265 (NH), 1668, 1627 (2C=O). Calcd. for C_27_H_21_ClN_4_O_2_S, %: C 64.73; H 4.22; N 11.18. Found, %: C 64.67; H 4.19; N 11.29.

*1-(4-(2-Aminothiazol-5-yl)phenyl)-3-(4-nitrobenzylidenehydrazinocarbonyl)-5-oxopyrrolidine* (**8a**): light yellow solid, yield 2.79 g, 62%, m. p. 234–236 °C (ethanol). ^1^H-NMR (400 MHz, DMSO-d_6_): δ = *Z*/*E* 2.69–2.96 (m, 2H, COCH_2_), 3.35–3.44 (m, 0.35H, CH), 3.91–4.28 (m, 0.6H, CH + 2H, NCH_2_), 6.95 (s, 1H, CH=C), 7.04 (s, 2H, NH_2_), 7.50–8.41 (m, 9H, H_Ar_ + N=CH), 11.87 (s, 0.65H, NH), 11.94 (s, 0.35H, NH) ppm. IR (KBr): ν_max_ = 3435 (NH_2_), 3245 (NH), 1681, 1656 (2C=O). MS (ESI), *m*/*z*, %: [M + H]^+^ = 451 (100). Calcd. for C_21_H_18_N_6_O_4_S, %: C 55.99; H 4.03; N 18.66. Found, %: C 55.96; H 4.01; N 18.55.

*3-(4-Nitrobenzylidenehydrazinocarbonyl)-1-(4-(2-phenylthiazol-5-yl)phenyl)-5-oxopyrrolidine* (**8b**): yellow solid, yield 4.55 g, 89%, m. p. 150–151 °C (methanol). ^1^H-NMR (400 MHz, DMSO-d_6_): δ = *Z*/*E* 2.77–2.96 (m, 2H, COCH_2_), 3.36–3.48 (m, 0.4H, CH), 4.01–4.27 (m, 0.6H, CH + 2H, NCH_2_), 7.45–8.38 (m, 15H, H_Ar_ + CH=C + N=CH), 11.90 (s, 0.65H, NH), 11.97 (s, 0.35H, NH) ppm. IR (KBr): ν_max_ = 3221 (NH), 1674, 1612 (2C=O). Calcd. for C_27_H_21_N_5_O_4_S, %: C 63.39; H 4.14; N 13.69. Found, %: C 63.42; H 4.15; N 13.29.

*3-(4-Fluorobenzylidenehydrazinocarbonyl)-1-(4-(2-phenylthiazol-5-yl)phenyl)-5-oxopyrrolidine* (**9b**): light yellow solid, yield 3.97 g, 82%, m. p. 216–217 °C (methanol). ^1^H-NMR (400 MHz, DMSO-d_6_): δ = *Z*/*E* 2.77–2.91 (m, 2H, COCH_2_), 3.35–3.41 (m, 0.4H, CH), 4.01–4.24 (m, 0.6H, CH + 2H, NCH_2_), 7.22–8.26 (m, 15H, H_Ar_ + CH=C + N=CH), 11.61 (s, 0.65H, NH), 11.68 (s, 0.35H, NH) ppm. IR (KBr): ν_max_ = 3265 (NH), 1668, 1604 (2C=O). Calcd. for C_27_H_21_FN_4_O_2_S, %: C 66.93; H 4.37; N 11.56. Found, %: C 66.72; H 4.25; N 11.49.

*5-Oxo-1-(4-(2-phenylthiazol-5-yl)phenyl)-N’-(thiophen-2-ylmethylene)pyrrolidine-3-*carbohydrazide (**10b**): light yellow solid, yield 3.54 g, 75%, m. p. 198–200 °C (methanol). ^1^H-NMR (400 MHz, DMSO-d_6_): δ = *Z*/*E* 2.72–2.90 (m, 2H, COCH_2_), 3.34–3.40 (m, 0.4H, CH), 3.82–4.30 (m, 0.6H, CH + 2H, NCH_2_), 7.10–8.18 (m, 13H, H_Ar_ + CH=C), 8.23 (s, 0.6H, N=CH), 8.45 (s, 0.4H, N=CH), 11.59 (s, 0.6H, NH), 11.62 (s, 0.4H, NH) ppm. IR (KBr): ν_max_ = 3265 (NH), 1673, 1610 (2C=O). Calcd. for C_25_H_20_N_4_O_2_S_2_, %: C 66.54; H 4.27; N 11.86. Found, %: C 66.50; H 4.24; N 11.89.

#### 3.1.4. General Procedure for the Preparation of Hydrazones 11b, 12b

A mixture of hydrazide **5b** (1 g, 2.64 mmol) and dimethylformamide (**11b**) or 1,4-dioxane (**12b**) (10 mL) was heated to its boiling point and 3- or 4-pyridinecarbaldehyde (5.28 mmol) was added dropwise. The mixture was refluxed for 1 h, diluted with water (20 mL) (**11b**) or the solvent was evaporated under reduced pressure, the resin mass was poured with diethyl ether (10 mL), stirred and left in refrigerator (**12b**). The obtained precipitate was filtered off, washed with water and ether, and recrystallized 2-propanol to give the title compound **11b** (yellow solid, yield 1.22 g, 99%, m. p. 203–204 °C (propan-2-ol) or **12b** (yellow solid, yield 1.19 g, 96.5%, m. p. 236–237 °C (propan-2-ol).

*5-Oxo-1-(4-(2-phenylthiazol-5-yl)phenyl)-N’-(pyridin-3-ylmethylene)pyrrolidine-3-carbohydrazide* (**11b**): ^1^H-NMR (400 MHz, DMSO-d_6_): δ = *Z*/*E* 2.75–2.94 (m, 2H, COCH_2_), 3.34–3.46 (m, 0.35H, CH), 3.94–4.34 (m, 0.65H, CH + 2H, NCH_2_), 7.43–8.92 (m, 15H, H_Ar_ + CH=C + N=CH), 11.76 (s, 0.65H, NH), 11.85 (s, 0.35H, NH) ppm. IR (KBr): ν_max_ = 3255 (NH), 1674, 1609 (2C=O). Calcd. for C_26_H_21_N_5_O_2_S, %: C 66.79; H 4.53; N 14.98. Found, %: C 66.76; H 4.48; N 14.89.

*5-Oxo-1-(4-(2-phenylthiazol-5-yl)phenyl)-N’-(pyridin-4-ylmethylene)pyrrolidine-3-carbohydrazide* (**12b**): ^1^H-NMR (400 MHz, DMSO-d_6_): δ = *Z*/*E* 2.71–3.02 (m, 2H, COCH_2_), 3.32–3.53 (m, 0.4H, CH), 4.00–4.27 (m, 0.6H, CH + 2H, NCH_2_), 7.47–8.27 (m, 13H, H_Ar_ + CH=C + N=CH), 8.57–8.79 (m, 2H, H_Pyr_), 11.91 (s, 0.6H, NH), 11.97 (s, 0.4H, NH) ppm. IR (KBr): ν_max_ = 3213 (NH), 1684, 1600 (2C=O). Calcd. for C_26_H_21_N_5_O_2_S, %: C 66.79; H 4.53; N 14.98. Found, %: C 62.68; H 4.48; N 14.76.

#### 3.1.5. General Procedure for the Preparation of Pyrroles 13a,b

A mixture of the corresponding acid hydrazide **5a**,**b** (10 mmol), hexane-2,5-dione (1.71 g, 1.8 mL, 15 mmol) glacial acetic acid (1.5 mL) and ethanol (30 mL) was heated at reflux for 10 h. Then the solvent was evaporated under reduced pressure, and the residue was poured with water (50 mL). The formed precipitate was filtered off, washed with water and ether, and recrystallized from the indicated solvent.

*1-(4-(2-Aminothiazol-5-yl)phenyl)-N-(2,5-dimethyl-1H-pyrrol-1-yl)-5-oxopyrrolidine-3-carboxamide* (**13a**): light yellow solid, yield 2.25 g, 57%, m. p. 257–258 °C (2-propanol). ^1^H-NMR (400 MHz, DMSO-d_6_): δ = 2.00 (s, 6H, 2CH_3_), 2.70–2.94 (m, 2H, COCH_2_), 3.43–3.52 (m, 1H, CH), 3.96–4.20 (m, 2H, NCH_2_), 5.65 (s, 2H, 2CH), 6.96 (s, 1H, CH=C), 7.05 (s, 2H, NH_2_), 7.68 (d, J = 8.8 Hz, 2H, H_Ar_), 7.80 d, J = 8.8 Hz, 2H, H_Ar_), 10.91 (s, 1H, NH) ppm. ^13^C-NMR (101 MHz, DMSO-d_6_): δ = 10.9, 11.0 (2CH_3_), 34.1 (COCH_2_), 35.7 (CH), 50.3 (NCH_2_), 100.9, 103.1, 119.3, 125.8, 126.7, 130.8, 138.1, 149.4 (CH=C, C_Ar_, C_Pyr_), 168.2 (C=N), 171.6, 171.9 (2C=O) ppm. IR (KBr): ν_max_ = 3403 (NH_2_), 3325 (NH), 1693, 1678 (2C=O). MS (ESI), *m*/*z*, %: [M + H]^+^ = 396 (100). Calcd. for C_20_H_21_N_5_O_2_S, %: C 60.74; H 5.35; N 17.71. Found, %: C 60.67; H 5.31; N 17.69.

*N-(2,5-Dimethyl-1H-pyrrol-1-yl)-5-oxo-1-(4-(2-phenylthiazol-5-yl)phenyl)pyrrolidine-3-carboxamide* (**13b**): light brown solid, yield 4.2 g, 92%, m. p. 119–120 °C (methanol). ^1^H-NMR (400 MHz, DMSO-d_6_): δ = 2.01, 2.02 (2s, 6H, 2CH_3_), 2.73–2.99 (m, 2H, COCH_2_), 3.47–3.55 (m, 1H, CH), 4.02–4.24 (m, 2H, NCH_2_), 5.65 (s, 2H, 2CH), 7.50–7.57 (m, 3H, H_Ar_), 7.81 (d, *J* = 8.8 Hz, 2H, H_Ar_), 8.02–8.09 (m, 4H, H_Ar_), 8.14 (s, 1H, CH=C), 10.93 (s, 1H, NH) ppm. ^13^C-NMR (101 MHz, DMSO-d_6_): δ = 11.0 (2CH_3_), 34.1 (COCH_2_), 35.8 (CH), 50.3 (NCH_2_), 103.1, 114.0, 119.5, 126.2, 126.5, 126.7, 129.3, 129.8, 130.4 133.0, 139.0 (C_Ar_, C_Pyr_), 154.7 (CH=C), 167.0 (C=N), 171.7, 171.8 (2C=O) ppm. IR (KBr): ν_max_ = 3317 (NH), 1699, 1609 (2C=O). MS (ESI), *m*/*z*, %: [M + H]^+^ = 457 (100). Calcd. for C_26_H_24_N_4_O_2_S, %: C 68.40; H 5.30; N 12.17. Found, %: C 68.29; H 5.23; N 12.03.

*5-Oxo-1-(5-oxo-1-(4-(2-phenylthiazol-5-yl)phenyl)pyrrolidine-3-carboxamido)pyrrolidine-3-carboxylic acid* (**14b**): A mixture of acid hydrazide **5b** (1 g, 2.64 mmol), itaconic acid (1.2 g, 9.23 mmol) and toluene (30 mL) was heated at reflux for 11 h, then cooled down, the obtained crystalline solid was filtered off, washed with water, and recrystallized from methanol to give the title compound **14b** (light yellow solid, yield 0.70 g, 54%, m. p. 170–171 °C (methanol)). ^1^H-NMR (400 MHz, DMSO-d_6_): δ = 2.51–2.88 (m, 4H, 2COCH_2_), 3.25–3.42 (m, 2H, 2CH), 3.55–4.16 (m, 4H, 2NCH_2_), 7.50–7.56 (m, 3H, H_Ar_), 7.75–7.80 (m, 2H, H_Ar_), 8.03–8.11 (m, 4H, H_Ar_), 8.13 (s, 1H, CH=C), 10.40 (s, 1H, NH), 12.84 (br s, 1H, OH) ppm. ^13^C-NMR (101 MHz, DMSO-d_6_): δ = 31.3, 33.6 (2COCH_2_), 34.2, 35.5 (2CH), 49.7, 50.3 (2NCH_2_), 114.0, 119.4, 126.2, 126.5, 129.3, 129.7, 130.4, 133.0, 139.0 (C_Ar_), 154.7 (CH=C), 167.0 (C=N), 170.9, 171.4, 171.8, 174.1 (4C=O) ppm. IR (KBr): ν_max_ = 3421 OH), 3274 (NH), 1726, 1717, 1699, 1682 (4C=O). Calcd. for C_25_H_22_N_4_O_5_S, %: C 61.21; H 4.52; N 11.42. Found, %: 61.28; H 4.62; N 11.50.

*2-(5-Oxo-1-(4-(2-phenylthiazol-5-yl)phenyl)pyrrolidine-3-carbonyl)-N-phenylhydrazine-1-carbothioamide* (**15b**): To a solution of hydrazide **5b** (1 g, 2.64 mmol) in methanol (10 mL), phenyl isothiocyanate (0.714 g, 0.63 mL, 5.28 mmol) was added dropwise and the mixture was heated at reflux for 3 h. After completion of the reaction the mixture was cooled down, the obtained crystalline solid filtered off, washed with methanol and recrystallized from methanol to give the title compound **15b** (white solid, yield 1.17 g, 86.5% m. p. 209–210 °C (methanol)). ^1^H-NMR (400 MHz, DMSO-d_6_): δ = 2.70–2.93 (m, 2H, COCH_2_), 3.31–3.42 (m, 1H, CH), 3.96–4.22 (m, 2H, NCH_2_), 7.08–7.22 (m, 1H, H_Ar_), 7.31–7.59 (m, 8H, H_Ar_), 7.69–7.83 (m, 2H, H_Ar_), 7.92–8.10 (m, 3H, H_Ar_), 8.13 (s, 1H, CH=C), 9.58 (s, 1H, NH), 9.68, 9.88 (2br s, 1H, NH), 10.12, 10.22 (2s, 1H, NH) ppm. ^13^C-NMR (101 MHz, DMSO-d_6_): δ = 34.0 (COCH_2_), 35.6 (CH), 50.3 (NCH_2_), 114.0 (C–S), 119.4, 126.2, 126.5, 128.1, 128.2, 129.3, 129.7, 130.4, 133.0, 139.1, 139.2 (C_Ar_), 154.7 (CH=C), 166.9 (C=N), 167.8, 168.5, 172.1 (C=S, 2C=O) ppm. IR (KBr): ν_max_ = 3163–3059 (NH), 1715, 1681 (2C=O). Calcd. for C_27_H_23_N_5_O_2_S_2_, %: C 63.14; H 4.51; N 13.63. Found, %: C 63.26; H 4.57; N 13.81.

*3-(4-Phenyl-5-thioxo-4,5-dihydro-1H-1,2,4-triazol-3-yl)-1-(4-(2-phenylthiazol-5-yl)phenyl)-5-oxopyrrolidine* (**16b**): A mixture of thiosemicarbazide **15b** (1 g, 1.94 mmol) and 4% sodium hydroxide solution was refluxed for 6 h. Then, the reaction mixture was cooled down, acidified with diluted hydrochloric acid to pH 1, refluxed for 15 min, afterwards cooled down and neutralized with 10% sodium carbonate solution to pH 7. The formed solid was filtered off, washed with water, and recrystallized from methanol to give the title compound **16b** (yellow solid, yield 0.86 g, 89.5%, m. p. 191–192 °C (methanol)). ^1^H-NMR (400 MHz, DMSO-d_6_): δ = 2.55–2.86 (m, 2H, COCH_2_), 3.42–3.76 (m, 1H, CH), 3.87–4.14 (m, 2H, NCH_2_), 7.25–8.11 (m, 14H, H_Ar_), 8.13 (s, 1H, CH=C), 13.89 (s, 1H, NH) ppm. ^13^C-NMR (101 MHz, DMSO-d_6_): δ = 27.8 (COCH_2_), 36.2 (CH), 50.5 (NCH_2_), 114.0, 119.6, 126.2, 126.4, 128.6, 129.3, 129.6, 129.8, 130.4, 133.0, 133.5, 138.8, 152.9 (C_Ar_), 154.7 (CH=C), 166.9, 168.4 (C=S, C=N), 171.3 C=O) ppm. IR (KBr): ν_max_ = 3406 (NH), 1685 (C=O). Calcd. for C_27_H_21_N_5_OS_2_, %: C 65.43; H 4.27; N 14.13. Found, %: C 65.39; H 4.14; N 14.11.

*3-(5-Thioxo-4,5-dihydro-1,3,4-oxadiazol-2-yl)-1-(4-(2-phenylthiazol-5-yl)phenyl)-5-oxopyrrolidine* (**17b**): To a solution of potassium hydroxide (0.56 g, 10 mmol) in methanol (25 mL), carbon disulfide (0.76 g, 10 mmol) was added dropwise. The obtained mixture was stirred at room temperature for 15 min, and then methanolic solution of acid hydrazide **5b** (1.89 g, 5 mmol/50 mL) poured over. The reaction mixture was refluxed for 15 h. After completion of the reaction (TLC), the solvent was evaporated under reduced pressure, the residue was poured with water (30 mL) and the mixture was acidified with diluted hydrochloric acid (1:1) to pH 1. The crystalline solid was filtered off, washed with plenty of water and recrystallized from methanol to give the title compound **17b** (white solid, yield 3.21 g, 76.5%, m. p. 254–255 °C (methanol)). H-NMR (400 MHz, DMSO-d_6_): δ = 2.82–3.07 (m, 2H, COCH_2_), 3.94–4.04 (m, 1H, CH), 4.10–4.35 (m, 2H, NCH_2_), 7.49–7.58 (m, 3H, H_Ar_), 7.78 (d, J = 8.8 Hz, 2H, H_Ar_), 7.99–8.11 (m, 4H, H_Ar_), 8.15 (s, 1H, CH=C), 14.42 (br s, 1H, NH) ppm. ^13^C-NMR (101 MHz, DMSO-d_6_): δ = 27.9 (COCH_2_), 35.2 (CH), 49.8 (NCH_2_), 114.1, 119.6, 126.2, 126.5, 129.3, 129.9, 130.4, 133.0, 138.8, 154.7, 163.9 (CH=C, C_Ar_), 167.0 (C=N), 171.1, 178.0 (C=O, C=S) ppm. IR (KBr): ν_max_ = 3110 (NH), 1658 (C=O). Calcd. for C_21_H_16_N_4_O_2_S_2_, %: C 59.98; H 3.84; N 13.32. Found, %: C 59.85; H 3.80; N 13.25.

*3-(4-Amino-5-thioxo-4,5-dihydro-1H-1,2,4-triazol-3-yl)-1-(4-(2-phenylthiazol-5-yl)phenyl)-5-oxopyrrolidine* (**18b**): A mixture of hydrazide **5b** (0.7 g, 1.85 mmol), potassium hydroxide (0.01 g, 0.19 mmol), carbon disulfide (0.28 g, 3.7 mmol) and methanol (35 mL) was refluxed for 15 h. Then, the solvent was removed under reduced pressure, and the residue was poured with diethyl ether (15 mL). The formed crystalline solid was filtered off, washed with diethyl ether and dried. Afterwards, the solid was poured with water, hydrazine monohydrate (0.28 g, 5.6 mmol) was added and the mixture was refluxed for 10 h. After completion of the reaction, the mixture was cooled down, diluted with water (20 mL) and neutralized with 3 M hydrochloric acid to pH 7. The formed solid was filtered off, washed with water, and recrystallized from methanol to give the title compound **18b** (light orange solid, yield 0.54 g, 67%, m. p. 245–246 °C (methanol)). ^1^H-NMR (400 MHz, DMSO-d_6_): δ = 2.62–3.08 (m, 2H, COCH_2_), 3.12–3.29 (m, 0.35H, CH), 3.85–4.34 (m, 0.65H, CH + 2H, NCH_2_), 5.59 (s, 2H, NH_2_), 7.40–8.10 (m, 9H, H_Ar_), 8.14 (s, 1H, CH=C), 13.63 (s, 1H, NH) ppm. ^13^C-NMR (101 MHz, DMSO-d_6_): δ = 27.3 (COCH_2_), 35.4 (CH), 50.4 (NCH_2_), 114.1 (C–S), 119.6, 126.2, 126.5, 129.3, 129.8, 130.4, 133.0, 139.1, 152.6, 154.7, 158.4 (C_Ar_), 166.9, 167.2, 172.2 (C=S, N–C=N, C=O) ppm. Calcd. for C_21_H_18_N_6_OS_2_, %: C 58.04; H 4.18; N 19.34. Found, %: C 57.95; H 4.05; N 19.27.

*3-(4-(Benzylideneamino)-5-thioxo-4,5-dihydro-1H-1,2,4-triazol-3-yl)-1-(4-(2-phenylthiazol-5-yl)phenyl)-5-oxopyrrolidine* (**19b**): To a mixture of compound **18b** (1 g, 2.3 mmol), benzenecarbaldehyde (0.49 g; 0.47 mL, 4.6 mmol) and ethanol (50 mL) catalytic amount of concentrated hydrochloric acid was added (0.5 mL) and the mixture was refluxed for 5 h, then cooled down. The formed crystalline solid was filtered off, washed with water, and recrystallized from methanol to give the title compound **19b** (light yellow solid, yield 1.06 g, 88%, m. p. 189–190 °C (methanol)). ^1^H-NMR (400 MHz, DMSO-d_6_): δ = 2.90–3.08 (m, 2H, COCH_2_), 4.03–4.13 (m, 1H, CH), 4.16–4.34 (m, 2H, NCH_2_), 7.40–7.70 (m, 6H, H_Ar_), 7.78 (d, *J* = 8.7 Hz, 2H, H_Ar_), 7.83–8.09 (m, 6H, H_Ar_), 8.13 (s, 1H, CH=C), 10.14 (s, 1H, N=CH), 13.97 (s, 1H, NH) ppm. ^13^C-NMR (101 MHz, DMSO-d_6_): δ = 27.3 (COCH_2_), 35.5 (CH), 50.3 (NCH_2_), 114.0, 119.6, 126.2, 126.4, 128.6, 129.2, 129.3, 132.1, 133.0, 138.9, 151.5 (CH=N, C_Th_, C_Ar_), 154.7 (CH=C), 162.0 (C=NNH), 162.9 (C=S), 166.9 (C=N), 171.7 (C=O) ppm. IR (KBr): ν_max_ = 3278 (NH), 1681 (C=O), 1482 (C=N), 1134 (C=S). Calcd. for C_28_H_22_N_6_OS_2_, %: C 64.35; H 4.24; N 16.08. Found, %: C 64.24; H 4.22; N 15.96.

*1-(4-(1-((2-aminophenyl)iminoethyl)phenyl)-5-oxopyrrolidine-3-carboxylic acid* (**20**): A mixture of acid **1** (2.47 g, 0.01 mol) and benzene-1,2-diamine (2.16 g, 0.02 mol) in ethanol (25 ml) was refluxed for 20 h, then cooled down, the formed crystalline solid was filtered off, washed with ethanol, and recrystallized from water (twice) to give the title compound **20** (brown-orange solid, yield 2.76 g, 82%, m. p. 139–140 °C (water)). ^1^H-NMR (400 MHz, DMSO-d_6_): δ = 2.55 (s, 3H, CH_3_), 2.70–2.88 (m, 2H, COCH_2_), 3.29–3.45 (m, 1H, CH), 3.95–4.16 (m, 2H, NCH_2_), 5.98 (br s, 3H, OH + NH_2_), 6.31–6.44 (m, 2H, H_Ar_), 6.44–6.63 (m, 2H, H_Ar_), 7.81 (d, *J* = 8.6 Hz, 2H, H_Ar_), 7.97 (d, *J* = 8.6 Hz, 2H, H_Ar_) ppm. ^13^C-NMR (101 MHz, DMSO-d_6_): δ = 26.5 (CH_3_), 35.1 (COCH_2_), 35.4 (CH), 49.8 (NCH_2_), 114.6, 117.4, 118.4, 129.2, 132.1, 134.9, 143.1 (C_Ar_), 172.6, 174.1 (2C=O), 196.7 (H_3_C–C=N) ppm. Calcd. for C_19_H_19_N_3_O_3_, %: C 67.64; H 5.68; N 12.46. Found, %: C 67.76; H 5.61; N 12.42.

*5-Oxo-1-(4-(quinoxalin-2-yl)phenyl)pyrrolidine-3-carboxylic acid* (**21**): A mixture of compound **2** (1 g, 3.08 mmol) and benzene-1,2-diamine (0.3 g, 2.77 mmol) in ethanol (12 mL) was refluxed for 5 h, then cooled down, the formed crystalline solid was filtered off, washed with methanol, and recrystallized from propan-2-ol to give the title compound **21** (brown solid, yield 0.72 g, 81%, m. p. 277–278 °C (propan-2-ol)). ^1^H-NMR (400 MHz, DMSO-d_6_): δ = 2.72–2.92 (m, 2H, COCH_2_), 3.36–3.45 (m, 1H, CH), 4.04–4.17 (m, 2H, NCH_2_), 7.78–7.92 (m, 4H, H_Ar_), 8.04–8.15 (m, 2H, H_Ar_), 8.36 (d, J =8.9 Hz, 2H, H_Ar_), 9.56 (s, 1H, CH), 12.85 (s, 1H, OH) ppm. ^13^C-NMR (101 MHz, DMSO-d_6_): δ = 35.1 (COCH_2_), 36.4 (CH), 49.9 (NCH_2_), 119.3, 127.9, 128.8, 129.1, 129.6, 130.6, 131.2, 140.9, 141.1, 141.4, 143.5, 150.3 (C_Ar_), 172.3 (C=O), 174.1 (COOH) ppm. IR (KBr): ν_max_ = 3415 (OH), 3193 (NH), 1732, 1652 (2C=O). Anal Calcd for C_19_H_15_N_3_O_3_ *m*/*z* %: 334.1191 [M + H]^+^, found HRMS (ESI), *m*/*z* %: 334.1193 [M + H]^+^ (100%). Calcd. for C_19_H_15_N_3_O_3_, %: C 68.46; H 4.54; N 12.61. Found, %: 68.41; H 4.64; N 12.72.

*1-(4-Acetylphenyl)-3-(1H-benzimidazol-2-yl)-5-oxopyrrolidine* (**22**): A mixture of compound **1** (1 g, 4 mmol), 1,2-benzenediamine (0.86 g, 8 mmol) and 4N hydrochloric acid (15 mL) was heated at reflux for 16 h. After completion of the reaction (TLC), the reaction mixture was filtered off, the solvent was evaporated under reduced pressure, and the residue was neutralized with 25% aqueous ammonia to pH 7. Then, a liquid layer was decanted, the precipitate was poured over with 5% aqueous sodium carbonate solution (20 mL) and afterward boiled and filtered off until hot. The obtained crystalline solid was washed with plenty of water and recrystallized from methanol to give the title compound **22** (yellow solid, yield 1.17 g, 92%, m. p. 222–223 °C). ^1^H-NMR (400 MHz, DMSO-d_6_): δ = 2.55 (s, 3H, CH_3_), 2.99–3.14 (m, 2H, COCH_2_), 3.98–4.08 (m, 1H, CH), 4.25–4.38 (m, 2H, NCH_2_), 7.13–7.18 (m, 2H, H_Ar_), 7.49–7.55 (m, 2H, H_Ar_), 7.85 (d, *J* = 8.7 Hz, 2H, H_Ar_), 7.99 (d, *J* = 8.7 Hz, 2H, H_Ar_) ppm. ^13^C-NMR (101 MHz, DMSO-d_6_): δ = 26.5 (CH_3_), 30.6 (COCH_2_), 37.7 (CH), 52.0 (NCH_2_), 114.8 118.4, 121.5, 129.2, 132.1, 138.8, 143.2 (C_Ar_), 155.0 (NHC=N), 172.8 (C=O), 196.6 (H_3_C–C=O) ppm. IR (KBr): ν_max_ = 3056 (NH), 1699, 1683 (2C=O), 1601 (C=N). Calcd. for C_19_H_17_N_3_O_2_, %: C 71.46; H 5.37; N 13.16. Found, %: C 71.26; H 5.31; N 13.09.

*1-(4-Acetylphenyl)-3-(1-ethyl-1H-benzimidazol-2-yl)-5-oxopyrrolidine* (**23**): A mixture of benzimidazole **22** (1 g, 3 mmol), potassium hydroxide (0.36 g, 6.4 mmol), sodium carbonate (0.21 g, 2 mmol) and ethyl iodide (10 mL) was stirred at room temperature for 48 h. After completion of the reaction (TLC), a mixture was poured over with dry acetone and filtered off. The filtrate was evaporated under reduced pressure to dryness, and then the obtained oily mass was poured over with diethyl ether and left in refrigerator for several hours. The formed crystalline solid was filtered off, washed with diethyl ether, and recrystallized from methanol to give the title compound **23** (light yellow solid, yield 0.96 g, 88%, m. p. 175–176 °C). ^1^H-NMR (400 MHz, DMSO-d_6_): δ = 1.30 (t, *J* = 7.1 Hz, 3H, CH_3_), 2.96–3.18 (m, 2H, COCH_2_), 4.13–4.45 (m, 5H, CH + NCH_2_ + CH_3_CH_2_), 7.11–7.291 (m, 2H, H_Ar_), 7.50–7.66 (m, 2H, H_Ar_), 7.82 (d, *J* = 8.8 Hz, 2H, H_Ar_), 7.94 (d, *J* = 8.8 Hz, 2H, H_Ar_) ppm. ^13^C-NMR (101 MHz, DMSO-d_6_): δ = 15.2 (CH_3_), 26.5 (CH_3_), 28.4 (COCH_2_), 37.9 (CH + CH_3_CH_2_), 52.0 (NCH_2_), 110.2, 118.5, 118.8, 121.5, 122.0, 129.2, 132.1, 135.0, 142.0, 143.2 (C_Ar_), 154.4 (N–C=N), 172.7 (N–C=O), 196.6 (H_3_C–C=O) ppm. IR (KBr): ν_max_ = 1707, 1670 (2C=O). Calcd. for C_21_H_21_N_3_O_2_, %: C 72.60; H 6.09; N 12.10. Found, %: C 71.72; H 5.92; N 12.04.

### 3.2. Biological Activity

The determination of the antibacterial and antifungal activity by diffusion method in agar was carried out by diffusion in agar on a solid nutrient medium (beef-extract agar for bacteria, wort agar for fungi). Petri plates containing 20 mL of nutrient medium were used for all the tested microorganisms. The inoculum (the microbial loading 109 cells (spores)/1 mL) was spread on the surface of the solidified media and Whatman no. 1 filter paper discs (6 mm in diameter) impregnated with the test compound (0.1 and 0.5%) were placed on the plates. The duration of bacteria incubation was 24 h at 35 °C and that of fungi incubation was 48–72 h at 28–30 °C. The antimicrobial effect and degree of activity of the tested compounds were evaluated by measuring the inhibition zone diameters and the results were compared with the data for the well-known antibacterial/antifungal agent. Each experiment was repeated three times. The determination of minimal inhibitory (MIC) concentrations was achieved using the serial dilution method. The tested compounds were added to the nutrient medium (beef-extract broth for bacteria and wort for fungi) as solutions in dimethyl sulfoxide (DMSO) for ensuring the needed concentration (0.9–500.0 μg/mL). Bacteria and fungi inoculum were inoculated into nutrient medium (the microbial loading was 106 cells (spores)/1 mL). The duration of bacteria incubation was 24 h at 35 °C and that of fungi incubation was 48–72 h at 28–30 °C. The results were estimated by the microorganism growth measured by degree of microbial turbidity in nutrient medium. The MIC of any compound was defined as the lowest concentration, which completely inhibits visible growth (turbidity on liquid nutrient medium).

## 4. Conclusions

In this study, the condensation of 1-(4-(2-bromoacetyl)phenyl)-5-oxopyrrolidine-3-carboxylic acid with thiourea, benzenecarbothioamide and thioureido acid novel disubstituted thiazole derivatives were obtained. Some transformations of these compounds were carried out; compounds with pyrrolidine, pyrrole, triazole, oxadiazole fragments were synthesized; and their antibacterial properties were investigated. The antibacterial assay revealed that in most cases substituted phenylthiazole derivatives had stronger inhibition and bactericidal properties against the test-cultures *Staphylococcus aureus* (ATCC 25923), *Bacillus cereus* (ATCC 10231), *Listeria monocytogenes* (ATCC 19111), *Pseudomonas aeruginosa* (ATCC 10145), *Escherichia coli* (ATCC 8739) and *Salmonella enterica enteritidis* when compared with the corresponding aminothiazoles. Compounds **3c**, **5b**, **15b** and **16 b** were found to have an exceptional antibacterial potency against all tested bacteria strains. The MIC of these compounds was observed at 7.8 μg/mL, and MBC – at 15.6 μg/mL, while the minimum inhibitory and minimum bactericidal concentrations of *Oxytetracycline* for Gram-positive bacteria strains was determined to be 62.5 μg/mL, and 125 μg/mL for Gram-negative ones.

## Figures and Tables

**Table 1:** The yields of the synthesized compounds **2**–**23**.

Compound	Yield, %	Compound	Yield, %	Compound	Yield, %
**1**	86	**7a**	90	**14b**	54
**2**	95	**7b**	69	**15b**	86
**3a**	62	**8a**	62	**16b**	90
**3b**	43	**8b**	89	**17b**	76
**3c**	82	**9b**	82	**18b**	67
**4a**	62	**10b**	75	**19b**	88
**4b**	71	**11b**	99	**20**	82
**5a**	81	**12b**	96	**21**	81
**5b**	65	**13a**	57	**22**	92
**6a**	90	**13b**	92	**23**	88
**6b**	92	**-**	-	**-**	-

**Table 2:** The determined MIC and MBC of the synthesized compounds (only the data for compounds that gave positive results in at least one case are included in the table).

Compound	Gram-Positive Bacteria Strains						Gram-Negative Bacteria Strains					
	*S. aureus*		*B. cereus*		*L. monocytogenes*		*P. aeruginosa*		*E. coli*		*S. enterica enteritidis*	
	MIC	MBC	MIC	MBC	MIC	MBC	MIC	MBC	MIC	MBC	MIC	MBC
	µg/mL											
**3a**	15.6	31.25	31.25	62.5	31.25	62.5	31.25	62.5	31.25	62.5	31.25	62.5
**3b**	31.25	62.5	15.6	31.25	15.6	31.25	15.6	31.25	62.5	125	15.6	31.25
**3c**	7.8	15.6	7.8	15.6	7.8	15.6	7.8	15.6	7.8	15.6	7.8	15.6
**4a**	62.5	125	31.25	62.5	31.25	62.5	31.25	62.5	31.25	62.5	31.25	62.5
**4b**	15.6	31.25	15.6	31.25	15.6	31.25	15.6	31.25	15.6	31.25	15.6	31.25
**5a**	31.25	62.5	31.25	62.5	31.25	62.5	31.25	62.5	31.25	62.5	31.25	62.5
**5b**	7.8	15.6	7.8	15.6	7.8	15.6	7.8	15.6	7.8	15.6	7.8	15.6
**6a**	31.25	62.5	31.25	62.5	31.25	62.5	31.25	62.5	31.25	62.5	31.25	62.5
**6b**	15.6	31.25	15.6	31.25	15.6	31.25	15.6	31.25	15.6	31.25	15.6	31.25
**7b**	+	-	7.8	15.6	15.6	31.25	+	-	15.6	31.25	15.6	15.6
**8b**	31.25	62.5	31.25	62.5	31.25	62.5	31.25	62.5	15.6	31.25	31.25	62.5
**9b**	7.8	15.6	15.6	31.25	15.6	31.25	15.6	31.25	15.6	31.25	15.6	31.25
**11b**	62.5	125	+	-	15.6	31.25	15.6	31.25	31.25	62.5	15.6	31.25
**12b**	31.25	62.5	31.25	62.5	31.25	62.5	31.25	62.5	31.25	62.5	31.25	62.5
**13a**	31.25	62.5	31.25	62.5	31.25	62.5	31.25	62.5	31.25	62.5	31.25	62.5
**13b**	31.25	62.5	15.6	31.25	15.6	31.25	15.6	31.25	31.25	62.5	15.6	31.25
**14b**	7.8	15.6	62.5	125	125	250	125	250	125	250	+	-
**15b**	7.8	15.6	7.8	15.6	7.8	15.6	7.8	15.6	7.8	15.6	7.8	15.6
**16b**	7.8	15.6	7.8	15.6	7.8	15.6	7.8	15.6	7.8	15.6	7.8	15.6
**17b**	15.6	31.25	15.6	31.25	15.6	31.25	15.6	31.25	15.6	31.25	15.6	31.25
**21**	62.5	125	31.25	31.25	31.25	62.5	-	-	125	125	31.25	62.5
**22**	31.25	31.25	31.25	62.5	-	-	31.25	62.5	31.25	31.25	-	-
**23**	15.6	31.25	31.25	62.5	-	-	31.25	62.5	31.25	31.25	-	-
***C***	**62.5**						**125**					

+, the growth of microorganisms; -, the absence of growth inhibition and bactericidal action; ***C***, control antibiotic *Oxytetracycline*.

## Supplementary Materials

The following are available online. Figure S1: 1H-NMR of compound 2, Figure S2: ^13^C-NMR of compound 2, Figure S3: 1H-NMR of compound 3 a, Figure S4: 13C-NMR of compound 3 a, Figure S5: 1H-NMR of compound 3 b, Figure S6: ^13^C-NMR of compound 3 b, Figure S7: 1H-NMR of compound 3 c, Figure S8: 13C-NMR of compound 3 c, Figure S9: ^1^H-NMR of compound 4 a, Figure S10: 13C-NMR of compound 4 a, Figure S11: 1H-NMR of compound 4 b, Figure S12: 13C-NMR of compound 4 b, Figure S13: 1H-NMR of compound 5 a, Figure S14: 13C-NMR of compound 5 a, Figure S15: 1H-NMR of compound 5 b, Figure S16: 13C-NMR of compound 5 b, Figure S17: 1H-NMR of compound 6 a, Figure S18: 1H-NMR of compound 6 b, Figure S19: 1H-NMR of compound 7 a, Figure S20: 1H-NMR of compound 7 b, Figure S21: 1H-NMR of compound 8 a, Figure S22: 1H-NMR of compound 8 b, Figure S23: 1H-NMR of compound 9 b, Figure S24: 1H-NMR of compound 10 b, Figure S25: 1H-NMR of compound 11 b, Figure S26: 1H-NMR of compound 12 b, Figure S27: 1H-NMR of compound 13 a, Figure S28: 13C-NMR of compound 13 a, Figure S29: 1H-NMR of compound 13 b, Figure S30: 13C-NMR of compound 13 b, Figure S31: 1H-NMR of compound 14 b, Figure S32: 13C-NMR of compound 14 b, Figure S33: 1H-NMR of compound 15 b, Figure S34: 13C-NMR of compound 15 b, Figure S35: 1H-NMR of compound 16 b, Figure S36: 13C-NMR of compound 16 b, Figure S37: 1H-NMR of compound 17 b, Figure S38: 13C-NMR of compound 17 b, Figure S39: 1H-NMR of compound 18 b, Figure S40: 13C-NMR of compound 18 b, Figure S41: 1H-NMR of compound 19 b, Figure S42: 13C-NMR of compound 19 b, Figure S43: 1H-NMR of compound 20, Figure S44: 13C-NMR of compound 20, Figure S45: 1H-NMR of compound 21, Figure S46: 13C-NMR of compound 21, Figure S47: 1H-NMR of compound 22, Figure S48: 13C-NMR of compound 22.
