# Supplementary material for: Synthesis, Characterization and Bioassay of Novel Substituted 1-(3-(1,3-Thiazol-2-yl)phenyl)-5-oxopyrrolidines
Source: Molecules. 2020 May 22;25(10):2433. doi: 10.3390/molecules25102433 (PMC7288019; doi:10.3390/molecules25102433)
Supplement: Supplementary file 1 [file molecules-25-02433-s001.pdf]

molecules

Supplementary Material

Synthesis, characterization, and bioassay of novel substituted 1-(3-(1,3-thiazol-2-yl)phenyl)-5-oxopyrrolidines

Birutė Sapijanskaitė-Banevič [1], Božena Šovkovaja [1], Rita Vaickelionienė [1,*], Jūratė Šiugždaitė [2] and Eglė Mickevičiūtė [3]

[1] Department of Organic Chemistry, Kaunas University of Technology, Radvilėnų Rd. 19, LT-50254, Kaunas, Lithuania

[2] Department of Veterinary Pathobiology, Veterinary Academy, Lithuanian University of Health Sciences, Tilžės Str. 18, LT-47181, Kaunas, Lithuania

[3] Department of Information Systems, Kaunas University of Technology, Studentų Str. 50, LT-51368 Kaunas, Lithuania

* Author to whom correspondence should be addressed: rita.vaickelioniene@ktu.lt

1-(4-(2-Bromoacetyl)phenyl)-5-oxopyrrolidine-3-carboxylic acid (2)

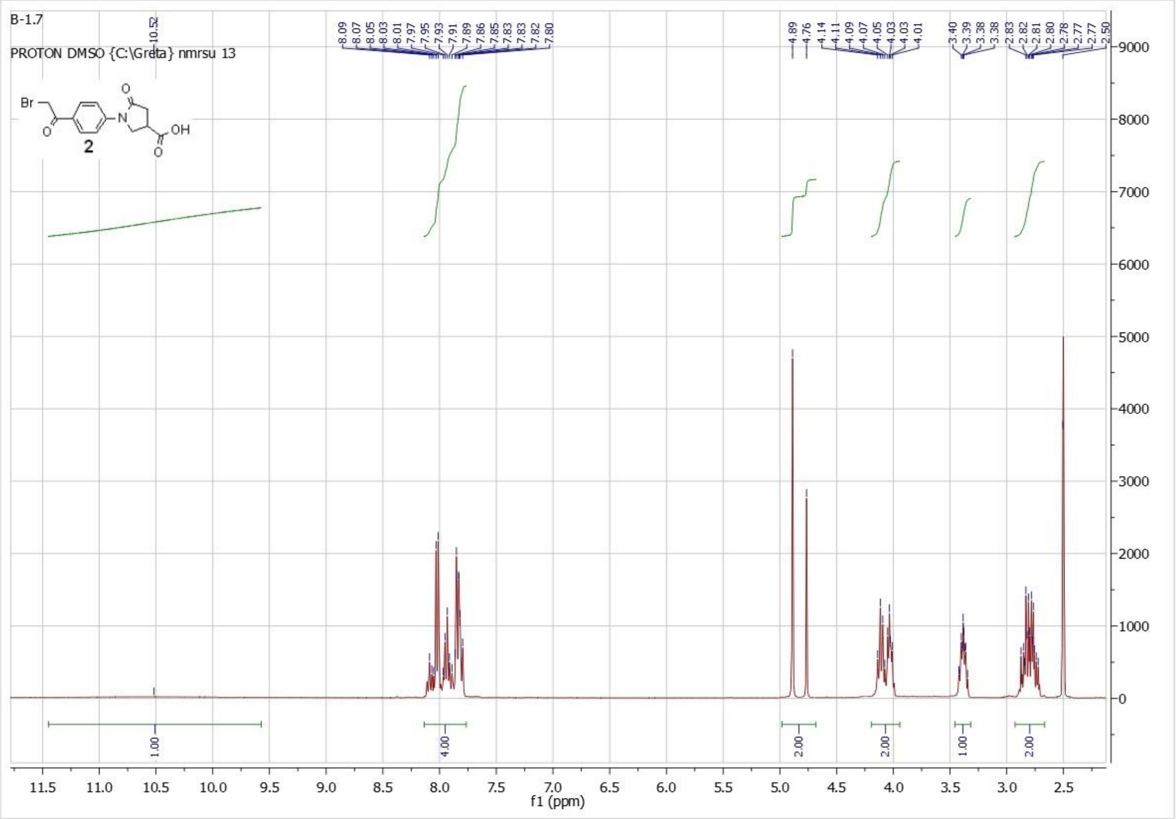

Figure S1: ^{1}H NMR of compound **2**

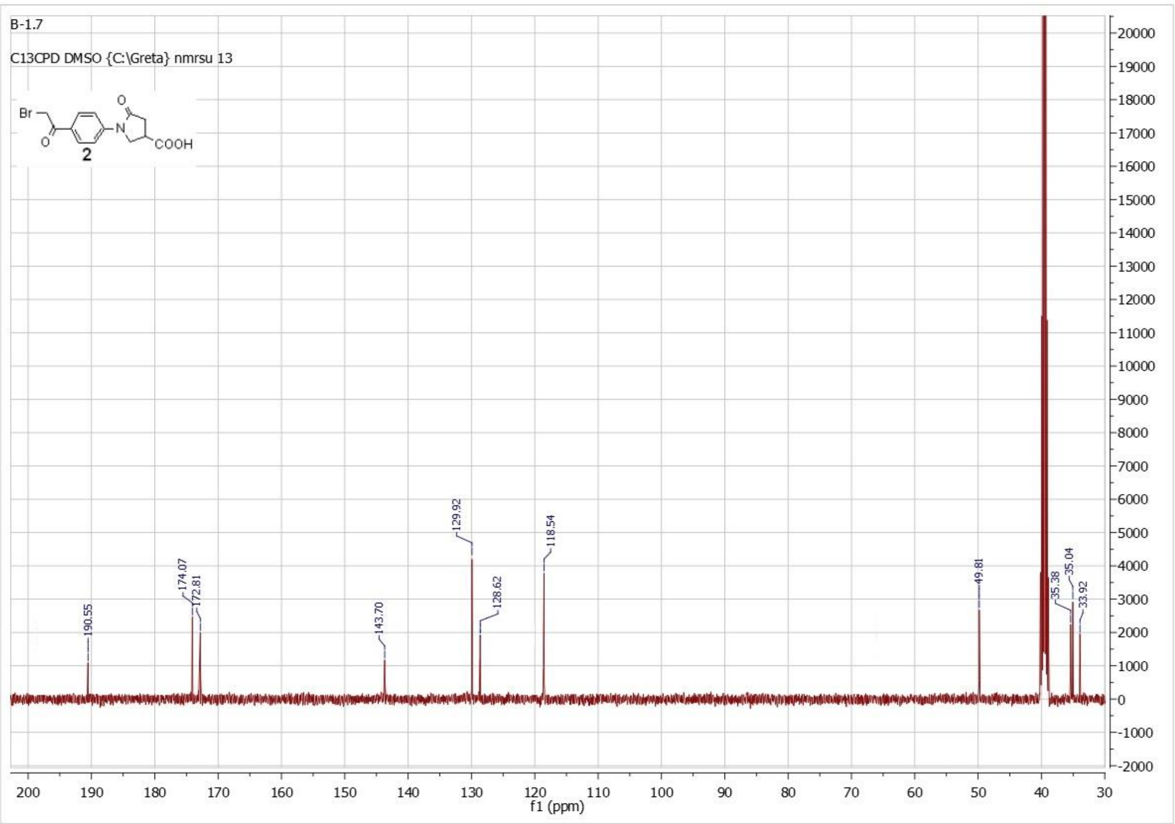

Figure S2: ^{13}C NMR of compound **2**

1-(4-(2-Aminothiazol-5-yl)phenyl)-5-oxopyrrolidine-3-carboxylic acid (3 a)

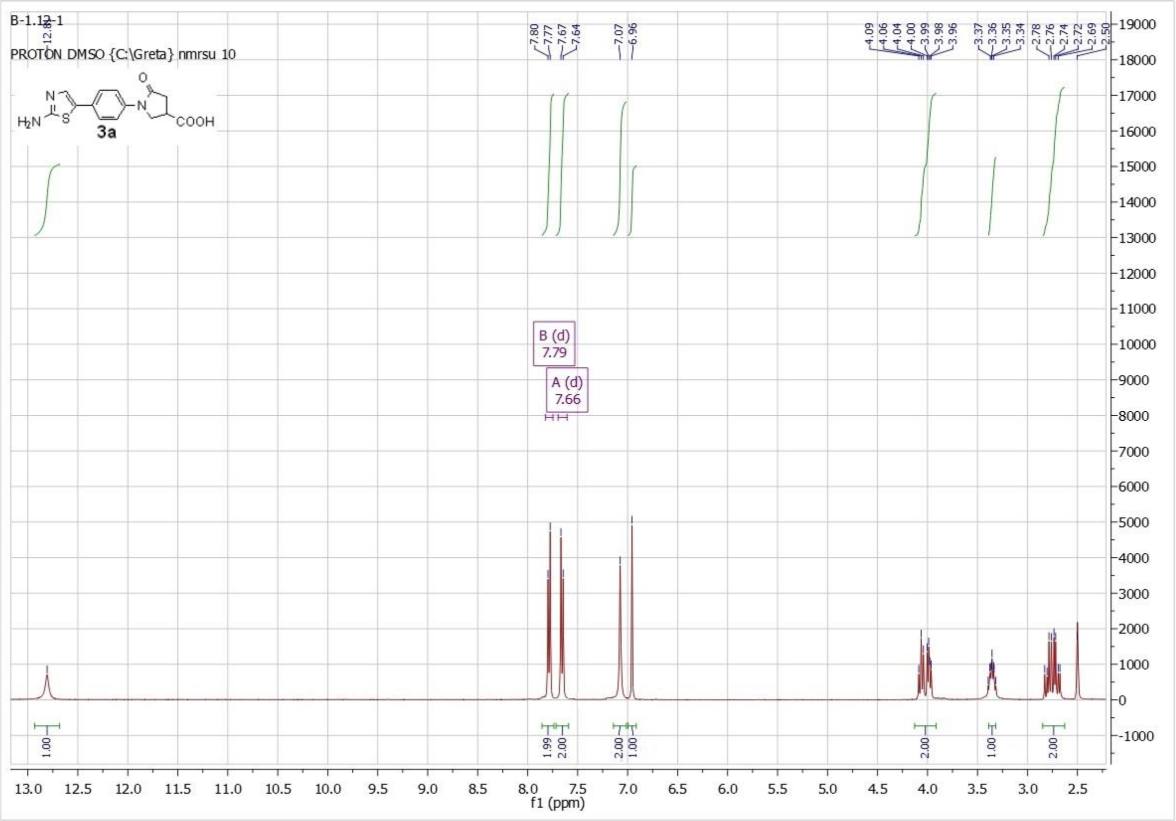

Figure S3: ^{1}H NMR of compound **3 a**

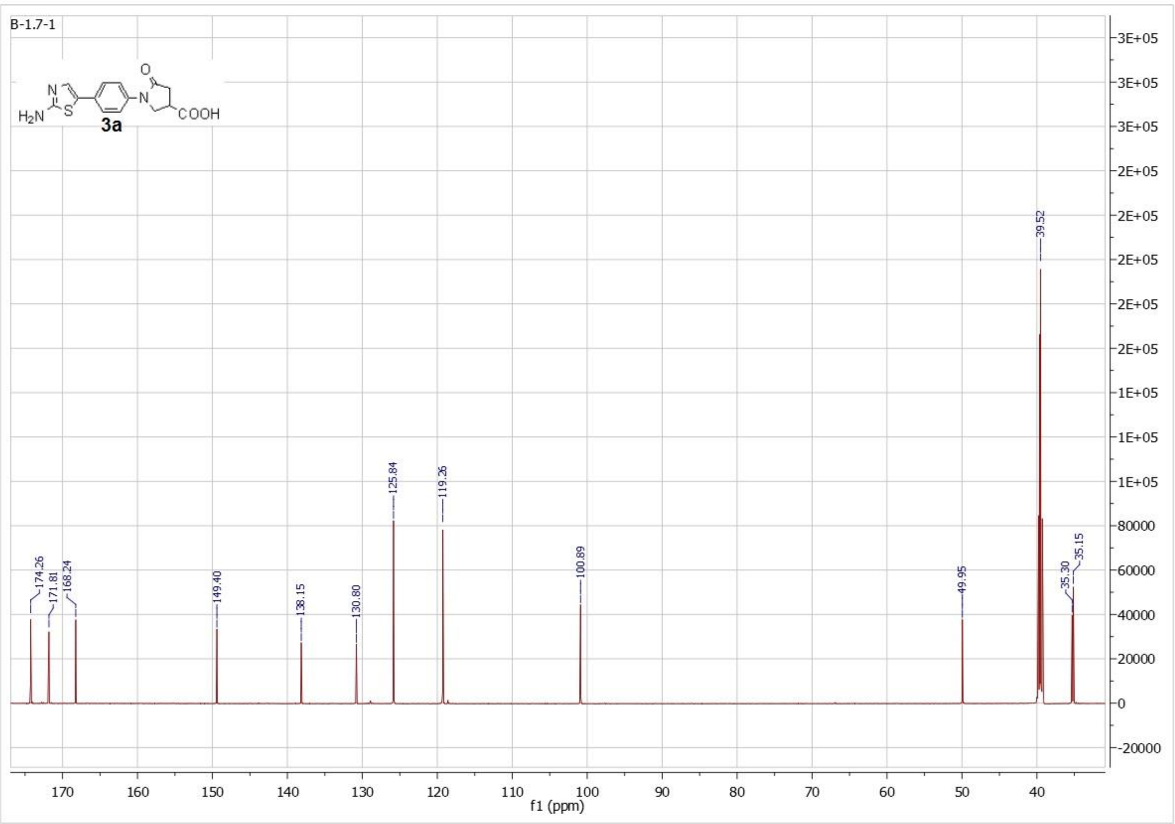

Figure S4: ^{13}C NMR of compound **3 a**

5-Oxo-1-(4-(2-phenylthiazol-5-yl)phenyl)pyrrolidine-3-carboxylic acid (3 b)

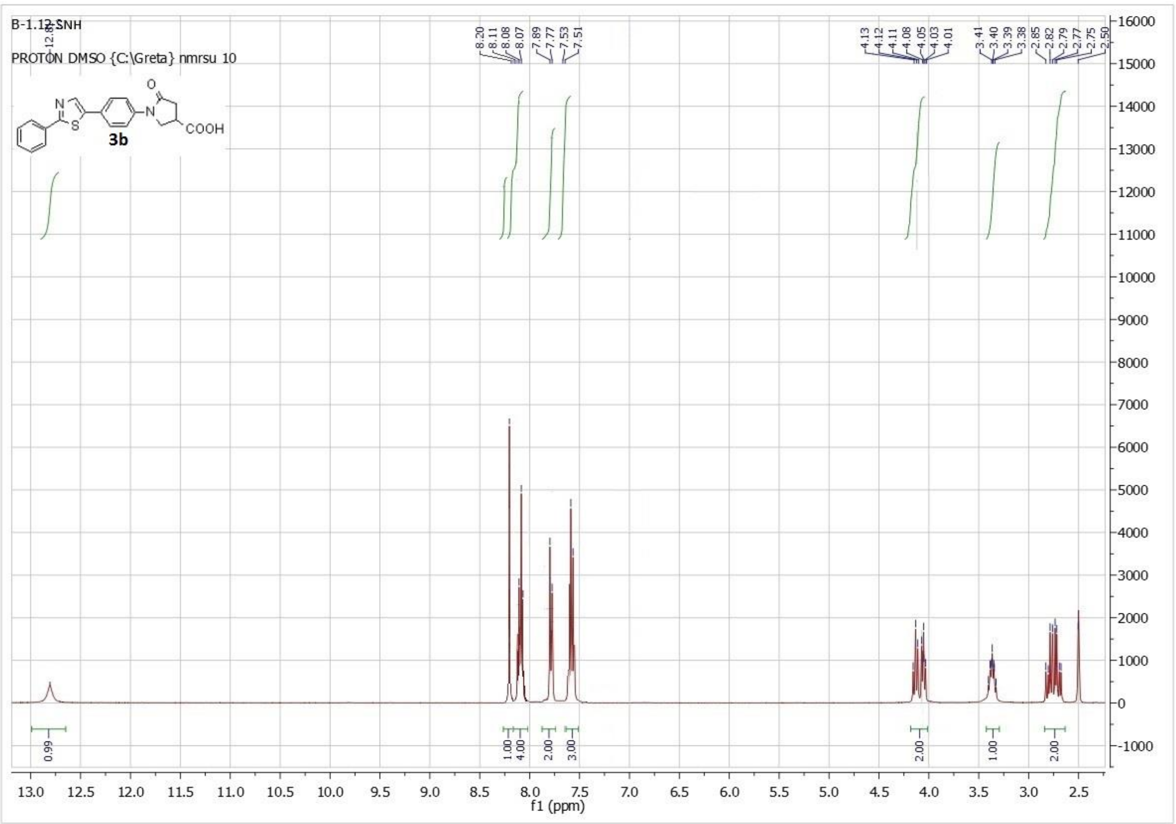

Figure S5: ^{1}H NMR of compound **3 b**

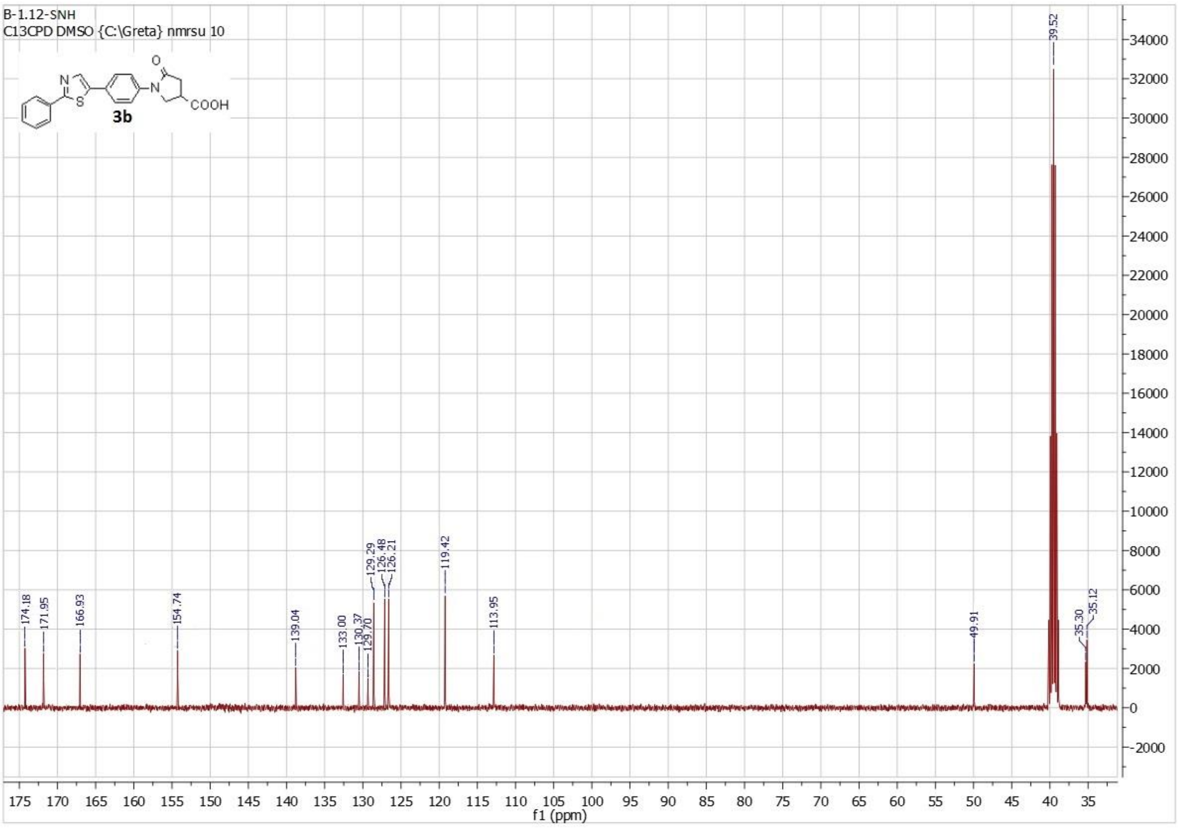

Figure S6: ^{13}C NMR of compound **3 b**

1-(4-(2-((2-Carboxyethyl)-4-methylanilino)-1,3-thiazol-5-yl)phenyl)-5-oxo-3-pyrrolidinecarboxylic acid (3 c)

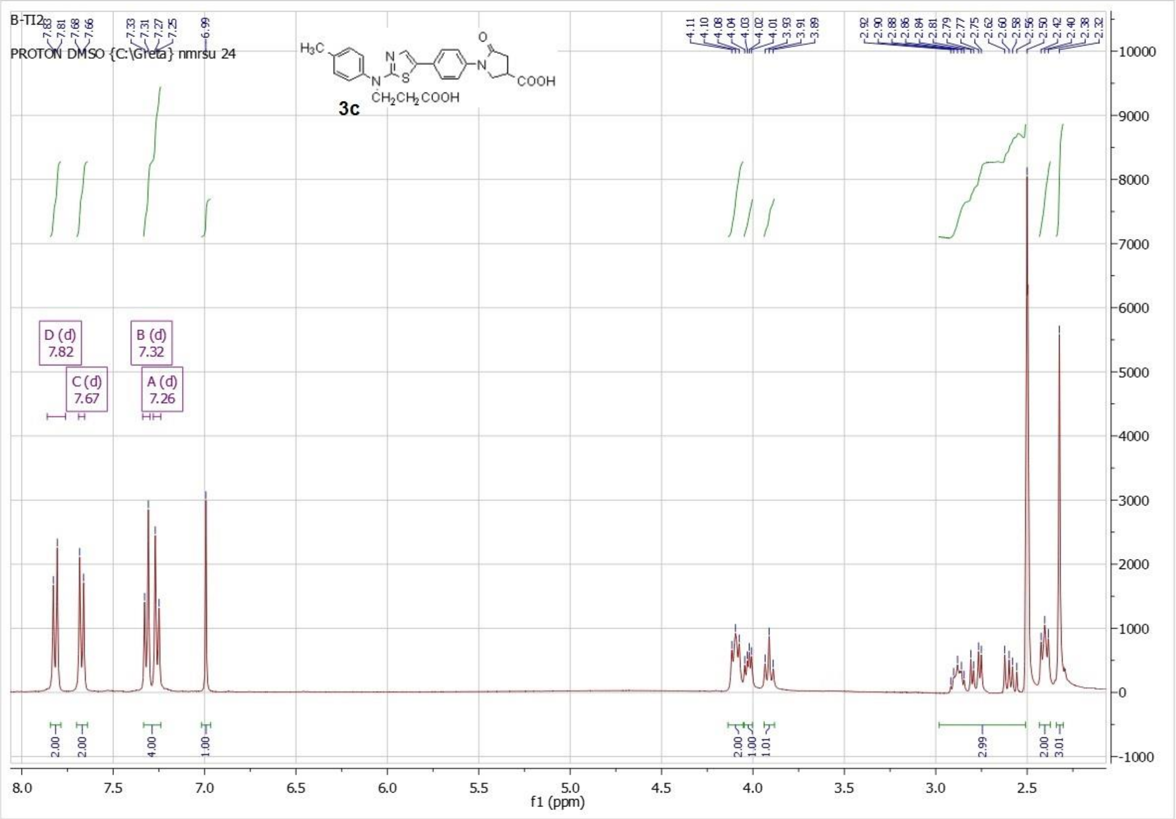

Figure S7: ^{1}H NMR of compound **3 c**

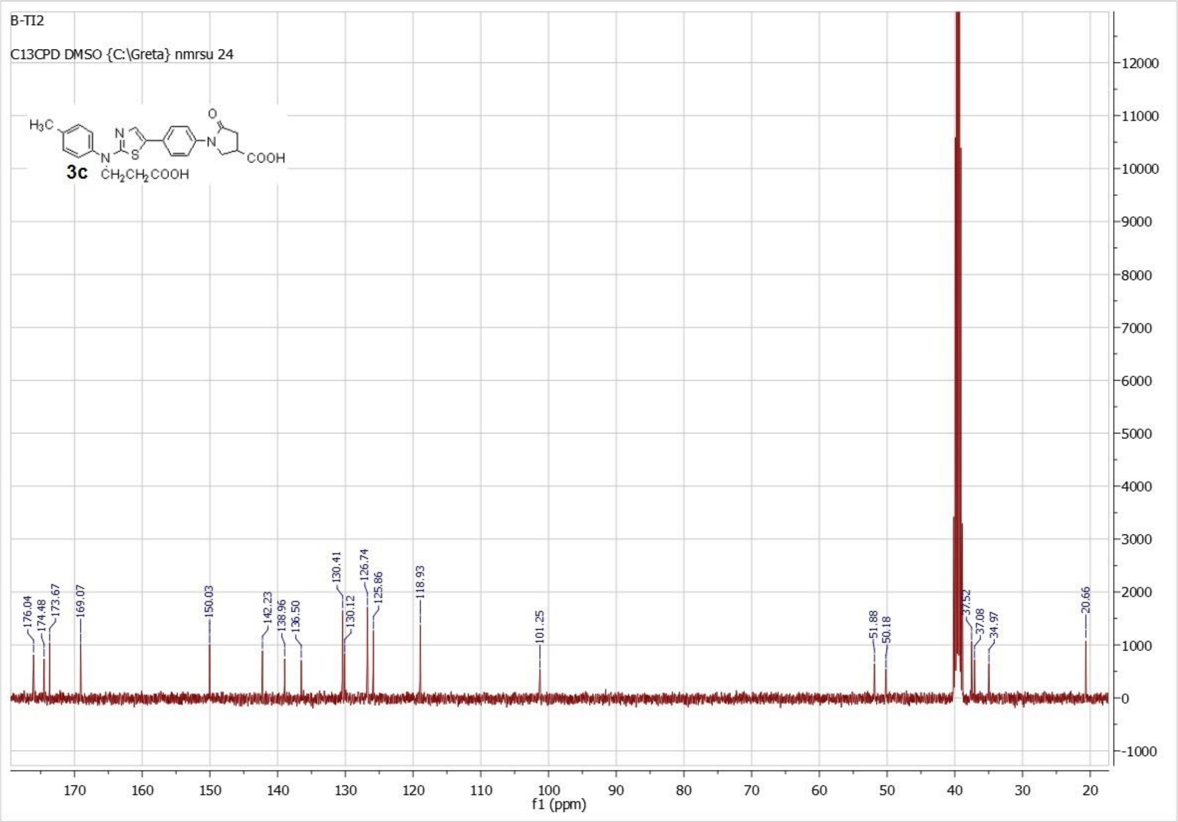

Figure S8: ^{13}C NMR of compound **3 c**

Methyl 1-(4-(2-aminothiazol-5-yl)phenyl)-5-oxopyrrolidine-3-carboxylate (4 a)

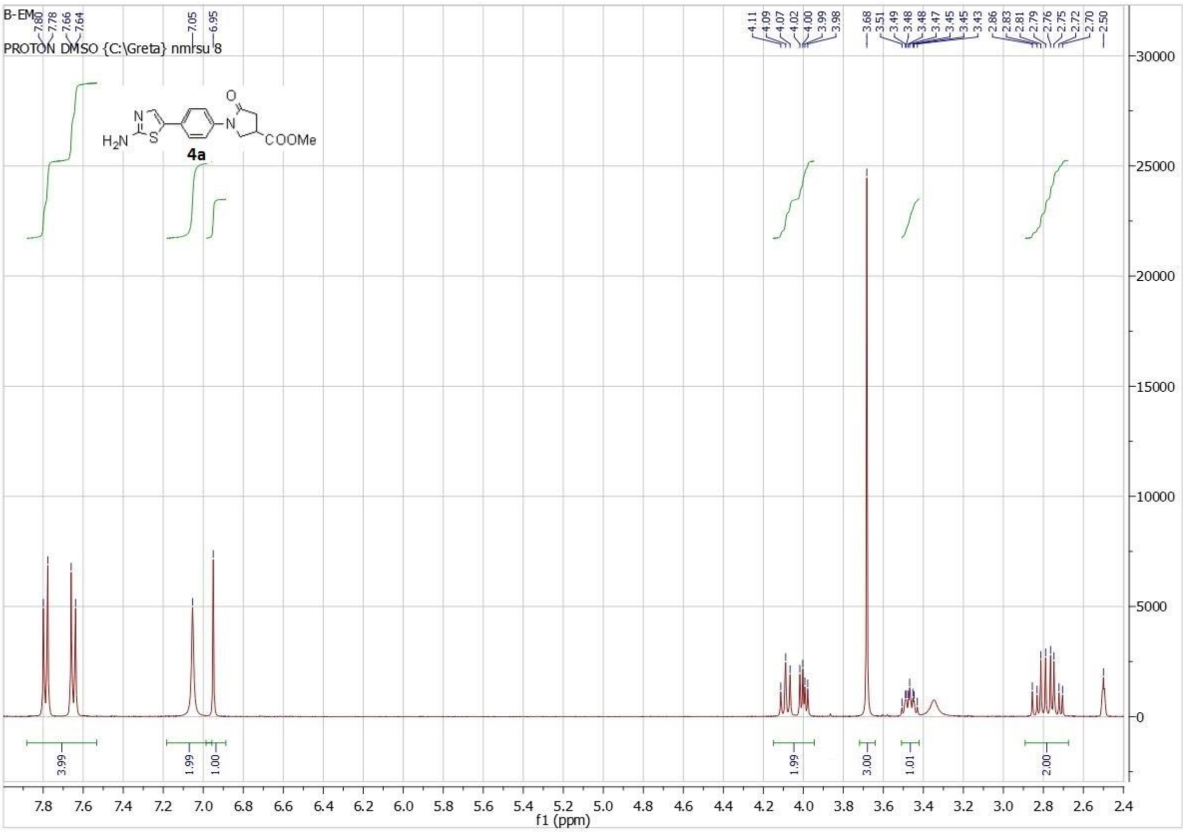

Figure S9: ^{1}H NMR of compound **4 a**

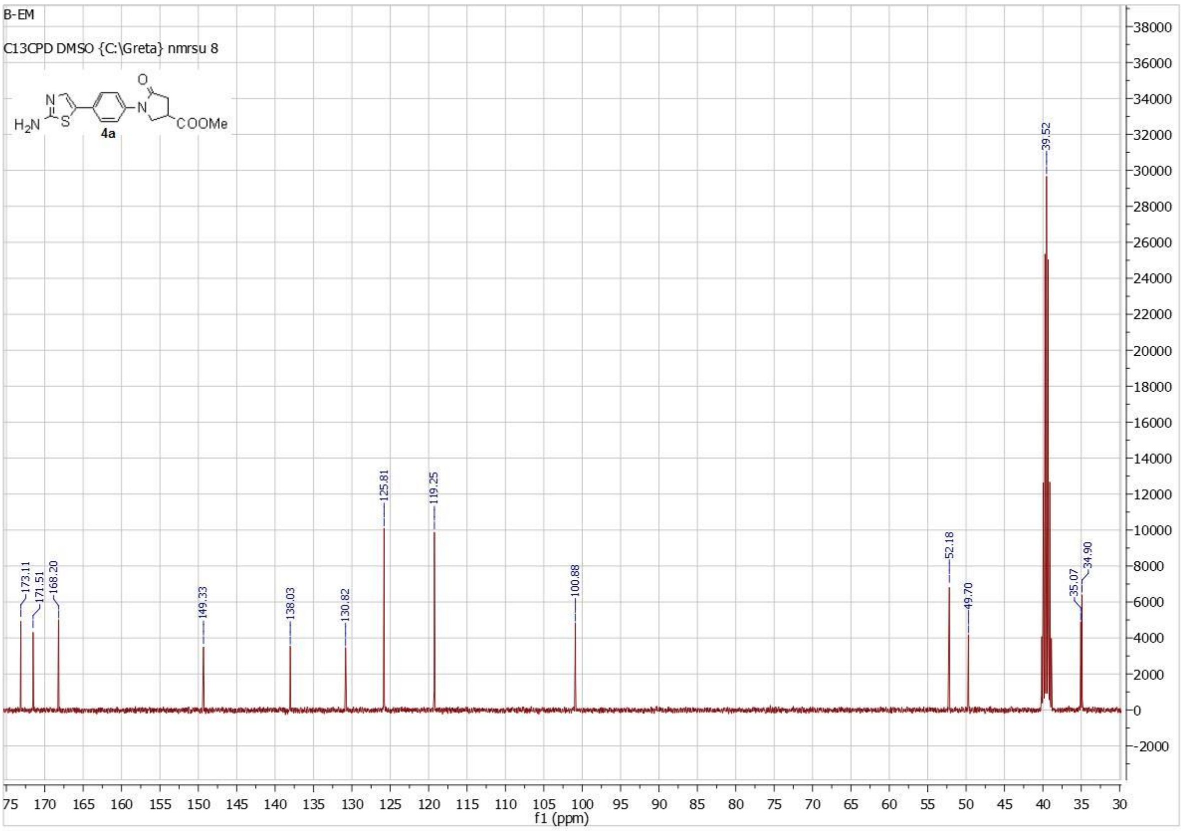

Figure S10: ^{13}C NMR of compound **4 a**

Methyl 5-oxo-1-(4-(2-phenylthiazol-5-yl)phenyl)pyrrolidine-3-carboxylate (4 b)

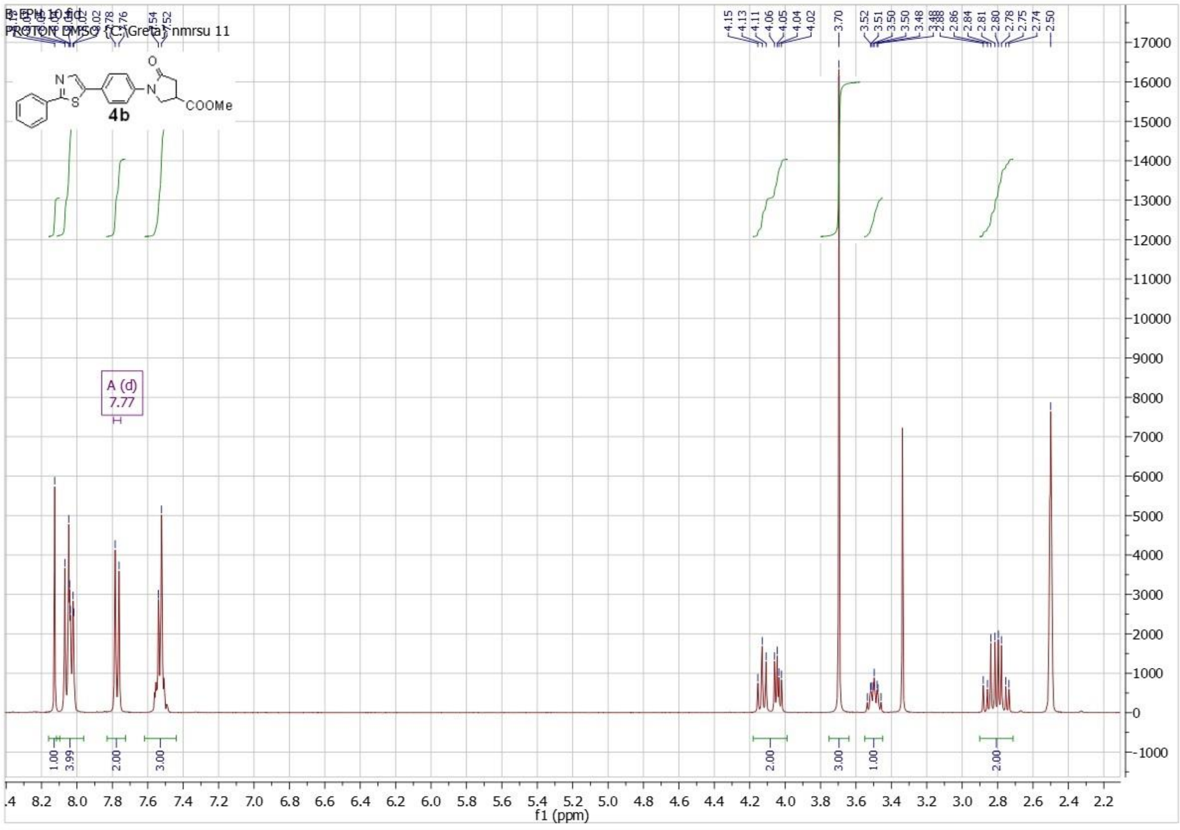

Figure S11: ^{1}H NMR of compound **4 b**

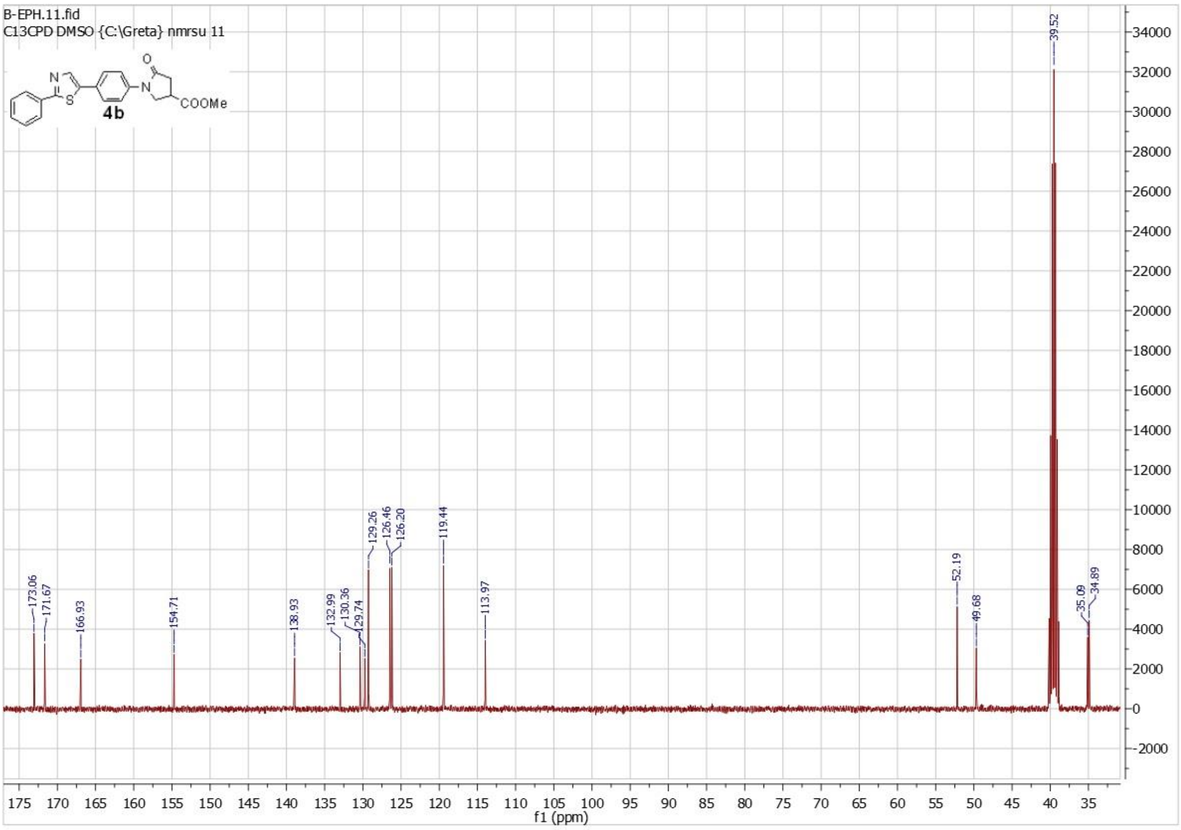

Figure S12: ^{13}C NMR of compound **4 b**

1-(4-(2-Aminothiazol-5-yl)phenyl)-5-oxopyrrolidine-3-carbohydrazide (5 a)

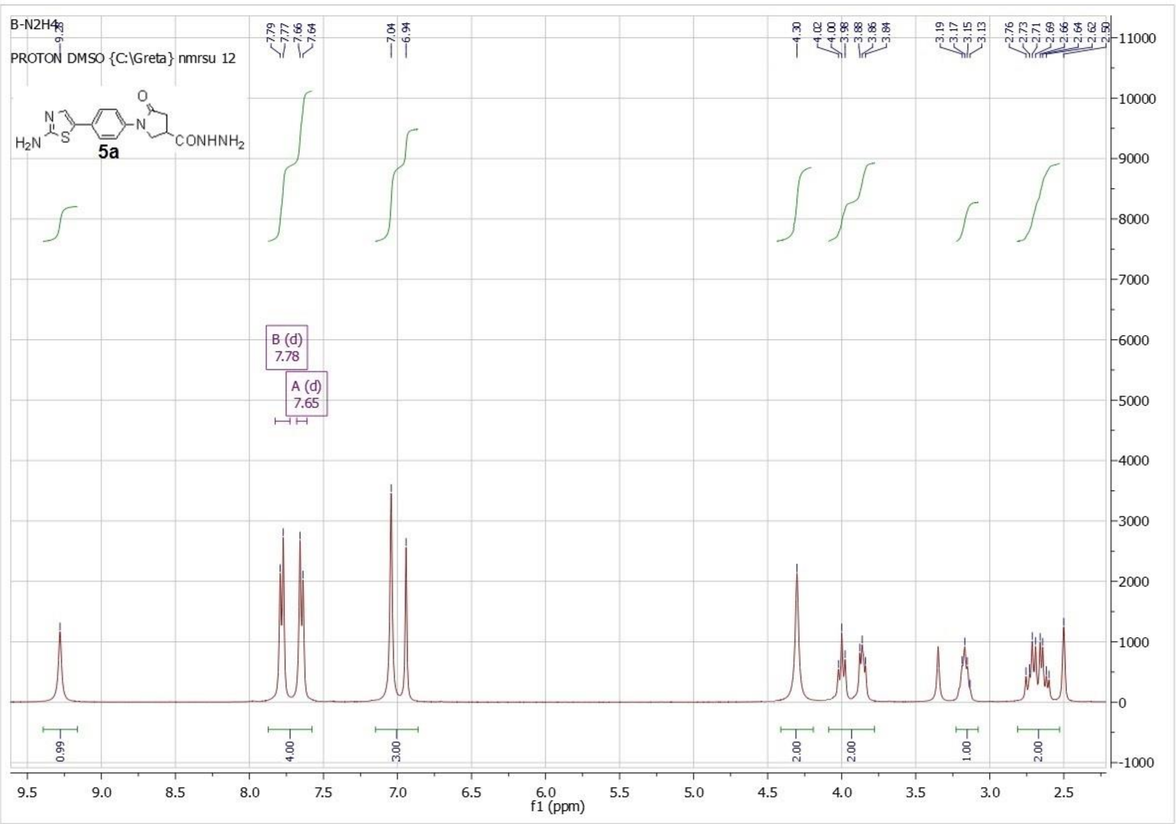

Figure S13: ^{1}H NMR of compound **5 a**

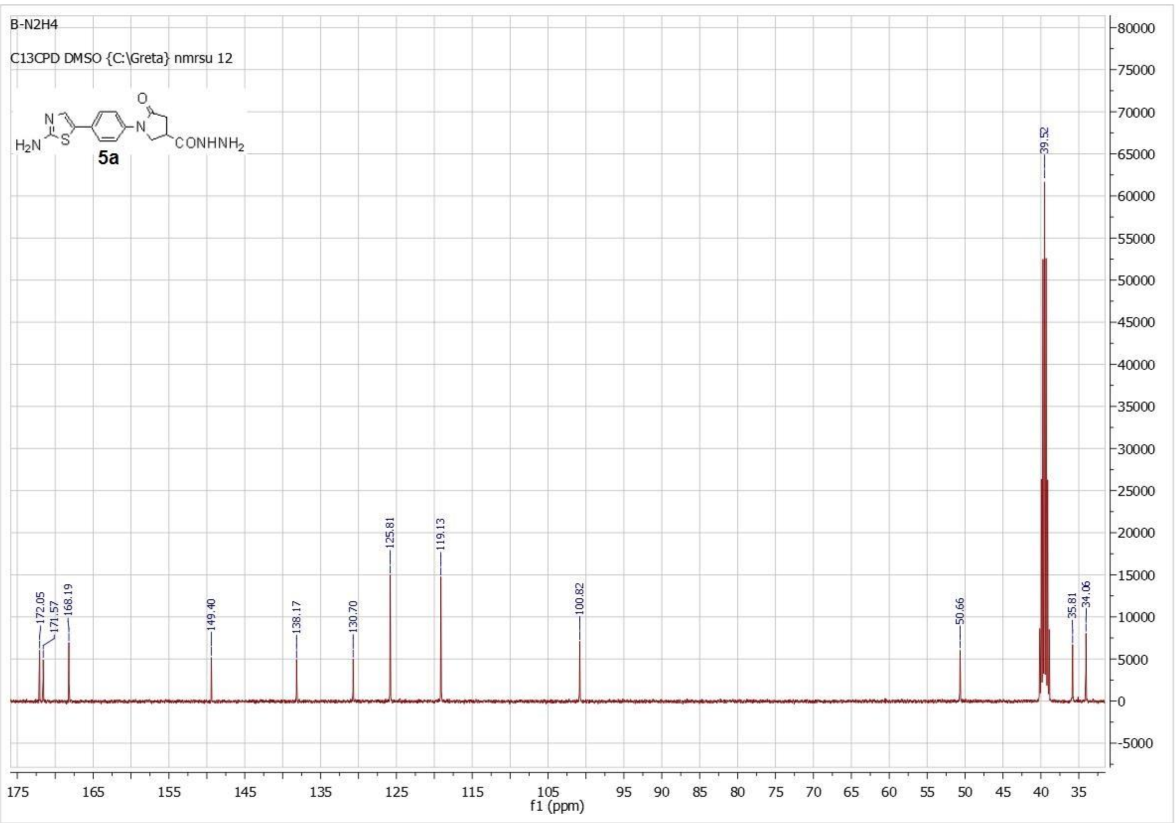

Figure S14: ^{13}C NMR of compound **5 a**

5-Oxo-1-(4-(2-phenylthiazol-5-yl)phenyl)pyrrolidine-3-carbohydrazide (5 b):

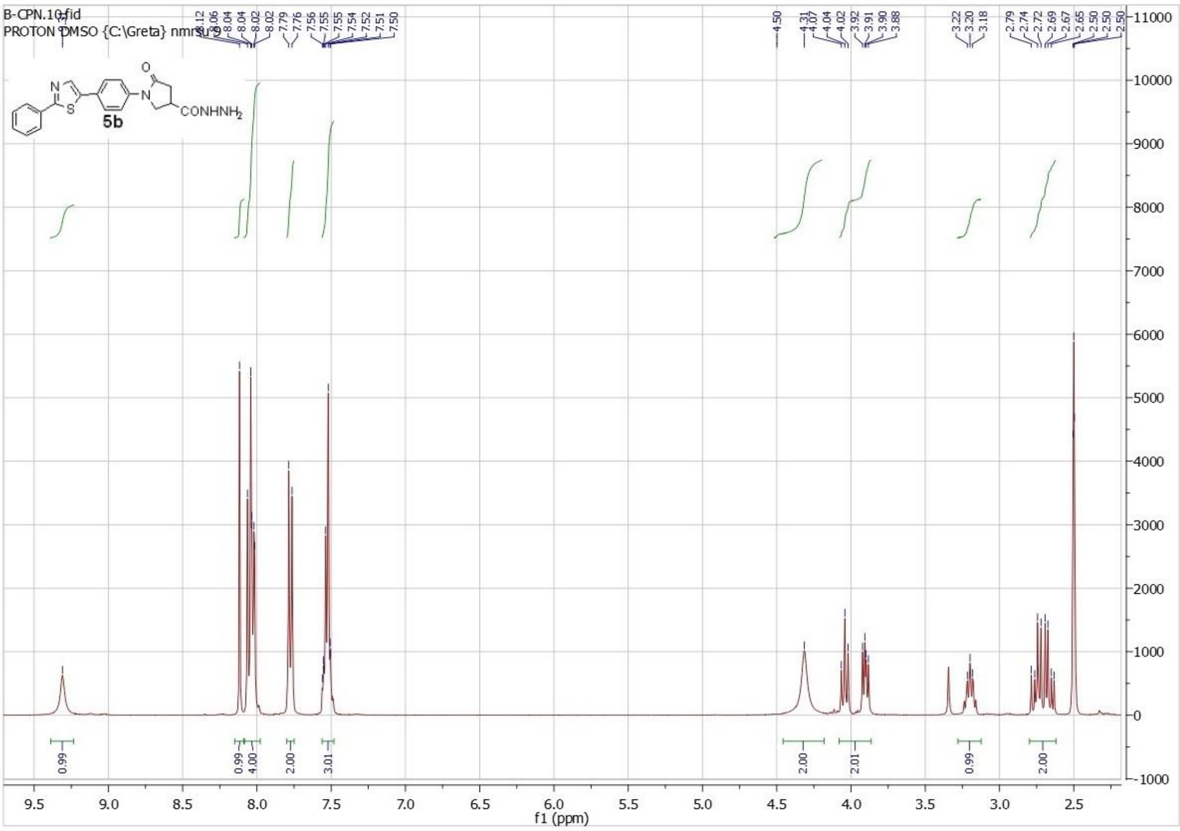

Figure S15: ^{1}H NMR of compound **5 b**

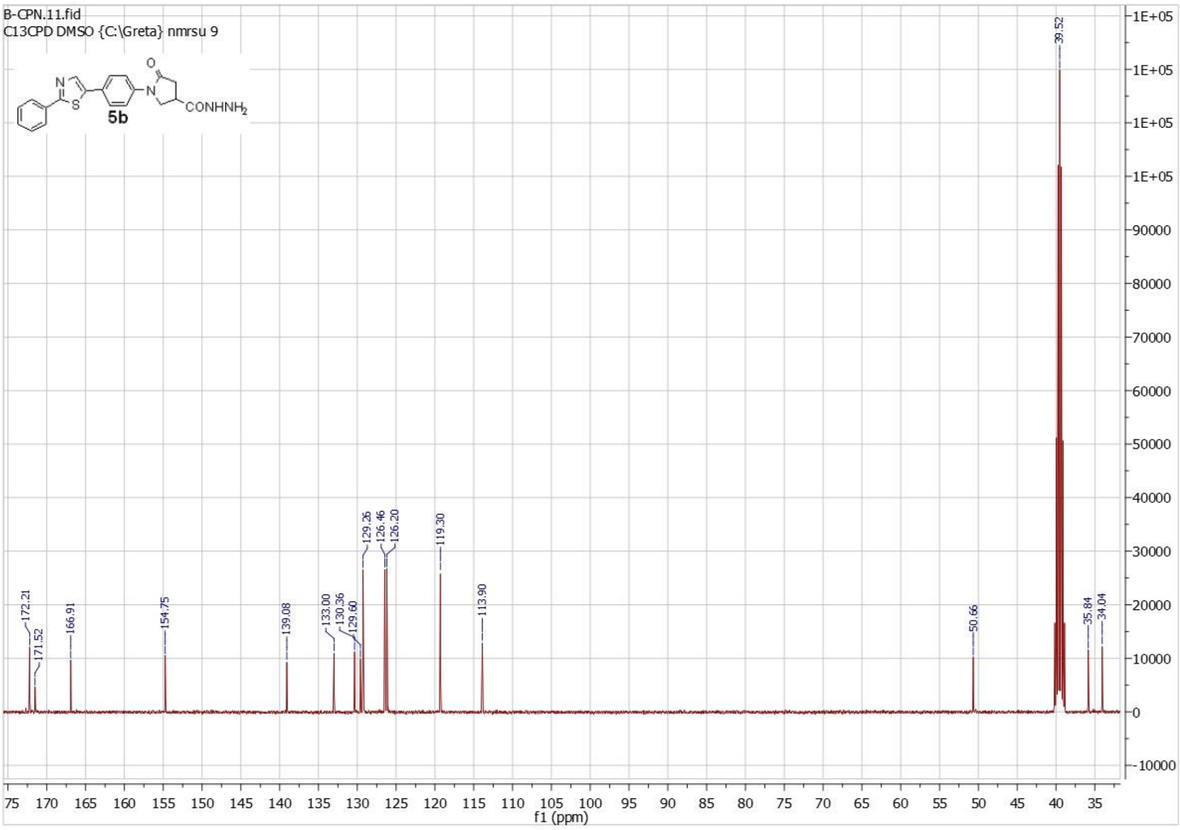

Figure S16: ^{13}C NMR of compound **5 b**

1-(4-(2-Aminothiazol-5-yl)phenyl)-3-(benzylidenehydrazinocarbonyl)-5-oxopyrrolidine (6 a)

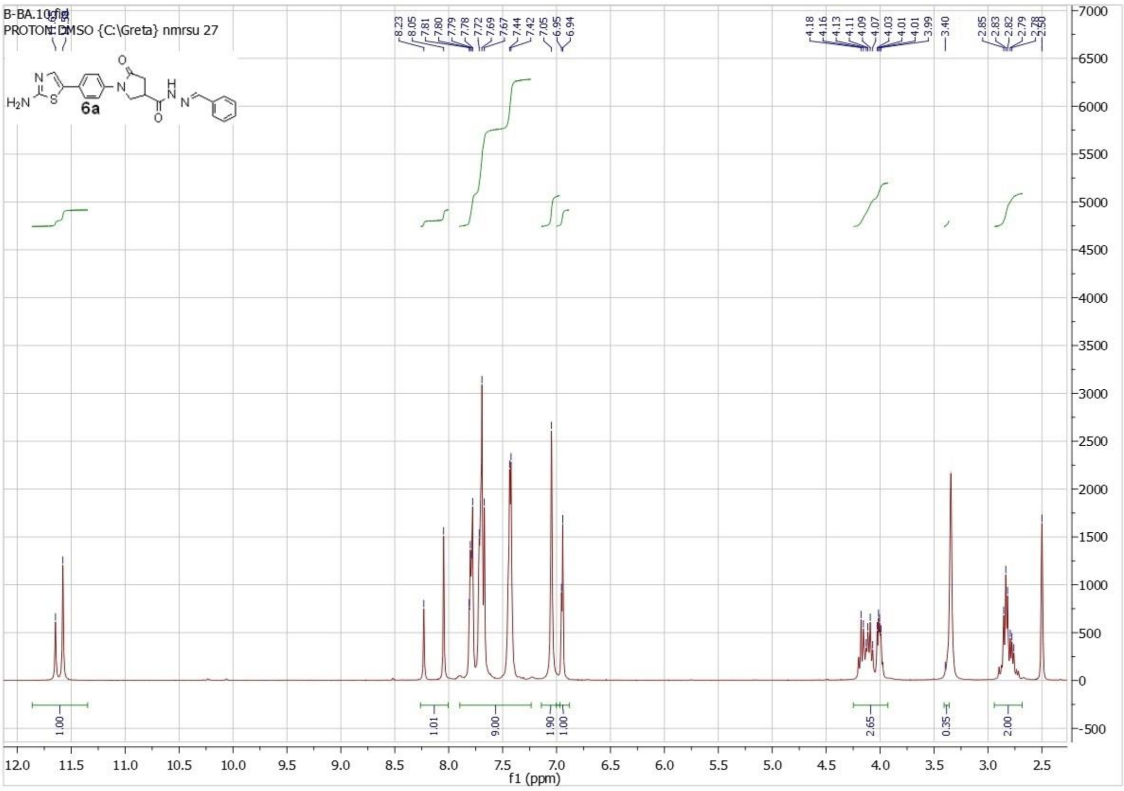

Figure S17: ^{1}H NMR of compound **6 a**

3-(Benzylidenehydrazinocarbonyl)-1-(4-(2-phenylthiazol-5-yl)phenyl)-5-oxopyrrolidine (6 b)

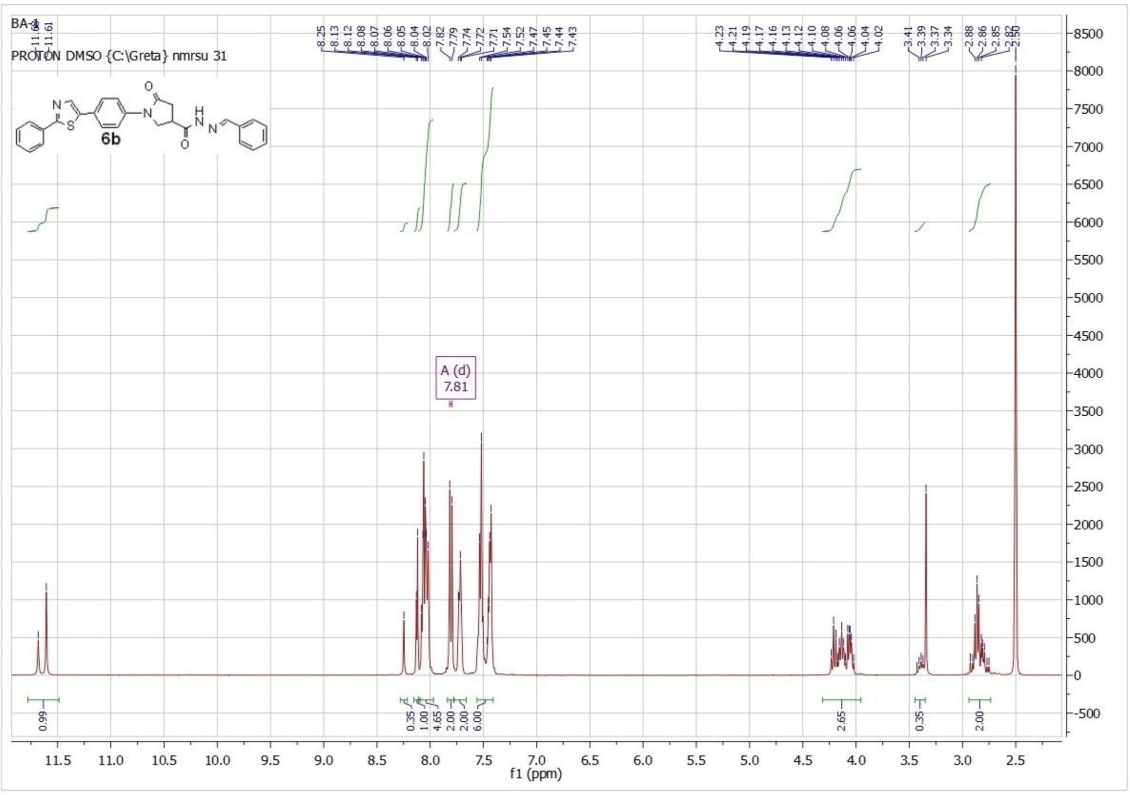

Figure S18: ^{1}H NMR of compound **6 b**

1-(4-(2-Aminothiazol-5-yl)phenyl)-3-(4-chlorobenzylidenehydrazinocarbonyl)-5-oxopyrrolidine (7 a)

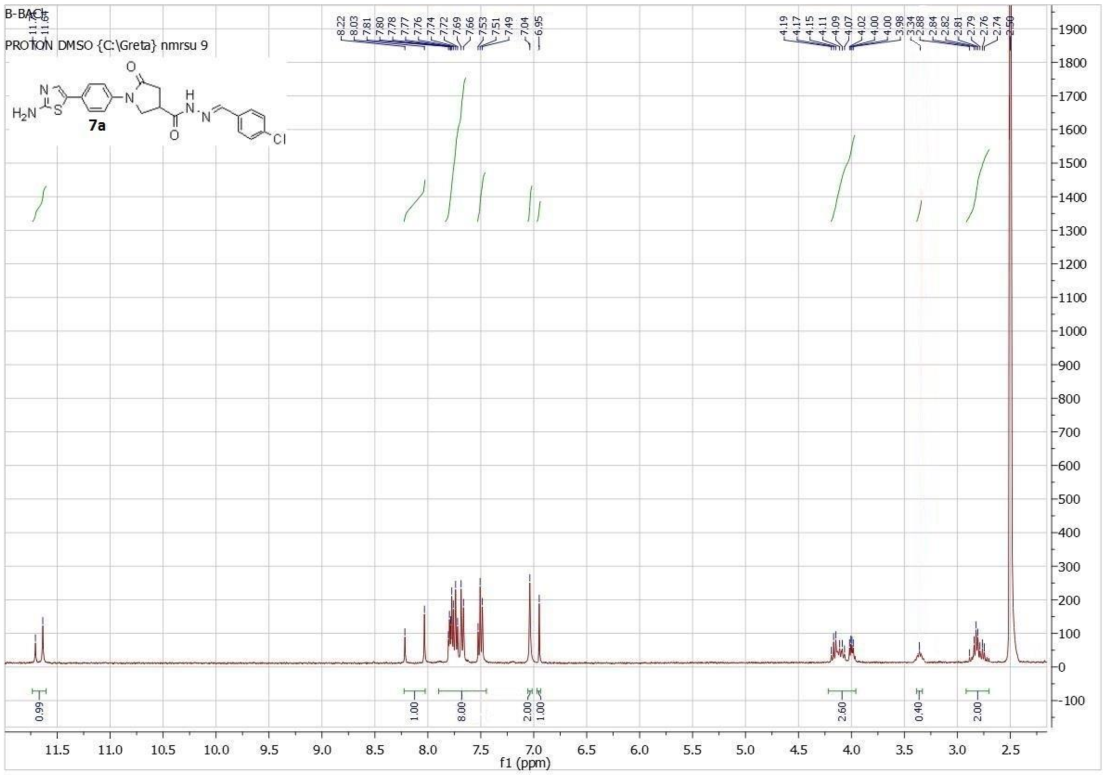

Figure S19: ^{1}H NMR of compound **7 a**

3-(3-Chlorobenzylidenehydrazinocarbonyl)-1-(4-(2-phenylthiazol-5-yl)phenyl)-5-oxopyrrolidine (7 b)

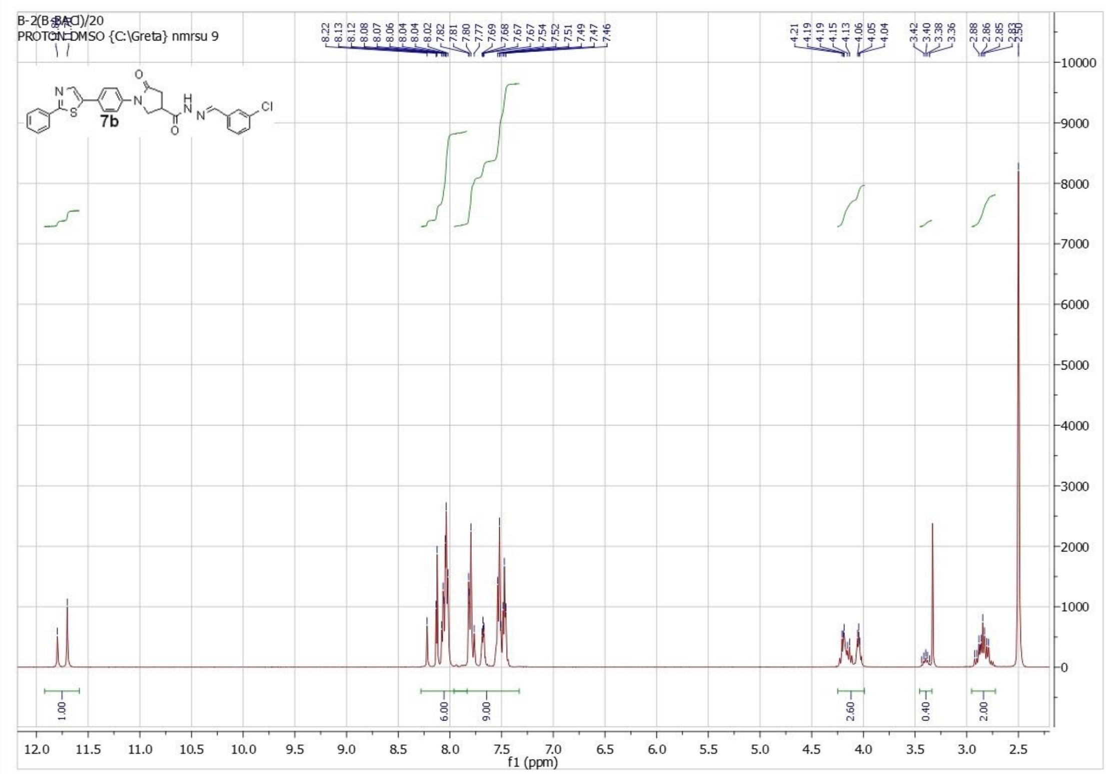

Figure S20: ^{1}H NMR of compound **7 b**

1-(4-(2-Aminothiazol-5-yl)phenyl)-3-(4-nitrobenzylidenehydrazinocarbonyl)-5-oxopyrrolidine (8 a)

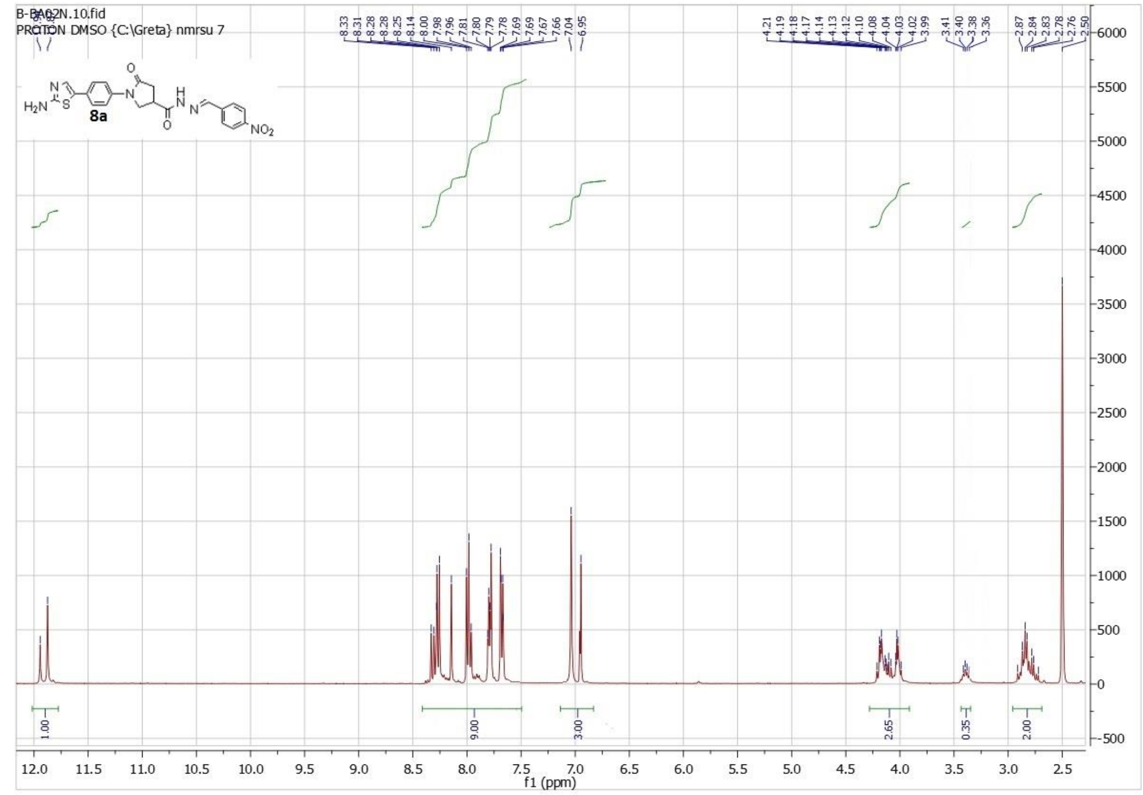

Figure S21: ^{1}H NMR of compound **8 a**

3-(4-Nitrobenzylidenehydrazinocarbonyl)-1-(4-(2-phenylthiazol-5-yl)phenyl)-5-oxopyrrolidine (8 b)

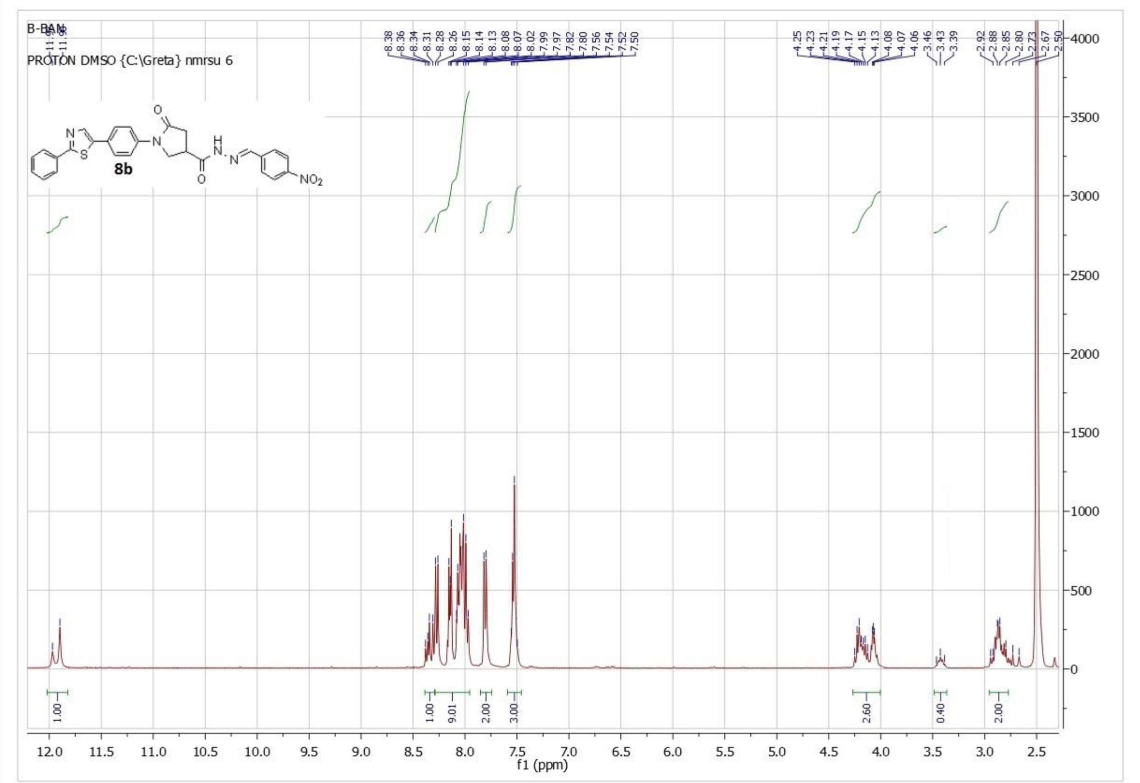

Figure S22: ^{1}H NMR of compound **8 b**

3-(4-Fluorobenzylidenehydrazinocarbonyl)-1-(4-(2-phenylthiazol-5-yl)phenyl)-5-oxopyrrolidine (9 b)

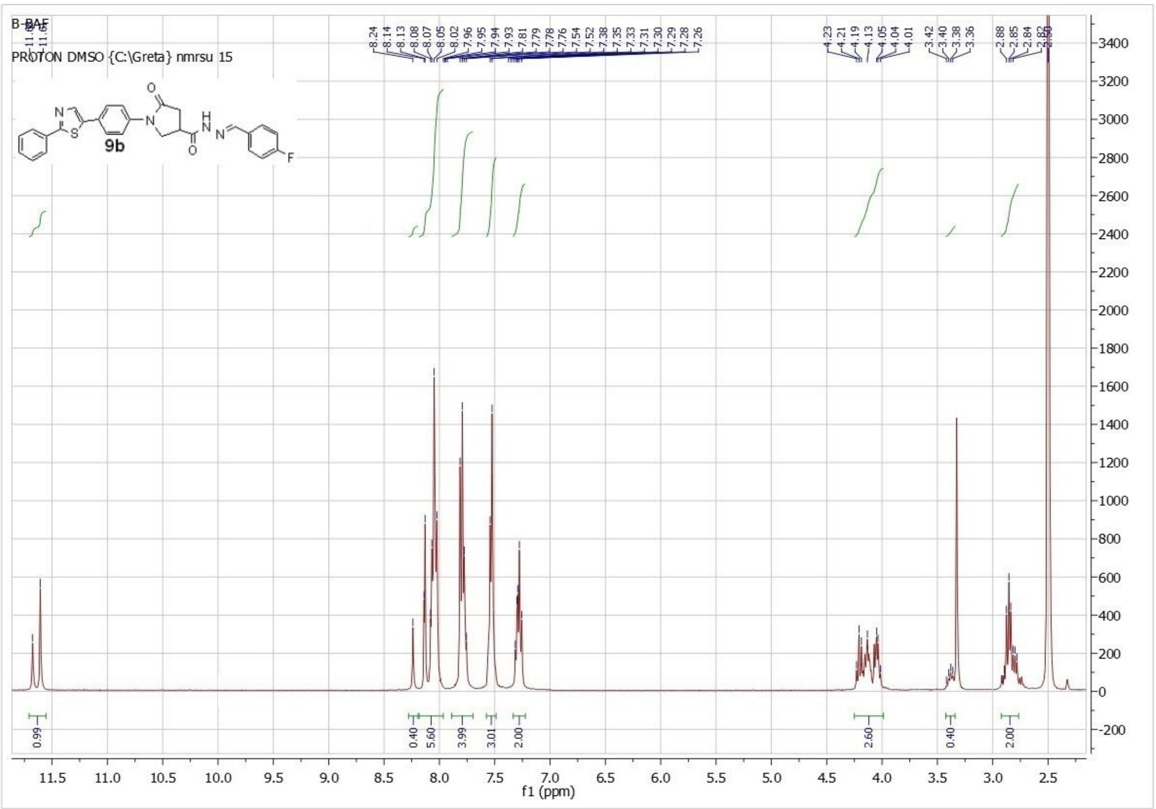

Figure S23: ^{1}H NMR of compound **9 b**

5-Oxo-1-(4-(2-phenylthiazol-5-yl)phenyl)-N'-(thiophen-2-ylmethylene)pyrrolidine-3-carbohydrazide (10 b)

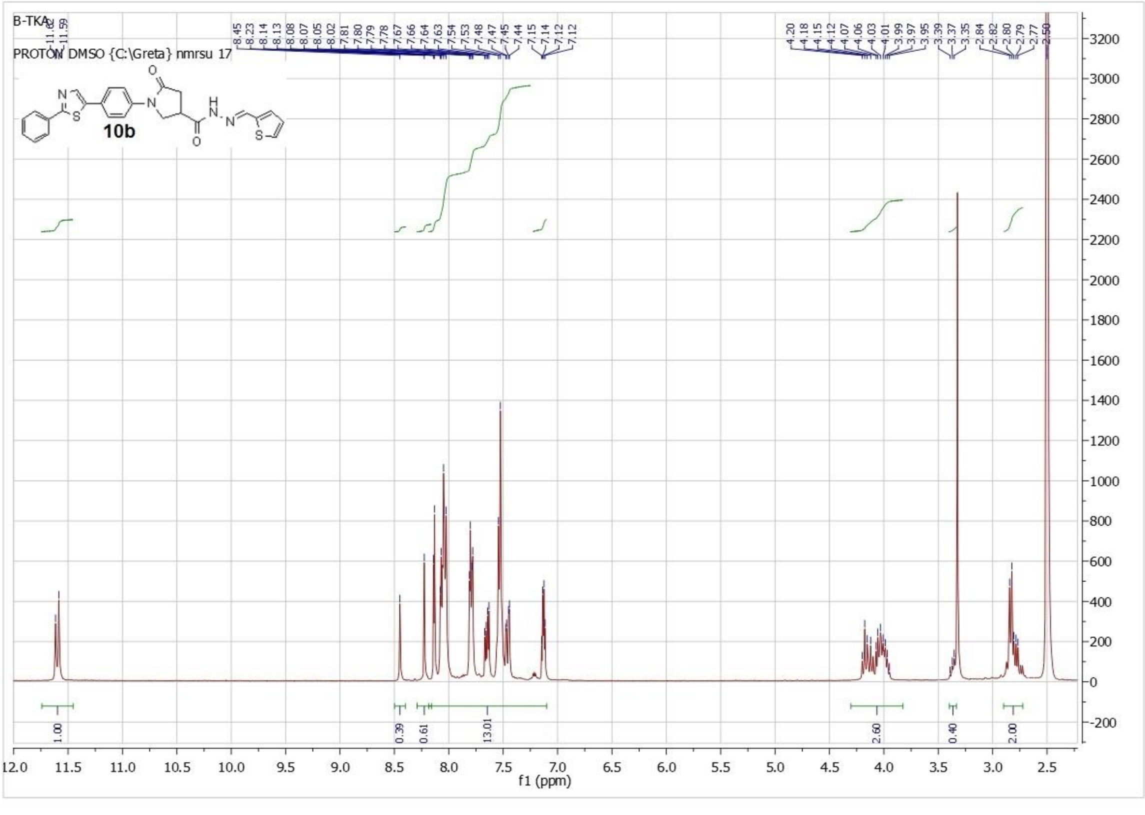

Figure S24: ^{1}H NMR of compound **10 b**

5-Oxo-1-(4-(2-phenylthiazol-5-yl)phenyl)-*N*'-(pyridin-3-ylmethylene)pyrrolidine-3-carbohydrazide (11 b)

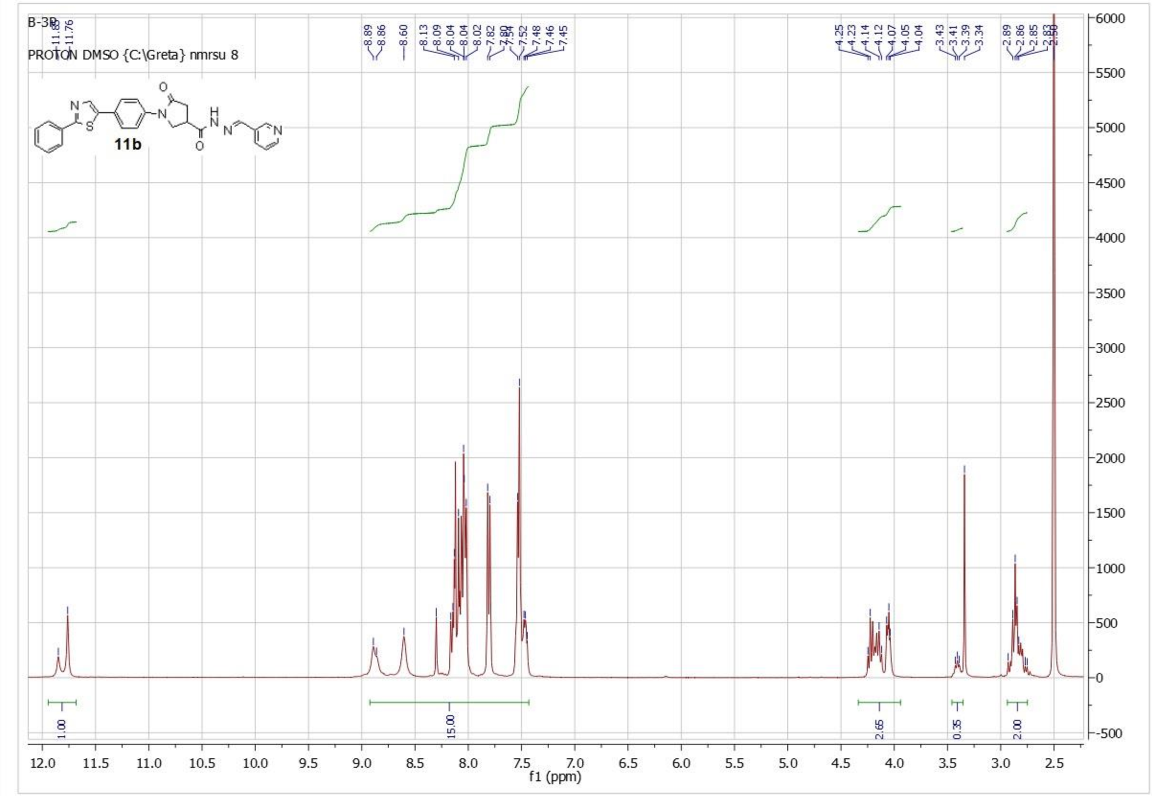

Figure S25: ^{1}H NMR of compound **11 b**

5-Oxo-1-(4-(2-phenylthiazol-5-yl)phenyl)-*N*'-(pyridin-4-ylmethylene)pyrrolidine-3-carbohydrazide (12 b)

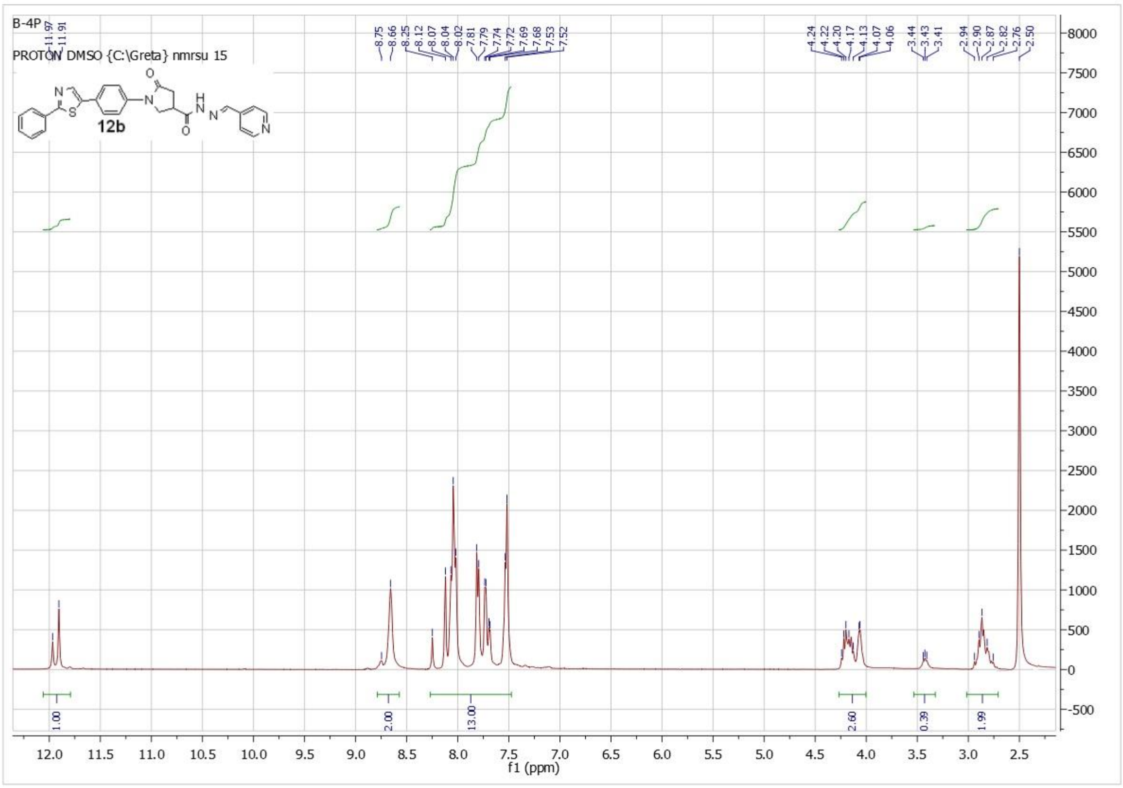

Figure S26: ^{1}H NMR of compound **12 b**

1-(4-(2-Aminothiazol-5-yl)phenyl)-*N*-(2,5-dimethyl-1*H*-pyrrol-1-yl)-5-oxopyrrolidine-3-carboxamide (13a)

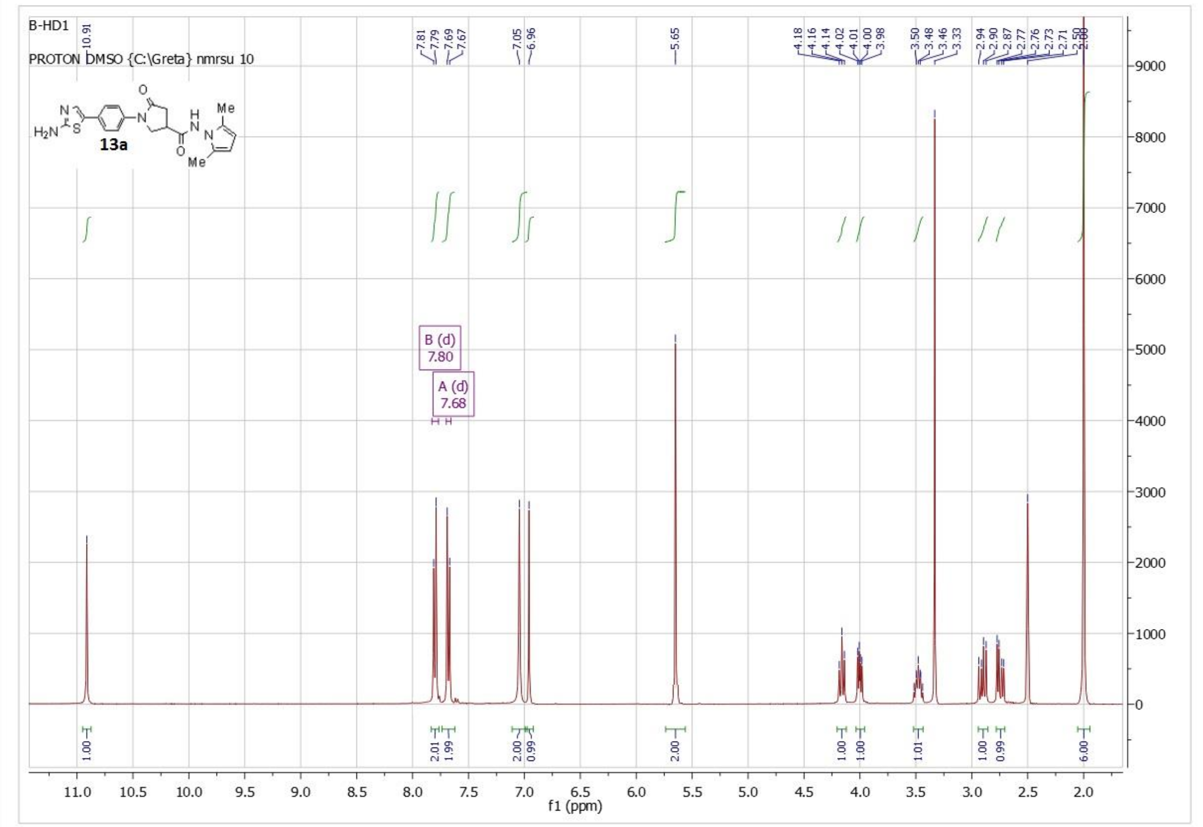

Figure S27: ^{1}H NMR of compound **13 a**

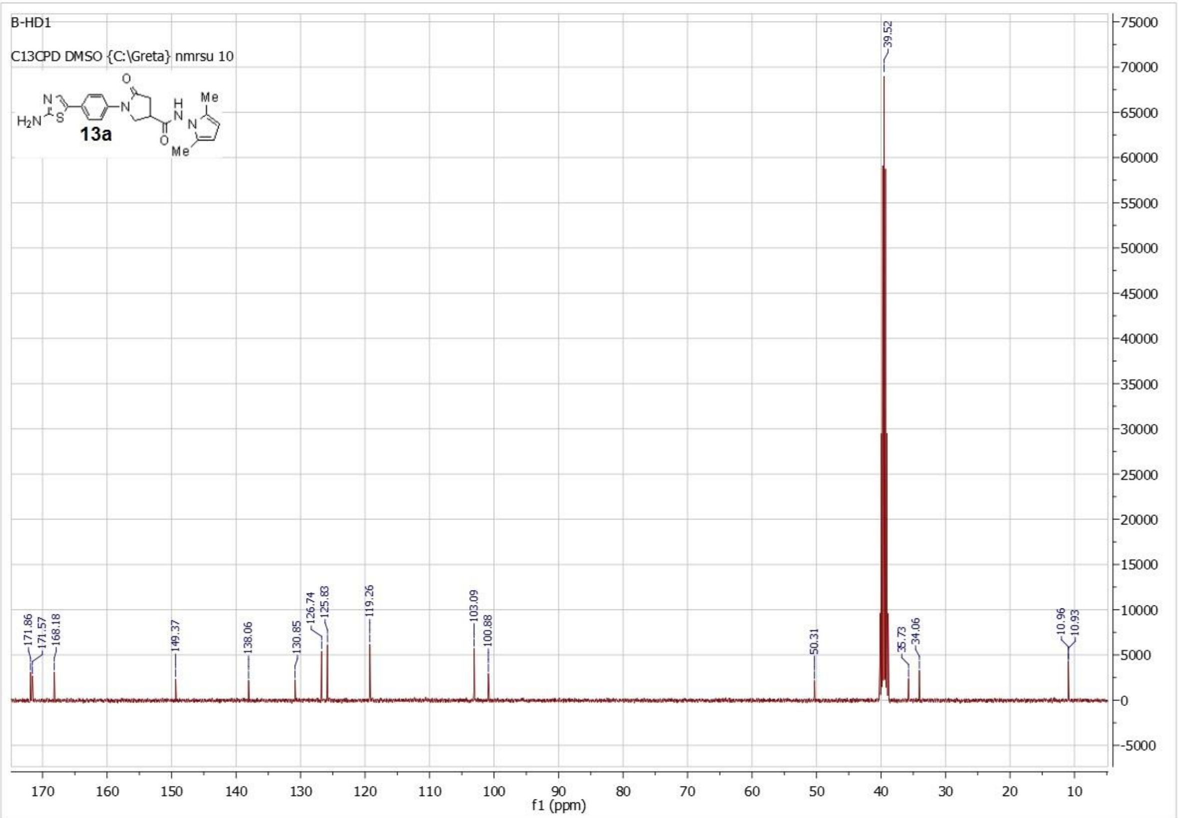

Figure S28: ^{13}C NMR of compound **13 a**

N-(2,5-Dimethyl-1*H*-pyrrol-1-yl)-5-oxo-1-(4-(2-phenylthiazol-5-yl)phenyl)pyrrolidine-3-carboxamide (13 b)

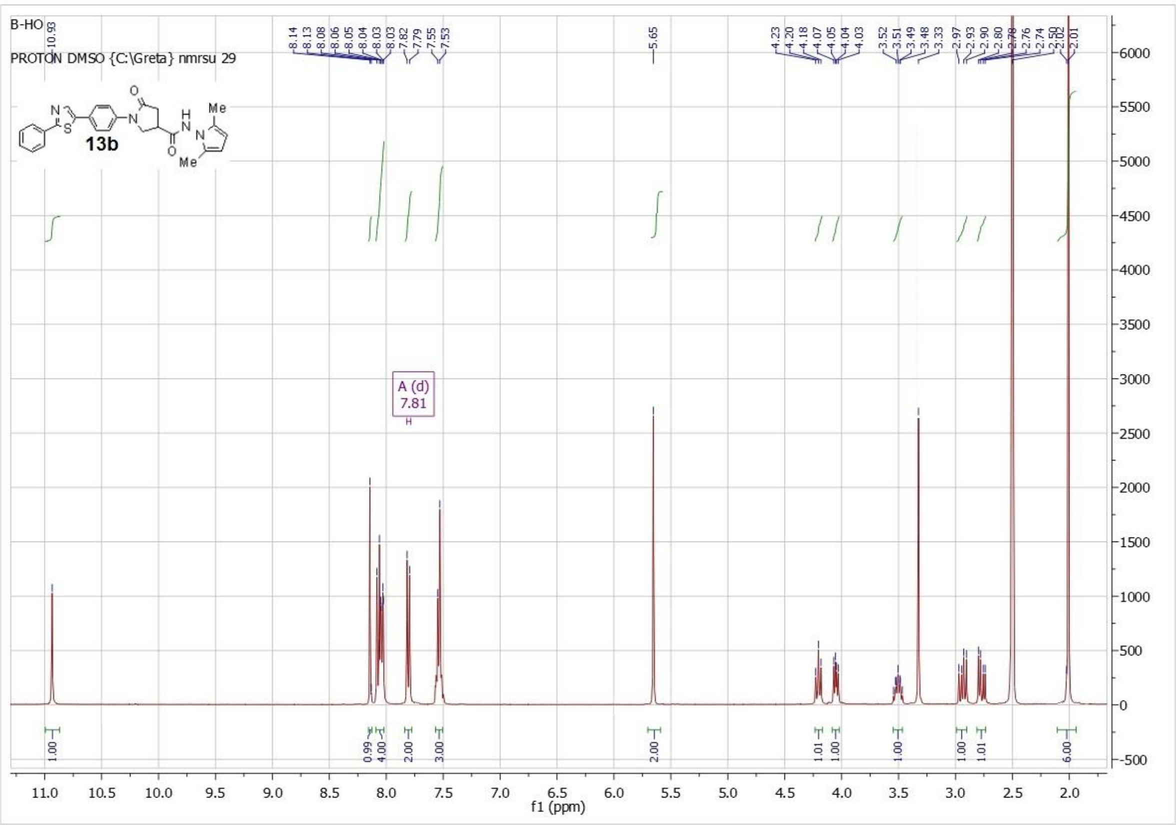

Figure S29: ^{1}H NMR of compound **13 b**

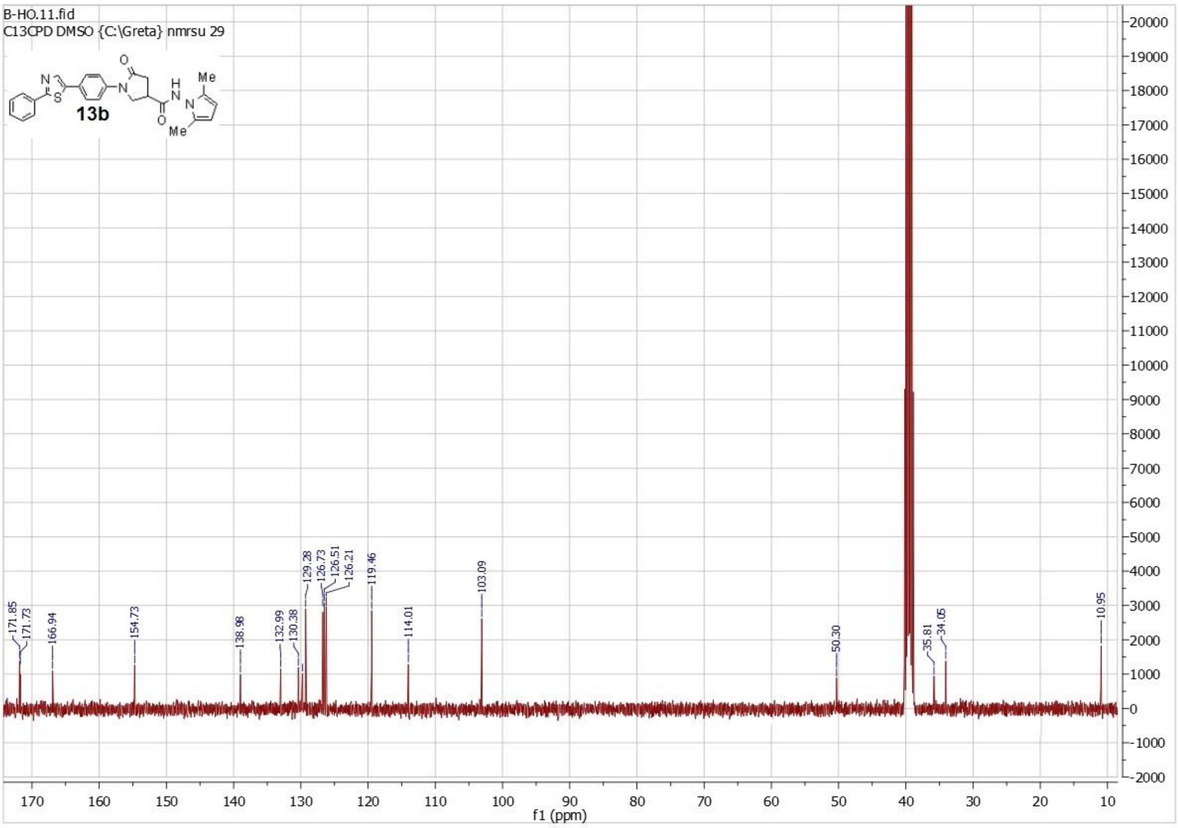

Figure S30: ^{13}C NMR of compound **13 b**

5-Oxo-1-(5-oxo-1-(4-(2-phenylthiazol-5-yl)phenyl)pyrrolidine-3-carboxamido)pyrrolidine-3-carboxylic acid (14 b)

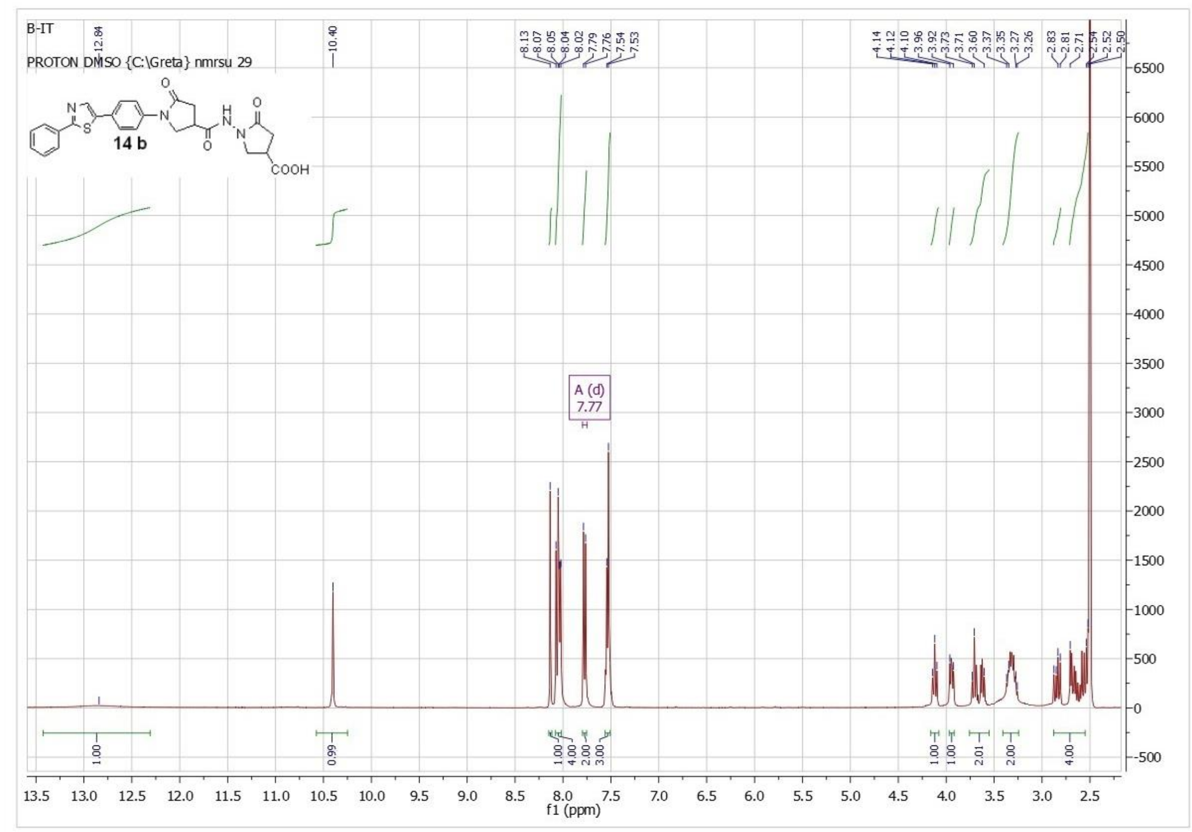

Figure S31: ^{1}H NMR of compound **14 b**

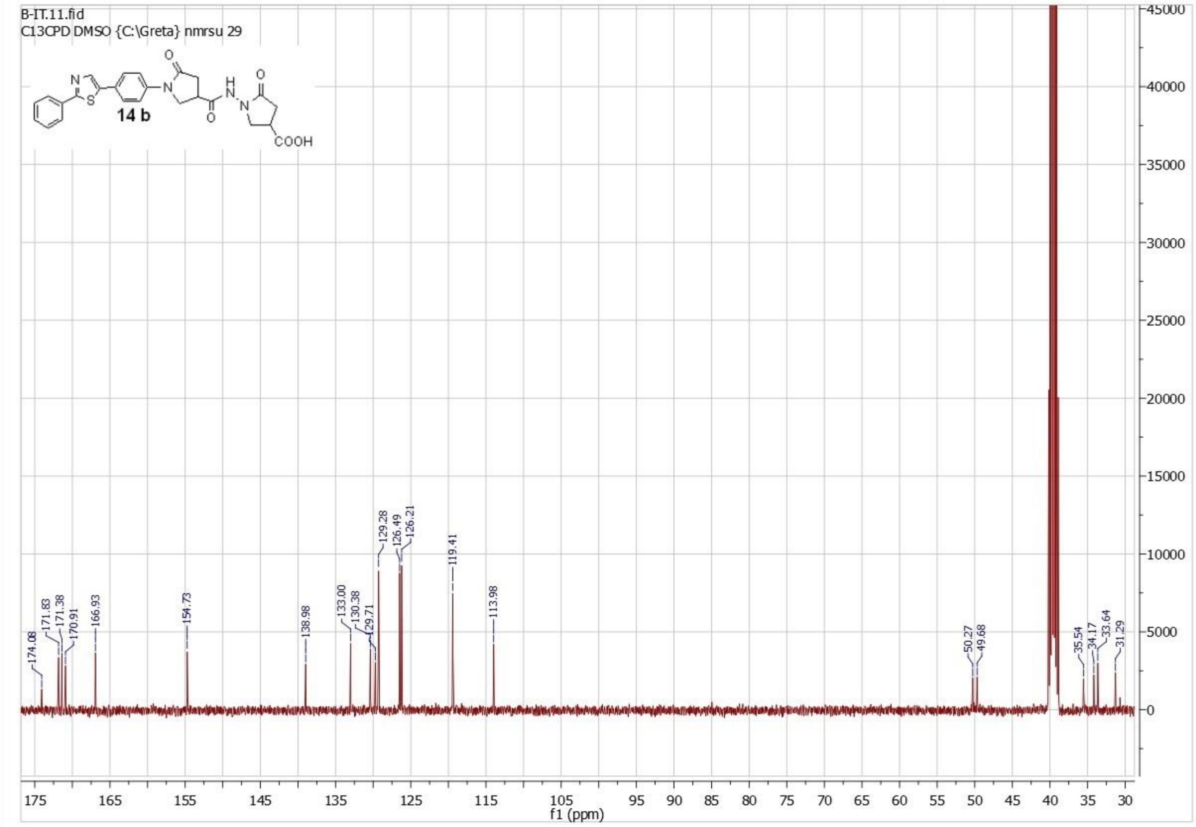

Figure S32: ^{13}C NMR of compound **14 b**

2-(5-Oxo-1-(4-(2-phenylthiazol-5-yl)phenyl)pyrrolidine-3-carbonyl)-*N*-phenylhydrazine-1-carbothioamide (15 b)

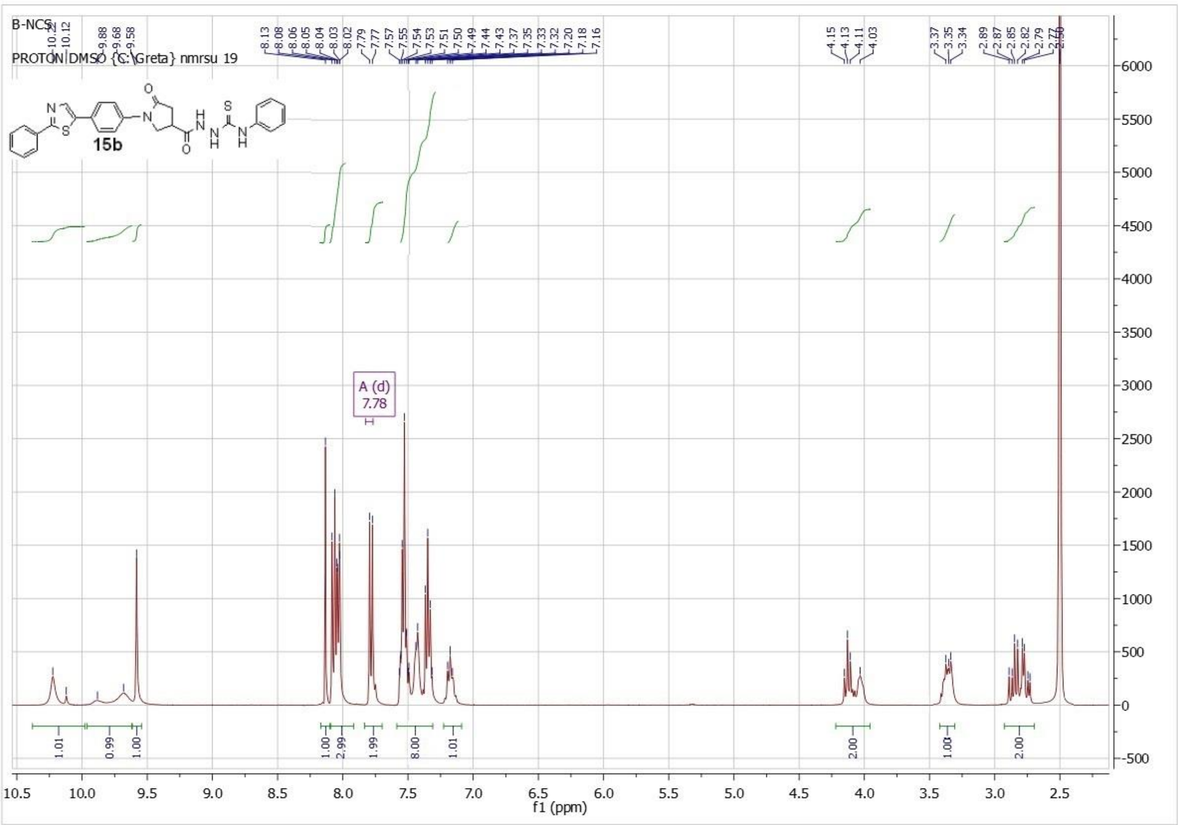

Figure S33: ¹H NMR of compound **15 b**

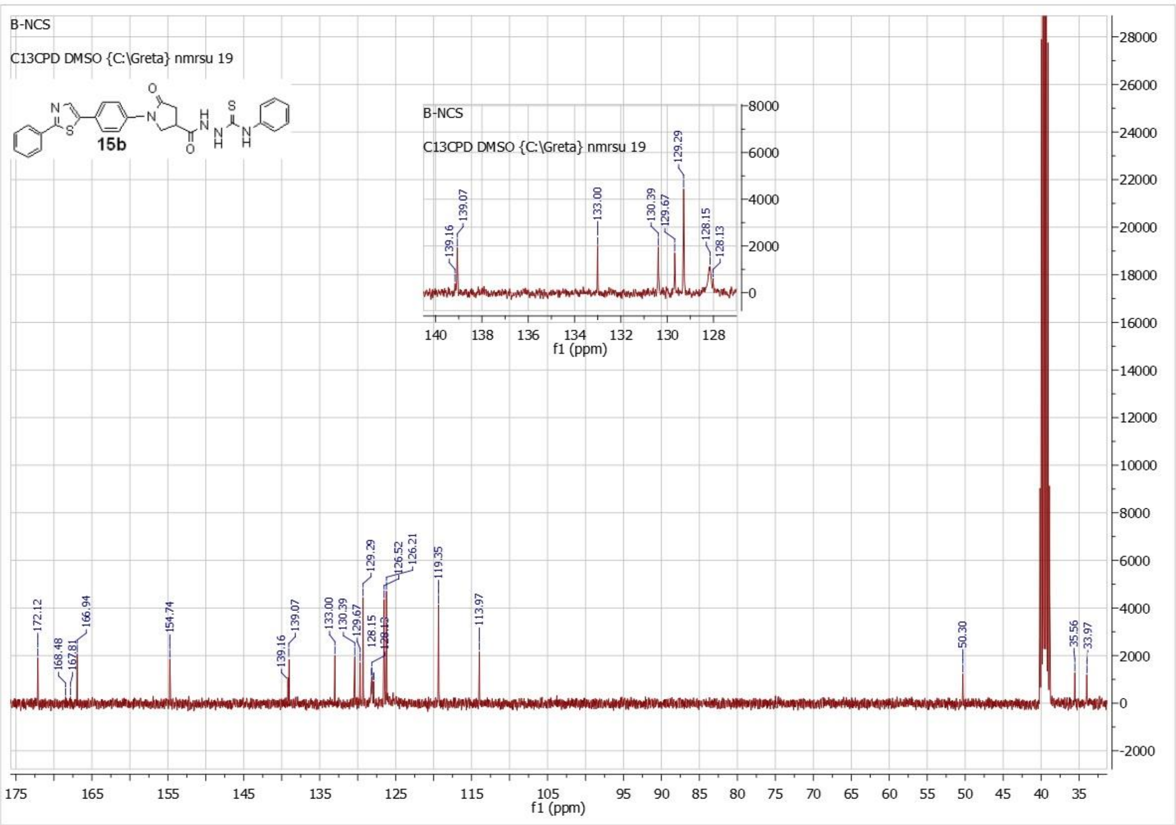

Figure S34: ¹³C NMR of compound **15 b**

3-(4-Phenyl-5-thioxo-4,5-dihydro-1*H*-1,2,4-triazol-3-yl)-1-(4-(2-phenylthiazol-5-yl)phenyl)-5-oxopyrrolidine (16 b)

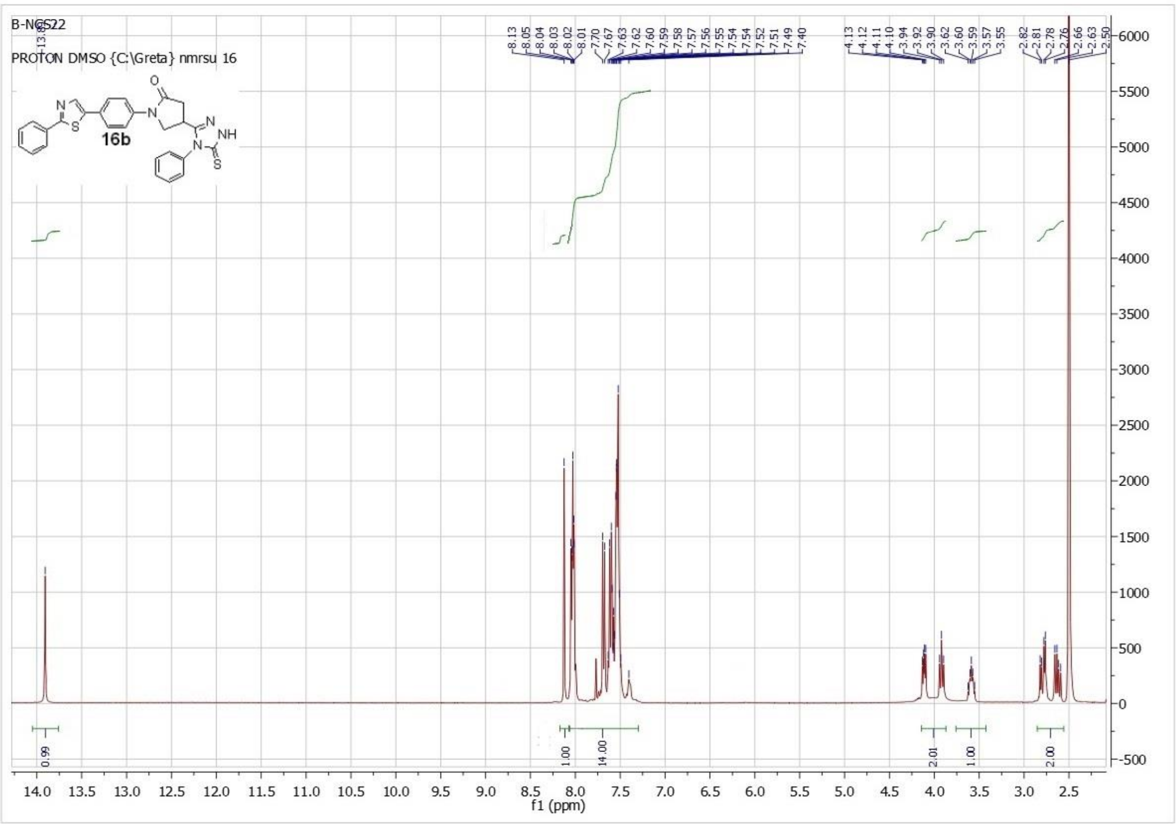

Figure S35: ^{1}H NMR of compound **16 b**

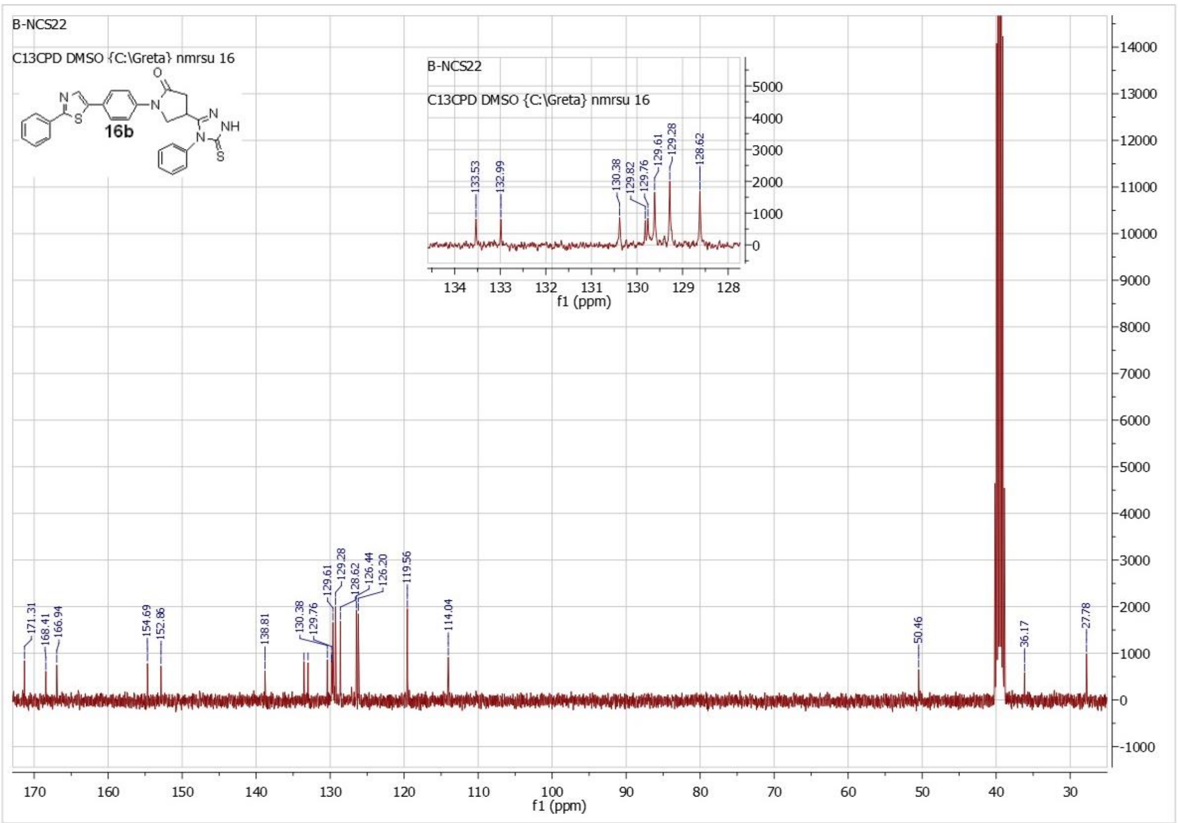

Figure S36: ^{13}C NMR of compound **16 b**

3-(5-Thioxo-4,5-dihydro-1,3,4-oxadiazol-2-yl)-1-(4-(2-phenylthiazol-5-yl)phenyl)-5-oxopyrrolidine (17 b)

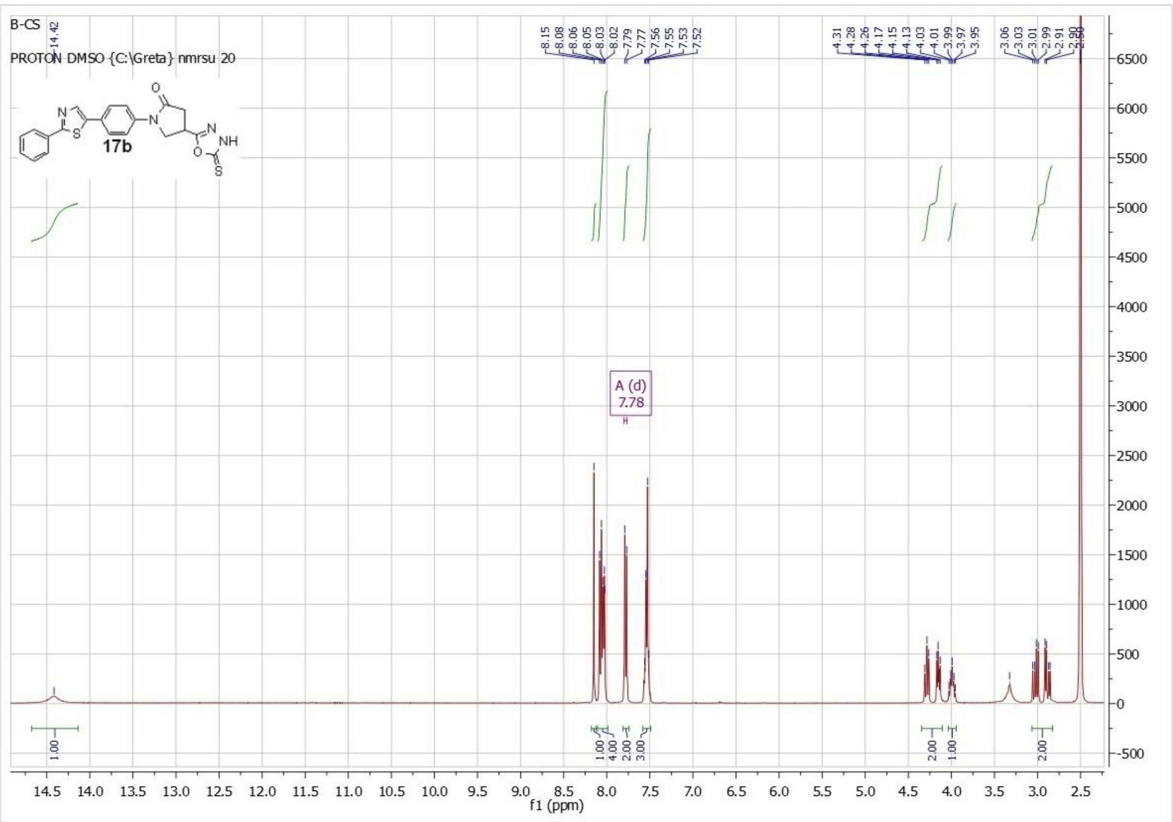

Figure S37: ^{1}H NMR of compound **17 b**

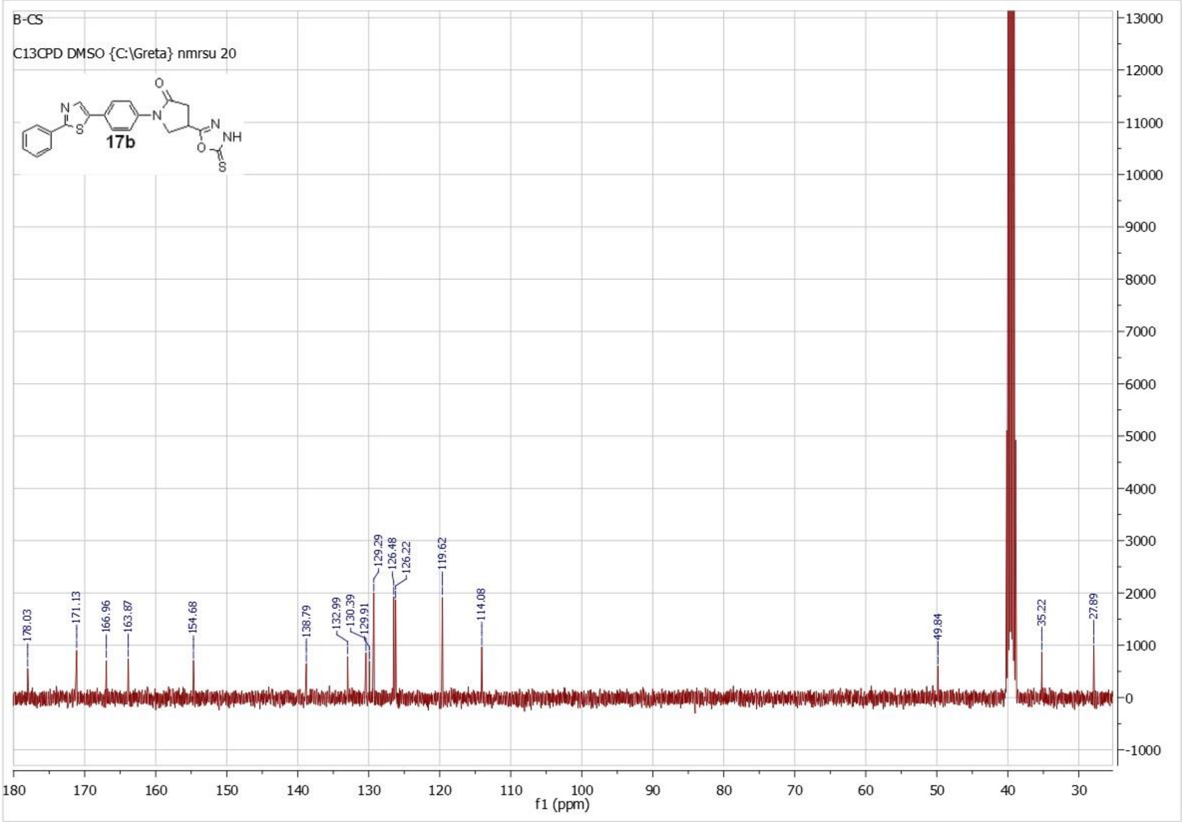

Figure S38: ^{13}C NMR of compound **17 b**

3-(4-Amino-5-thioxo-4,5-dihydro-1*H*-1,2,4-triazol-3-yl)-1-(4-(2-phenylthiazol-5-yl)phenyl)-5-oxopyrrolidine (18 b)

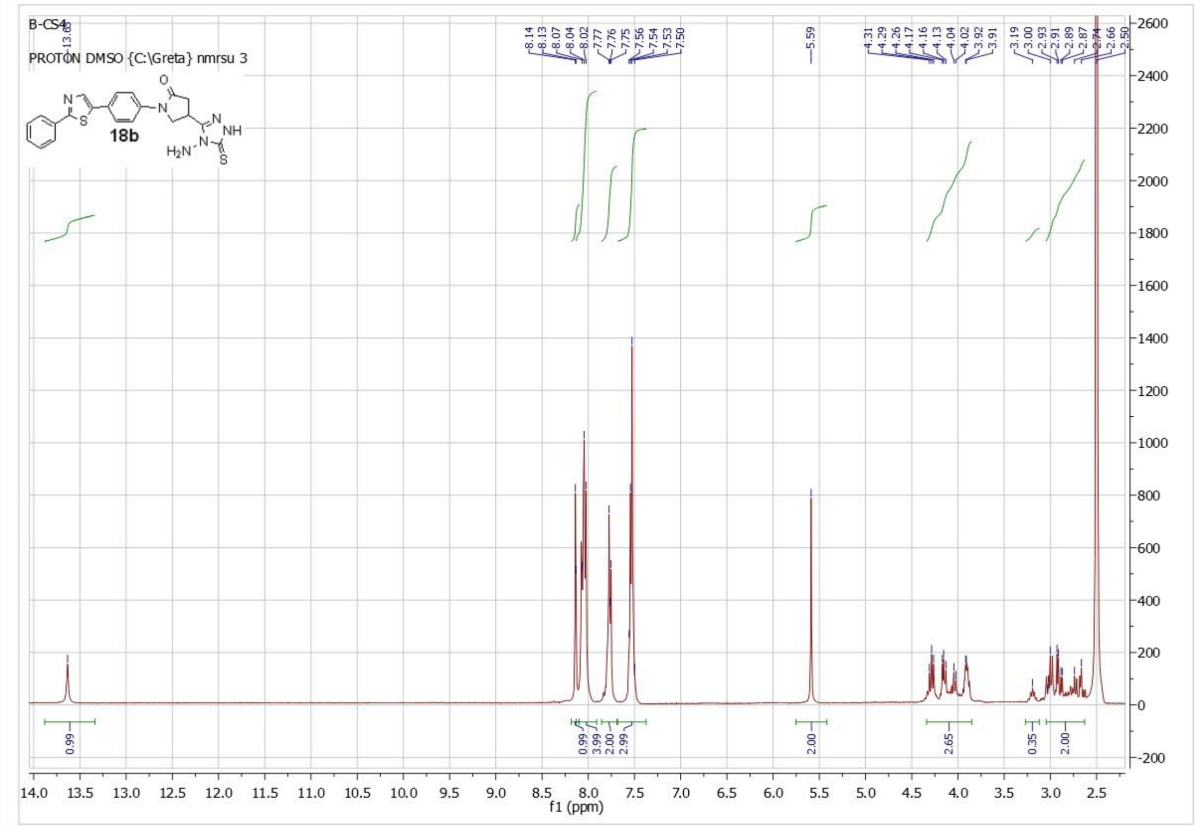

Figure S39: ^{1}H NMR of compound **18 b**

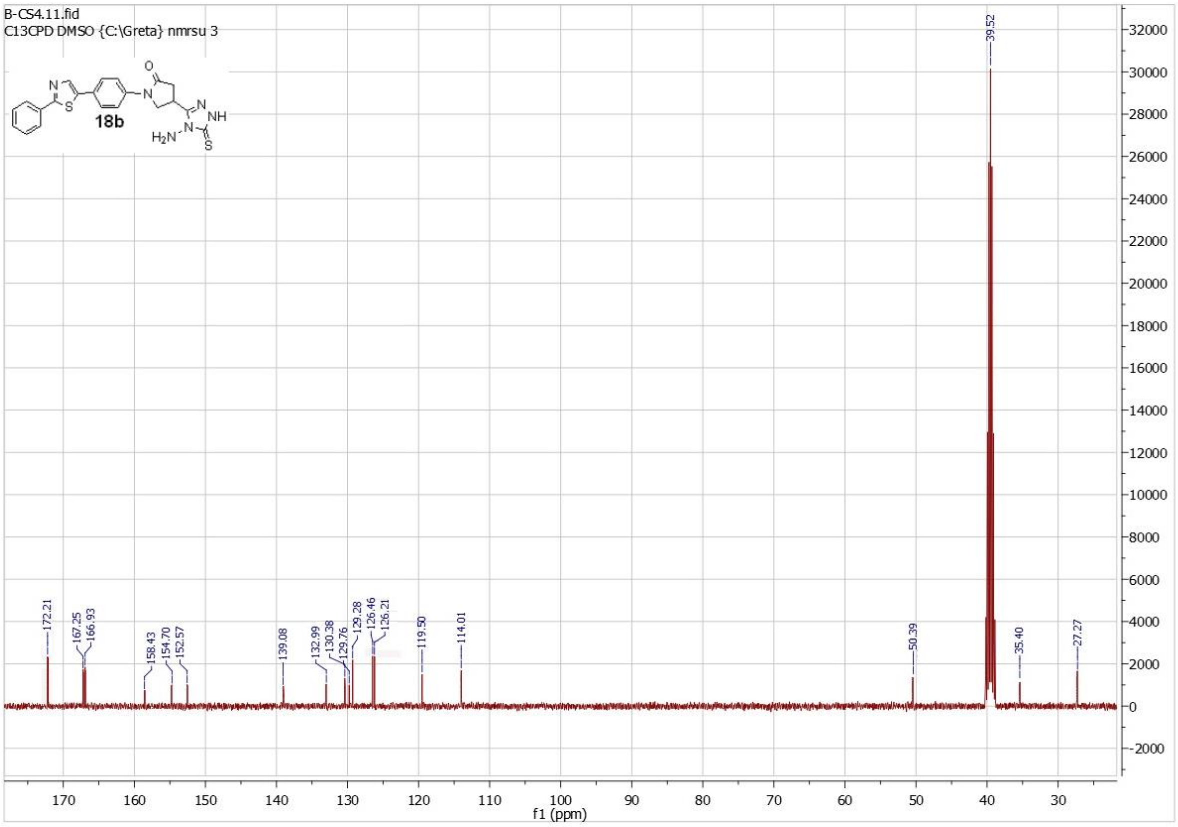

Figure S40: ^{13}C NMR of compound **18 b**

3-(4-(Benzylideneamino)-5-thioxo-4,5-dihydro-1*H*-1,2,4-triazol-3-yl)-1-(4-(2-phenylthiazol-5-yl)phenyl)-5-oxopyrrolidine (19 b)

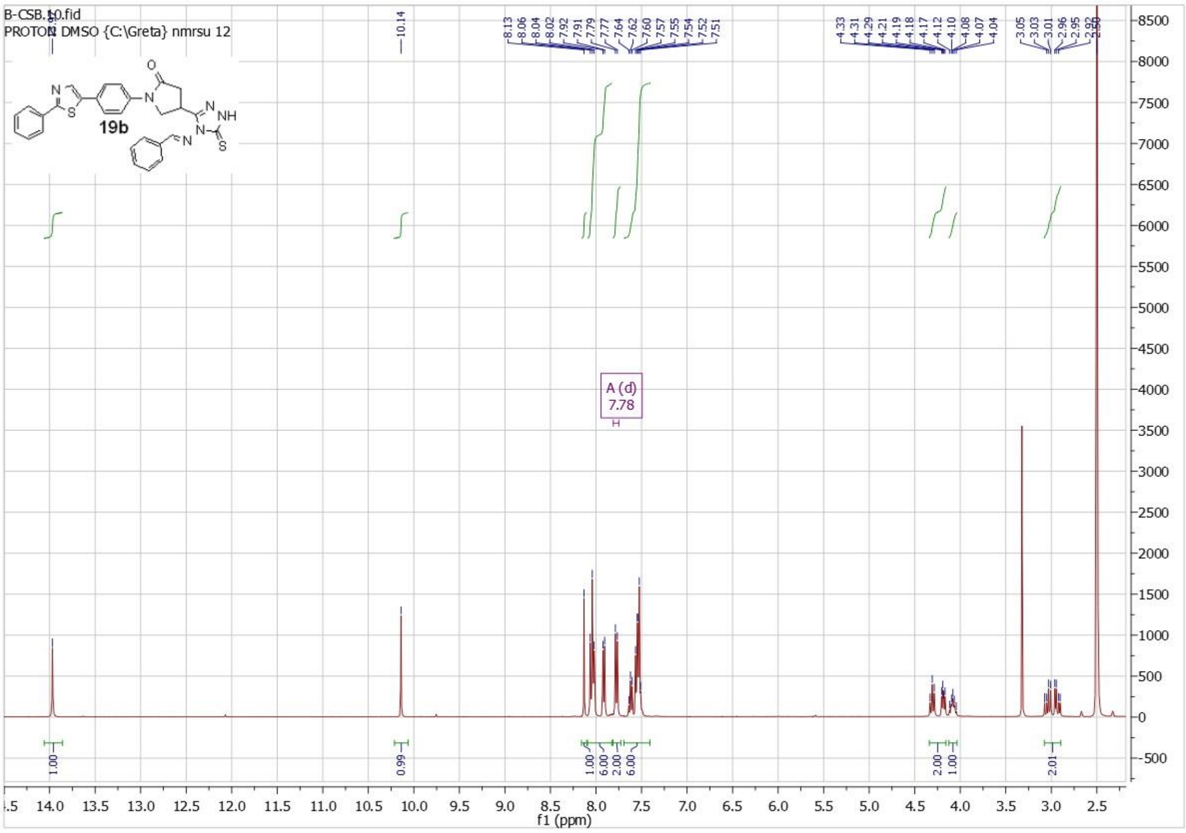

Figure S41: ^{1}H NMR of compound **19 b**

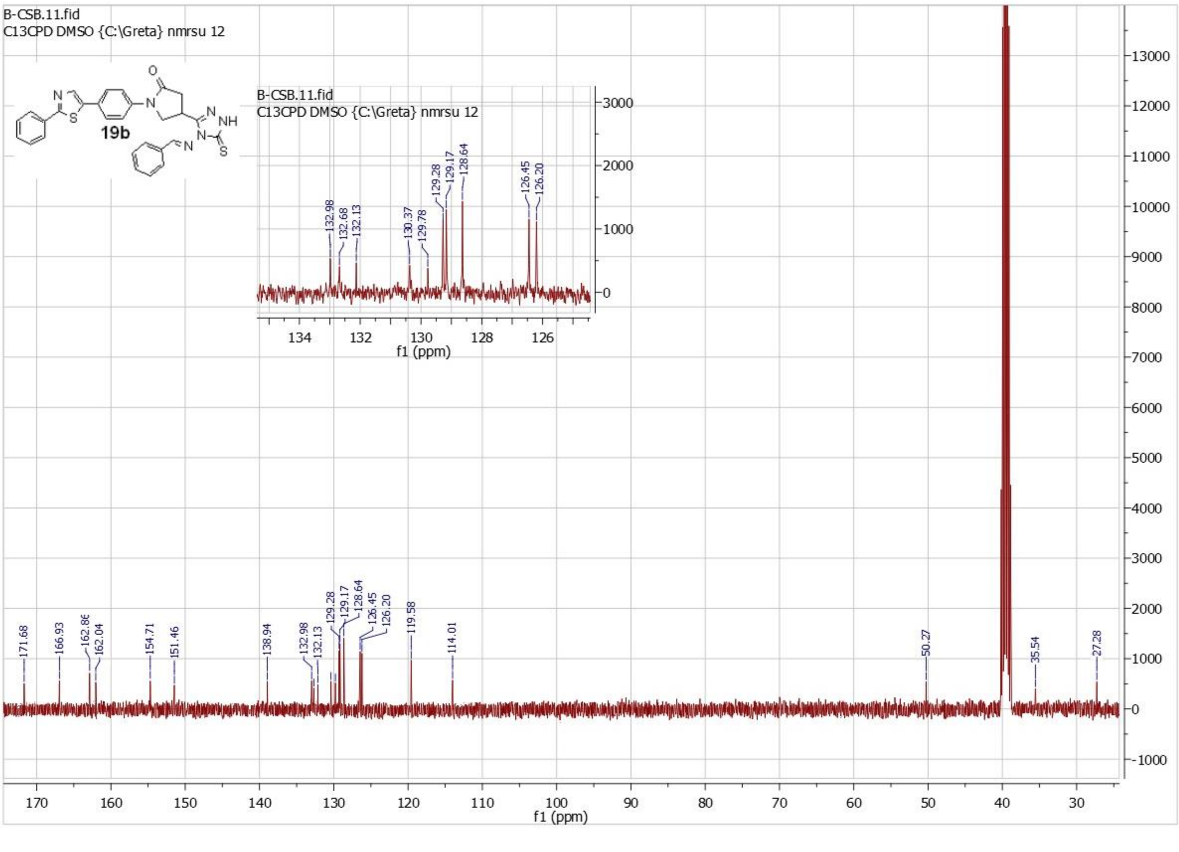

Figure S42: ^{13}C NMR of compound **19 b**

1-(4-(1-((2-Aminophenyl)iminoethyl)phenyl)-5-oxopyrrolidine-3-carboxylic acid (20)

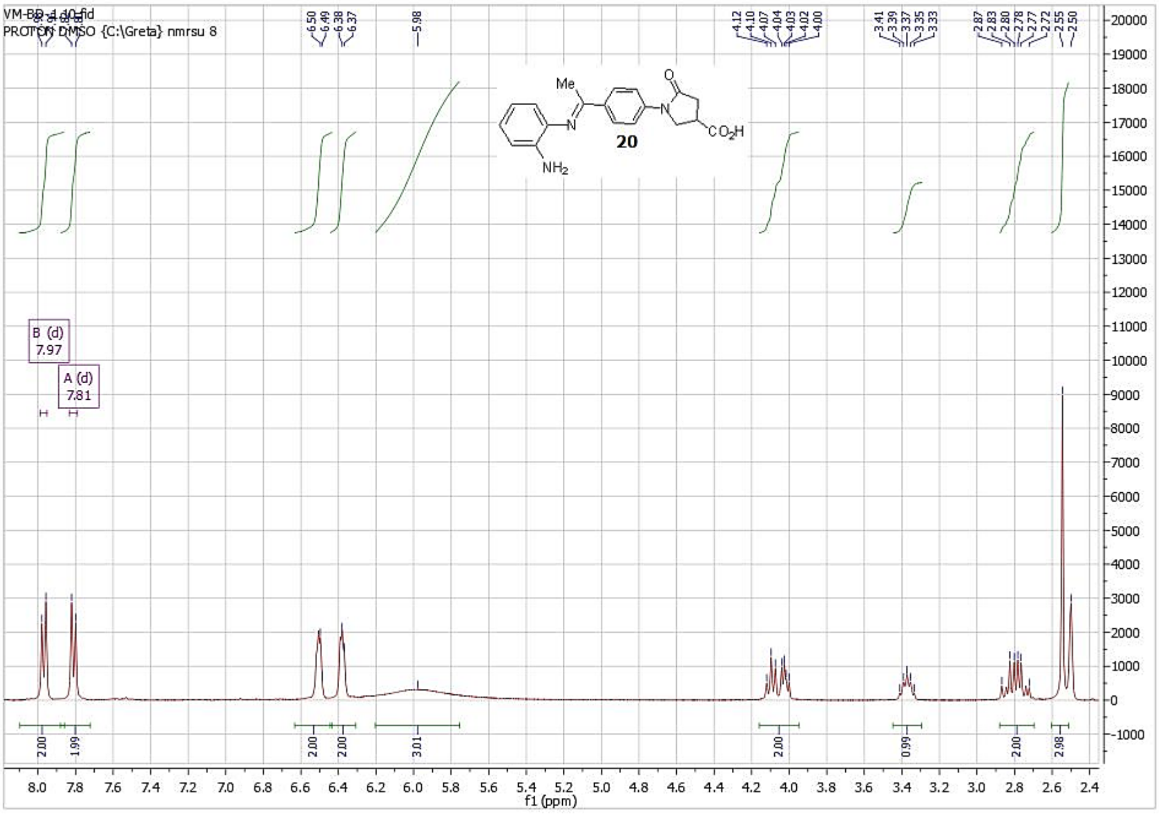

Figure S43: ^{1}H NMR of compound **20**

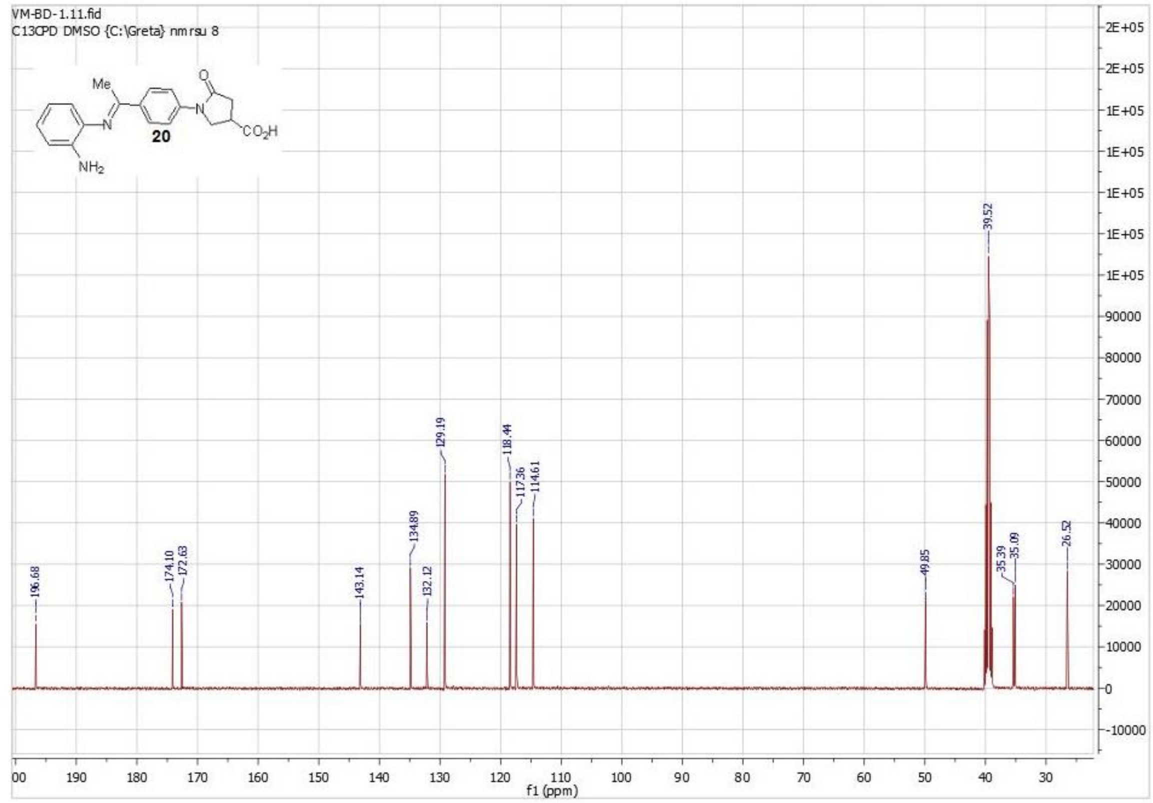

Figure S44: ^{13}C NMR of compound **20**

5-Oxo-1-(4-(quinoxalin-2-yl)phenyl)pyrrolidine-3-carboxylic acid (21)

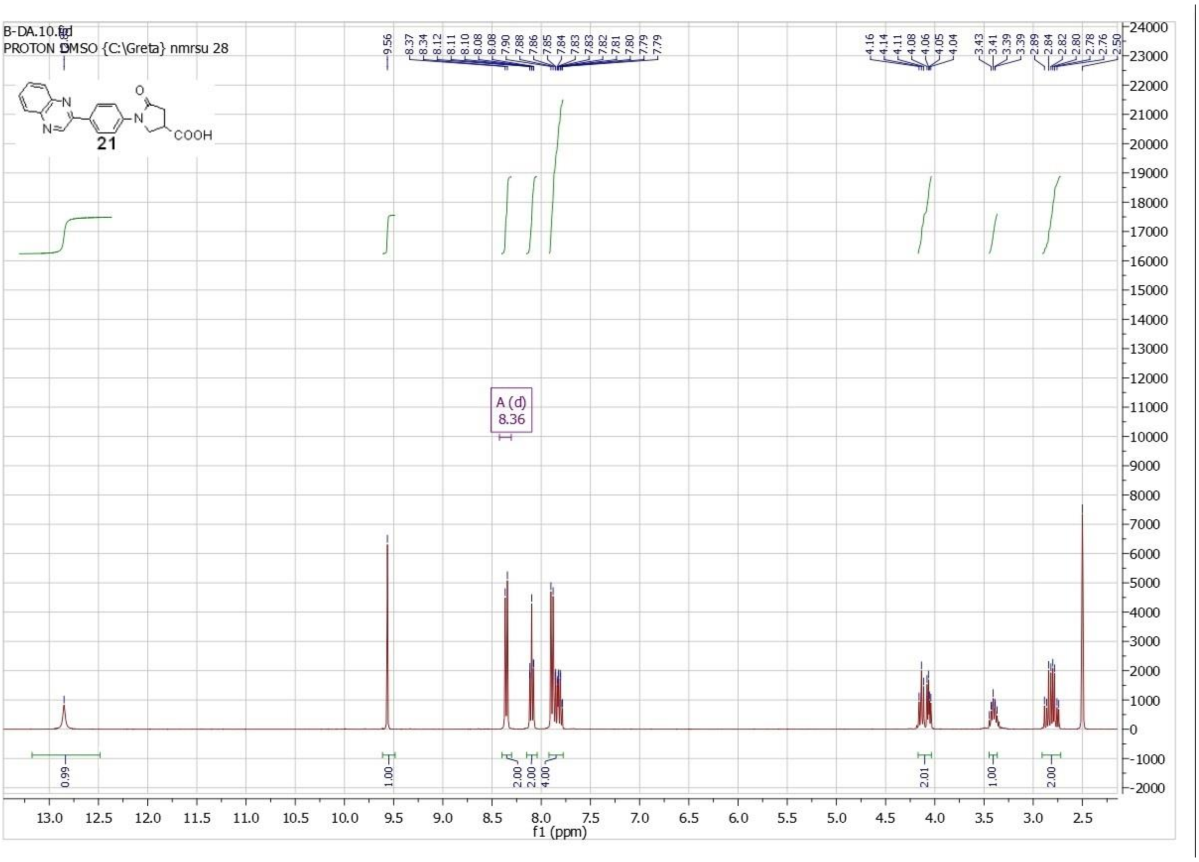

Figure S45: ^{1}H NMR of compound **21**

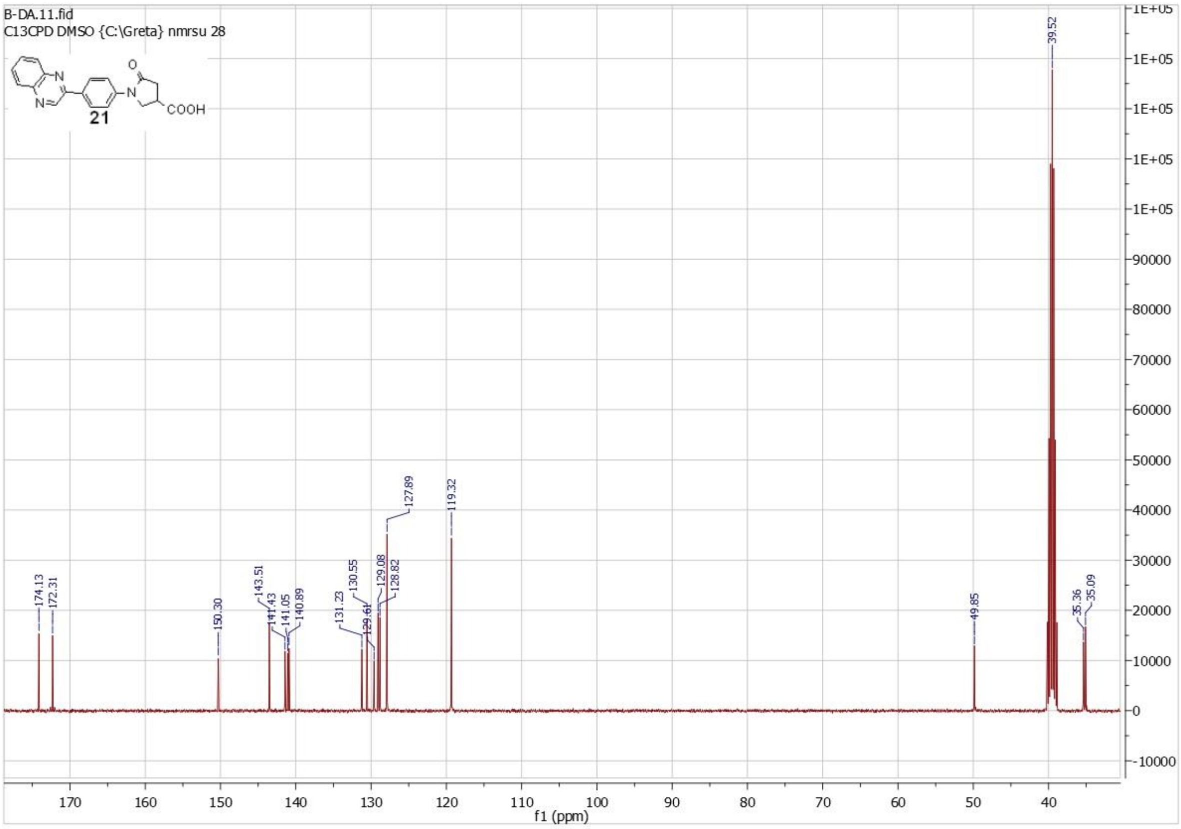

Figure S46: ^{13}C NMR of compound **21**

1-(4-Acetylphenyl)-3-(1*H*-benzimidazol-2-yl)-5-oxopyrrolidine (22)

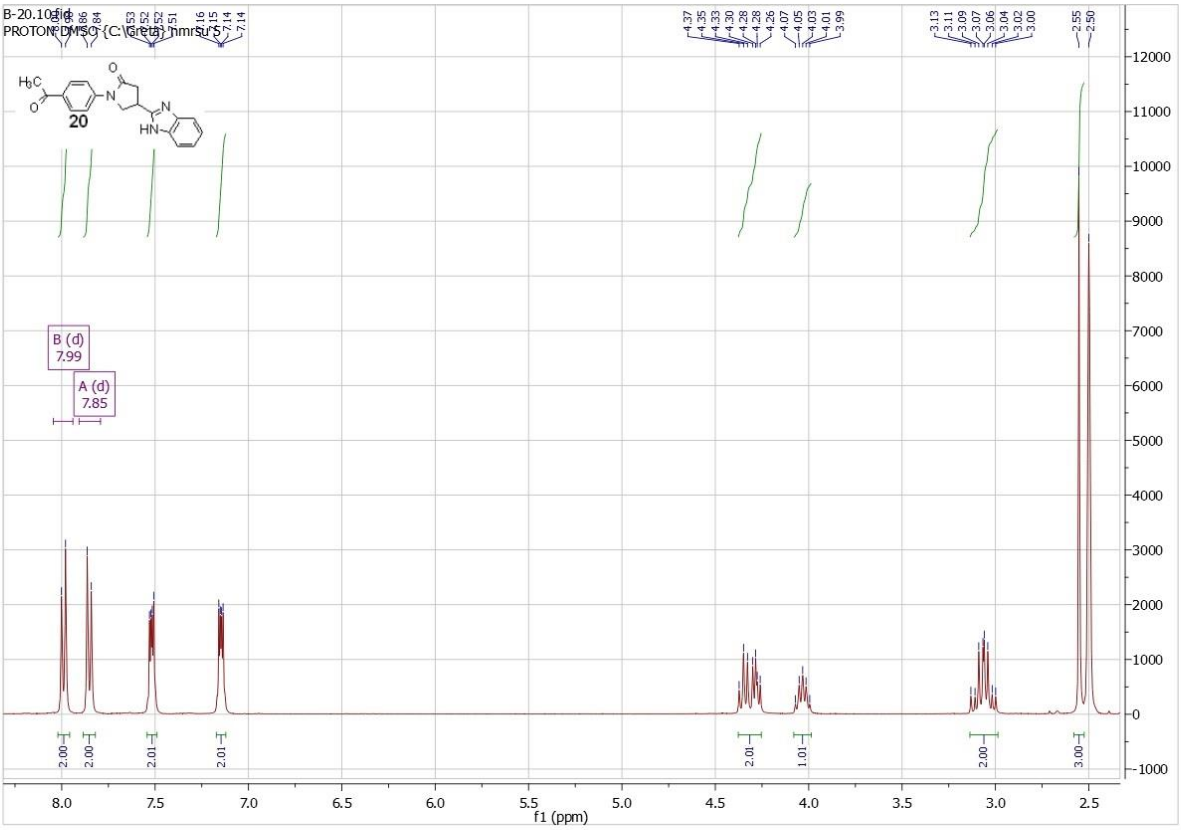

Figure S47: ^{1}H NMR of compound **22**

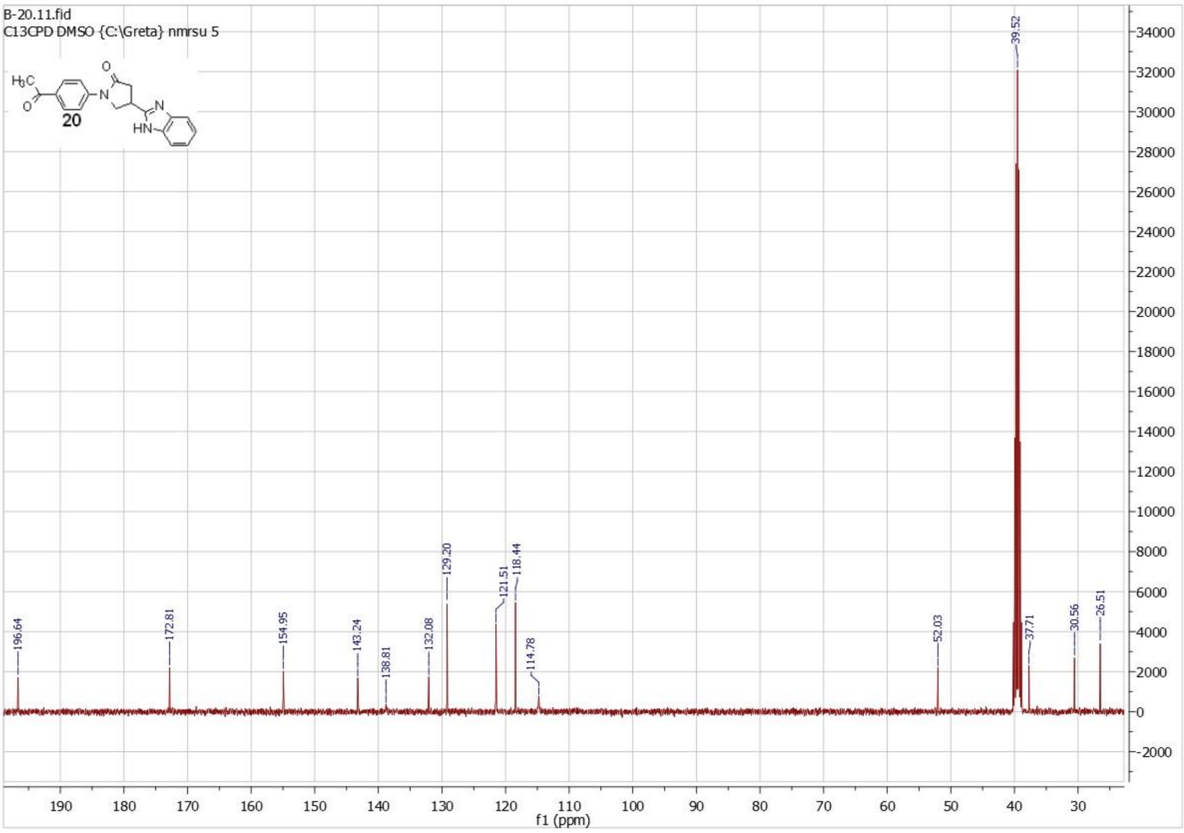

Figure S48: ^{13}C NMR of compound **22**

1-(4-Acetylphenyl)-3-(1-ethyl-1*H*-benzimidazol-2-yl)-5-oxopyrrolidine (23)

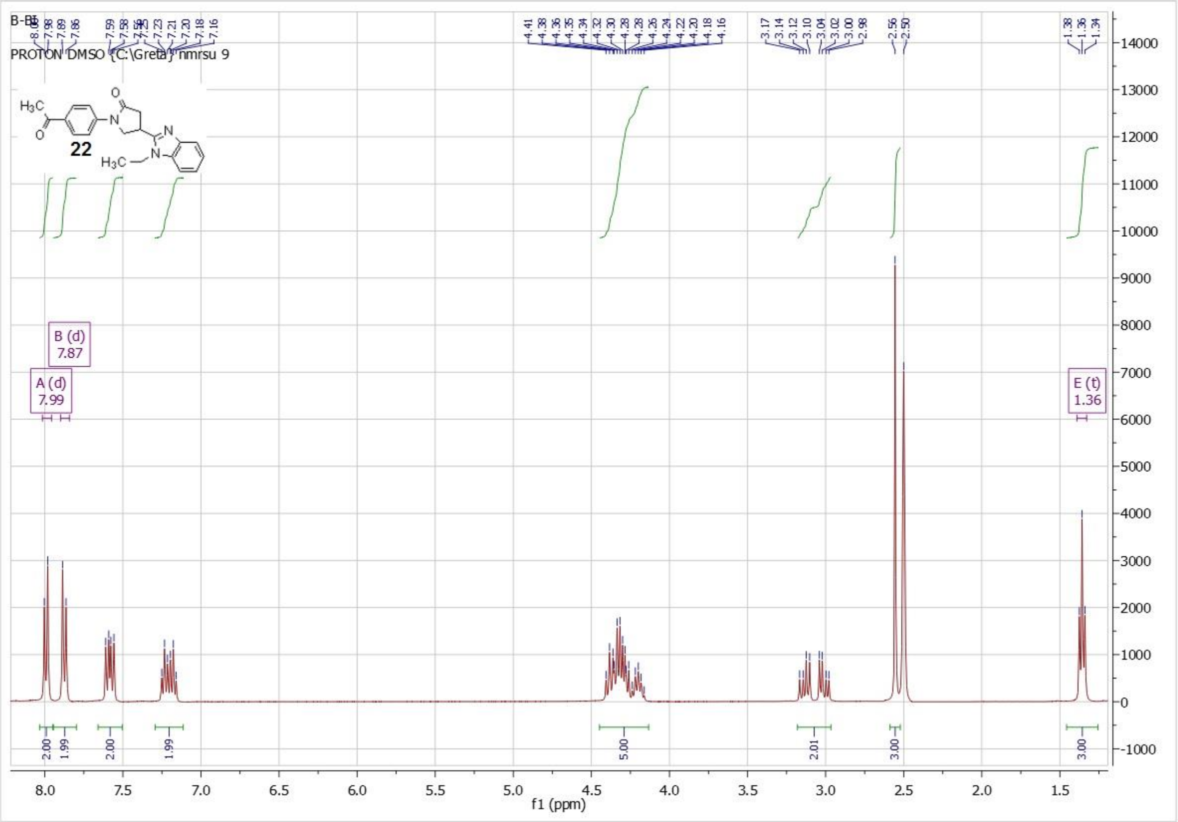

Figure S49: ^{1}H NMR of compound **23**

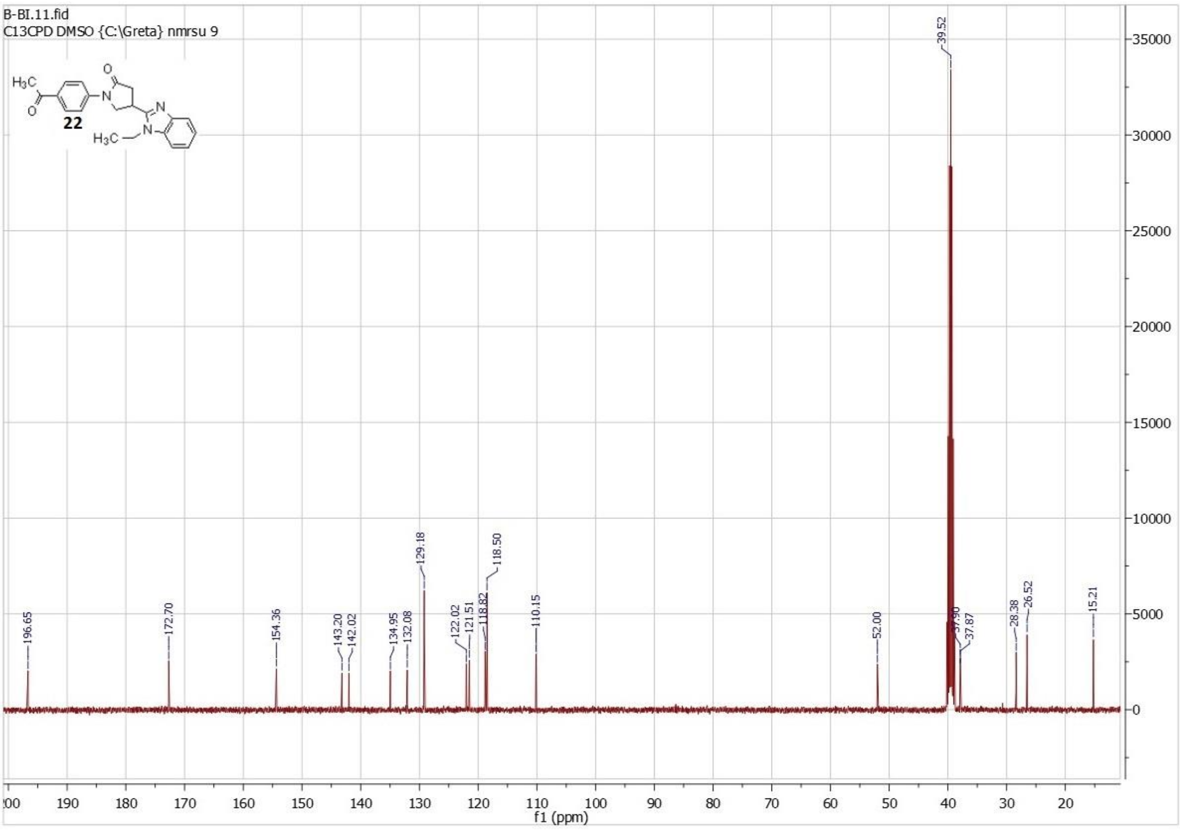

Figure S50: ^{13}C NMR of compound **23**
